# Supplementary material for: Tumor-targeted top1 inhibitor delivery with optimized parp inhibition in advanced solid tumors: a phase i trial of gapped scheduling
Source: Nat Commun. 2025 Oct 27;16:9457. doi: 10.1038/s41467-025-64509-5 (PMC12559310; doi:10.1038/s41467-025-64509-5)
Supplement: Supplementary file 1 — Supplementary Information [file 41467_2025_64509_MOESM1_ESM.pdf]

Supplementary Information of Tumor-Targeted TOP1 Inhibitor Delivery with Optimized PARP Inhibition in Advanced Solid Tumors: A Phase I Trial of Gapped Scheduling

Supplementary Note 1

Supplemental Tables/ Figures

Patient ID	Prior Treatment	TOP1	PARP#
1	Cisplatin, Carboplatin and Pemetrexed, clinical trials including PI3 kinase/mTOR inhibitor, Docetaxel, Pembrolizumab	None	None
2	Carboplatin/Pemetrexed, Nivolumab	None	None
3	Carboplatin/Paclitaxel, Carboplatin/Doxil, Abraxane/Bevacizumab, Gemcitabine/Docetaxel, and Durvalumab/Cediranib.	None	None
4	Cisplatin, Taxane, and Bevacizumab , Nivolumab	None	None
5	Carboplatin/Taxol, Paclitaxel, Doxil/Carboplatin, Durvalumab/Olaparib	None	Yes
6	Gemcitabine/Oxaliplatin ,Gemcitabine/Capecitabine	None	None
7	Carboplatin/Etoposide, Topotecan,Nivolumab	Yes	None
8	Cisplatin/Etoposide, Topotecan	Yes	None
9	FOLFOX, FOLFIRI, PDL-1 / TGFB Receptor Trap	Yes	None
10	Carboplatin/Etoposide	None	None
11	Cisplatin, Topotecan, paclitaxel and bevacizumab,PDL-1 / TGFB Receptor Trap	Yes	None
12	FOLFIRINOX, Durvalumab, Pentostatin/Cyclophosphamide/Anti-Mesothelin Recombinant Immunotoxin SS1P, Gemcitabine/Nab-Paclitaxel	Yes	None
13	Gemcitabine/Capecitabine, FOLFILINOX, Gemcitabine /Nab-Paclitaxel, Tremelimumab	Yes	None
14	Carboplatin/Pemetrexed, Nivolumab, Gemcitabine/ Nab-Paclitaxel	None	None
15	Pembrolizumab, Carboplatin/Paclitaxel	None	None
16	Taxol/Carboplatin, Gemcitabine, Cisplatin/Bevacizumab	None	None
17	Pemetrexed/Cisplatin, Gemcitabine/Cisplatin, Pembrolizumab/Pemetrexed/Carboplatin/Bevacizumab, Pembrolizumab/Pemetrexed/Bevacizumab.	None	None
18	Pemetrexed/Cisplatin, Nivolumab	None	None
19	FOLFIRINOX, Gemcitabine/Capecitabine, Gemcitabine/Nab-Paclitaxel, Avelumab/ NHS-IL12	Yes	None
20	Gemcitabine/Cisplatin, Capecitabine, Avelumab/ NHS-IL12	None	None
21	Cabozantanib, PDL1/TGF B trap	None	None
22	Gemcitabine/Oxaliplatin, Capecitabine	None	None
23	Carboplatin/Paclitaxel, Sunitinib	None	None
24	FOLFOX/Bevacizumab, FOLFIRI / Bevacizumab, Durvalumab/Cediranib	Yes	None
	Total	33%	4%

Table S1: Summary of prior treatments received for each by patient

Cycle	N	C_{MAX} (ng/mL)			AUC_{INF} (hr*ng/mL)			CL (L/hr)	V_{SS} (L)	$T_{1/2}$ (hr)
		Con+Un	Con	Un	Con+Un	Con	Un	Con+Un	Con+Un	Con+Un
1	24	5,406 ± 966.9	5,230 ± 934.9	266.9 ± 120.1	◊194,928 ± 53,760.7	◊168,737 ± 49063.1	⊥25,418.6 ± 10,627.3	◊0.117 ± 0.030	◊6.40 ± 1.66	◊47.42 ± 18.22
6	5	5,115 ± 965.0	4,989 ± 933.0	180.6 ± 85.03	173,181 ± 33,164.4	158,014 ± 29,146.4	◊22,294.5 ± 13,773.5	0.0944 ± 0.020	4.36 ± 1.62	37.17 ± 14.04

Table S2: Pharmacokinetic Parameters for CRLX101

*All values are reported as arithmetic mean ± SD.
Abbreviations: C_{MAX}: maximum plasma concentration; AUC_{INF}: area under the concentration-time curve from 0 to infinity; CL: total systemic clearance; V_{SS}: volume of distribution at steady-state; Con+Un: conjugated+unconjugated CPT, representing "total" CPT present; Con: Conjugated or polymer-conjugated CPT encapsulated in the CRLX101 formulation (obtained via subtracting freely circulating CPT from "total" CPT); Un: unconjugated, or freely circulating CPT after release from CRLX101.

⊥ 4 patients were excluded from this calculation due to low confidence in elimination rate calculation (r^2<0.7).

◊ One patient was excluded from this calculation due to low confidence in elimination rate calculation (r^2<0.7).

Dose (mg)	N	C_{MAX} (ng/mL)
100	2	2843.1 ± 947.62
150	5	2359.8 ± 1548.0
200	3	4496.7 ± 2375.2
250	7	6338.6 ± 4865.5
300	6	5594.6 ± 4132.4

Table S3: C_{MAX} for Olaparib
*All values are reported as arithmetic mean ± SD. C_{MAX}: maximum plasma concentration.

Combination		MTD	Topo1i<br><br>% of MTD	PARPi<br><br>% of MTD	DLT	Ref
CRLX101	*Olaparib*	*CRLX101 12 mg/m2 d1,15;q3w*<br><br>*Olaparib 250 tab bid d3-13, 17-26*	*80%*	*≈83%*	*Myelosuppression*	*Current study*
Irinotecan	Olaparib	Irinotecan 200 mg/m2; q3w<br><br>Olaparib 50 mg qd d1-21	57%	6%	Diarrhea, myelosuppression	Chen, Inv New Drugs, 2016
		Irinotecan 125 mg/m2 q2w<br><br>Olaparib 50 mg bid d1-5	≈69%	≈12%	Anorexia/fatigue	
Irinotecan	Veliparib	Irinotecan 100 mg/m2 q1,8; q3w<br><br>Veliparib 40 bid d1-14	≈80	≈10%	Diarrhea, fatigue, myelosuppression	LoRusso, Clin Can Res, 2016
Liposomal Irinotecan	Veliparib	Veliparib dosage of 50 mg BID d5-12 and d19-25 Q3w<br><br>Nal- Irinotecan 70 mg/m2<br><br>q2w	100%	13%	Diarrhea, Hyponatremia	LaRose, Oncologist, 2023
Topotecan	Olaparib	Topotecan 1 mg/m2/d1-3; q3w<br><br>Olaparib 100 mg bid d1-21	≈40%	25%	Myelosuppression	Samol, Inv New Drugs, 2012
Topotecan	Veliparib	Topotecan 0.6 mg/m2/d1-5; q3w<br><br>Veliparib 10 bid d1-5	40%	≈3%	Myelosuppression	Kummar, Can Res, 2011
Topotecan	Veliparib	Tpotecan 3 mg/m2 d2,9,16; q4w<br><br>Veliparib 300 mg bid d1-3, 8-10,15-17	≈75%	≈75%	Myelosuppression	Hendrickson, Clin Can Res, 2017

Table S4: Dose levels of Topoisomerase inhibitors and PARP inhibitors achieved in previous clinical trials

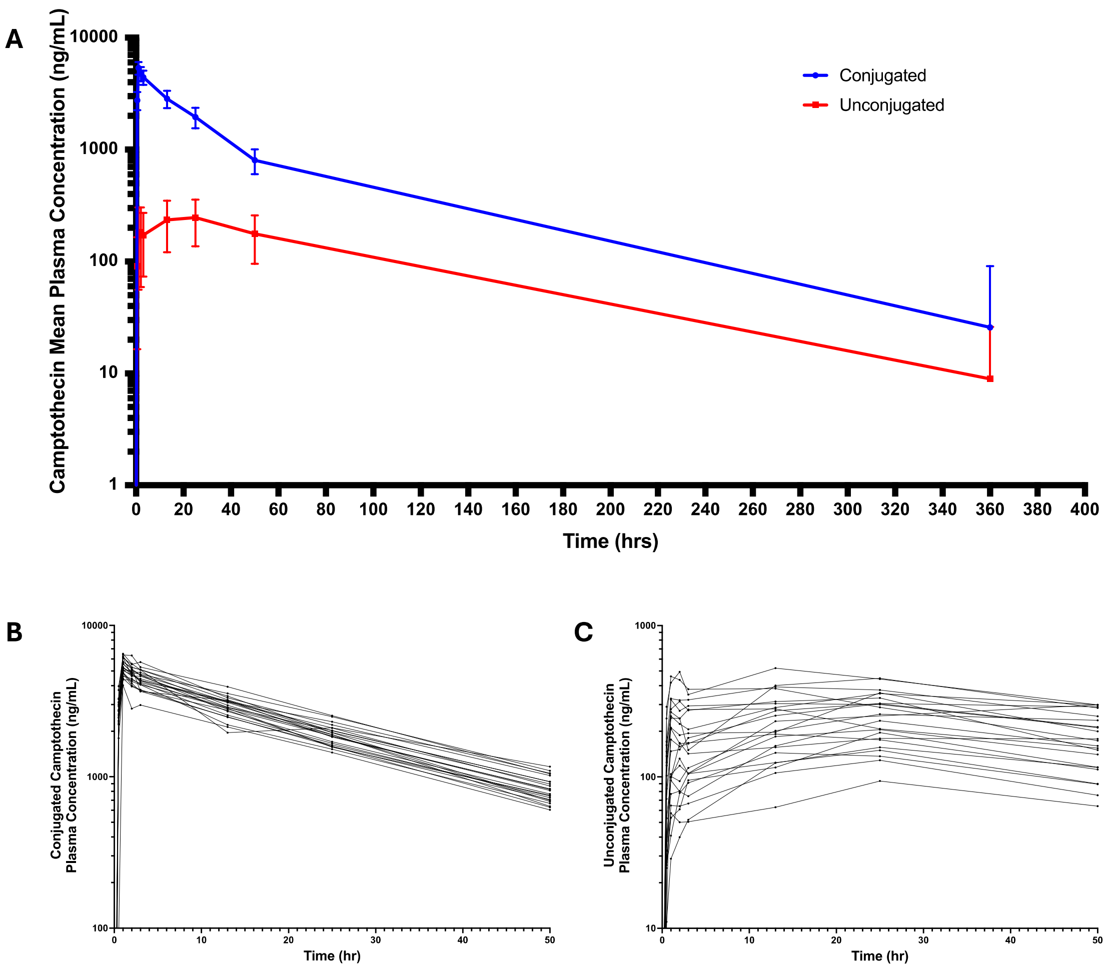

Figure S1: **Plasma concentration–time profiles of polymer-conjugated and unconjugated camptothecin following first-dose CRLX101 administration.** Mean ± SD plasma concentration versus time for both polymer-conjugated camptothecin (CPT) and polymer-unconjugated CPT following administration of the first dose of CRLX101 during cycle 1 (**A**). Plasma concentrations from each patient(N=24 patients) versus time for polymer-conjugated CPT (**B**) and polymer-

unconjugated CPT (**C**) following administration of the first dose of CRLX101 during cycle 1 (N=23 patients; One patient had an incomplete serial plasma sample collection, thus was omitted)

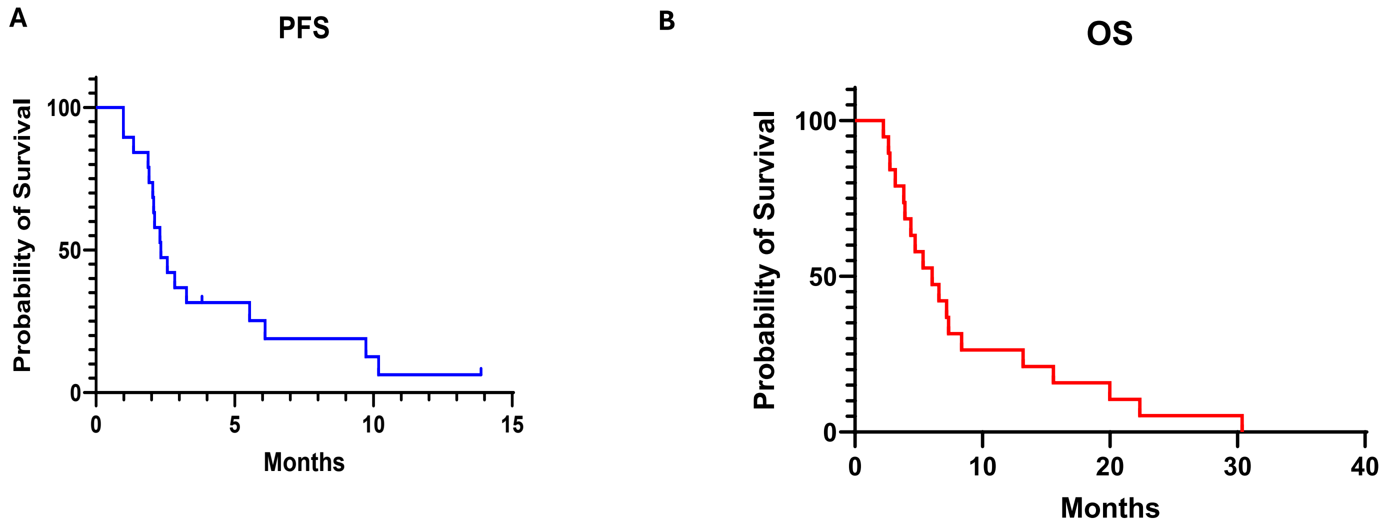

Figure S2: Survival data: (A) Progression Free survival and (B) Overall survival in the evaluable cohort (N=19 Patients).

A

Figure S3: Association of DDR gene alterations and HRD scores with clinical benefit across multiple tumor types (A) Association between somatic variants in DDR genes and clinical benefit (n=11 patient tumors). PAAD: pancreatic adenocarcinoma, CHOL: cholangiocarcinoma; SCLC: small cell lung cancer; MESO: mesothelioma; NSCLC: non-small cell lung cancer; OV: ovarian/fallopian tube cancer; COAD: colorectal carcinoma; CESC: cervical carcinoma. **(B) HRD score and clinical benefit among 6 patients with matched tumor-normal exome sequencing; No statistical comparison was done and the plot represents the distribution of HRD scores across samples.**

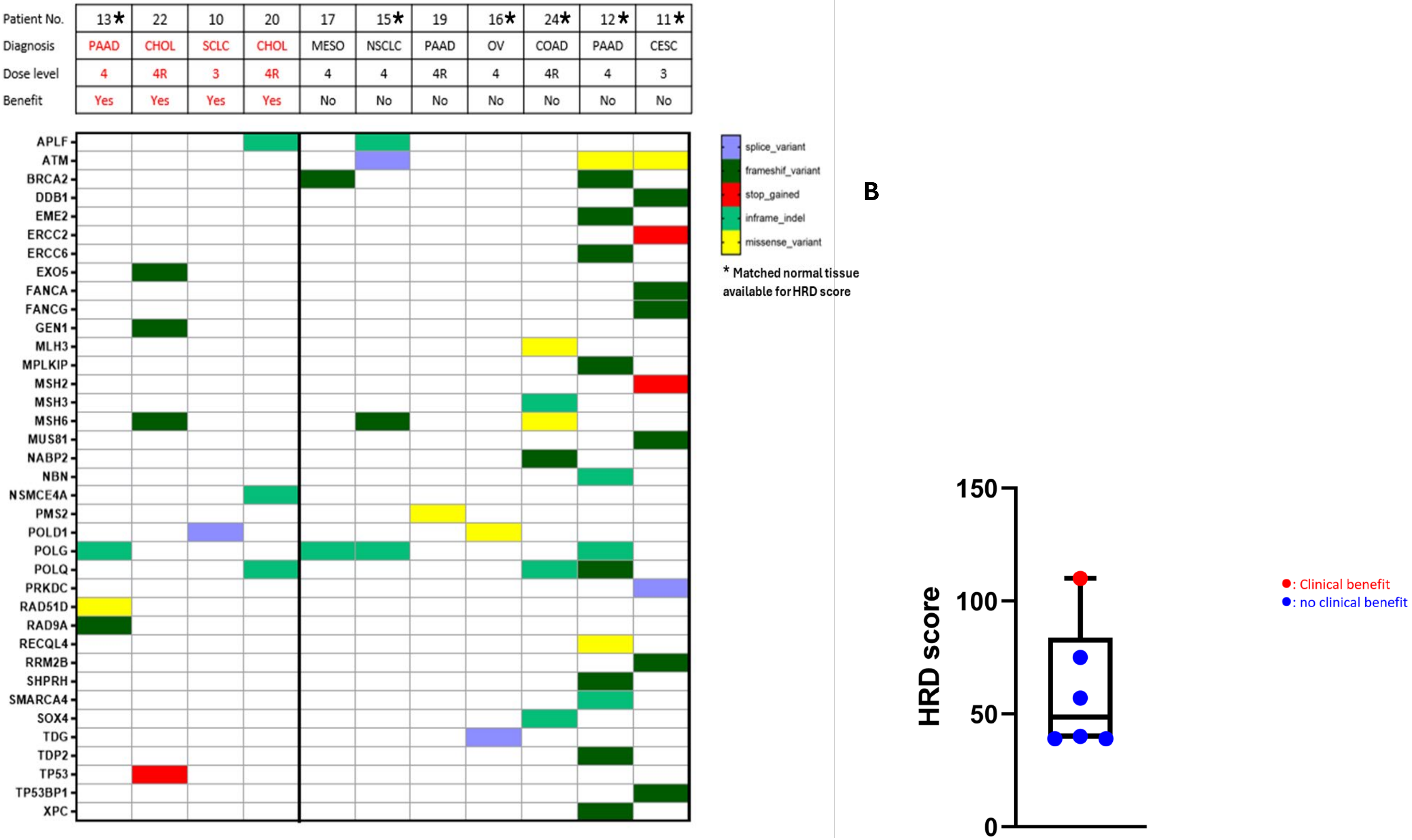

Supplementary Note 2: Clinical Trial Protocol

Abbreviated Title: CRLX101 in lung cancer
NCI Protocol #:
Version Date: April 13, 2016

Title: A Phase I/II Trial of CRLX101, a Nanoparticle Camptothecin with Olaparib in Patients with Relapsed/Refractory Small Cell Lung Cancer

Principal Investigator: Anish Thomas, MBBS, M.D.
Thoracic and Gastrointestinal Oncology Branch (TGIB)
Center for Cancer Research (CCR)
National Cancer Institute (NCI)
10 Center Drive, Room 4-5330
Bethesda, MD 20892
TEL: 301-451-8418
FAX: 954-827-0184
E-mail: anish.thomas@nih.gov

Associate Investigators:

Yves Pommier, M.D., Ph.D.<br>Developmental Therapeutics Branch (DTB)<br>CCR/NCI<br>National Cancer Institute<br>37 Convent Drive Room 5068<br>Bethesda, MD 20892<br>TEL: 301-496-5944<br>FAX: 301-402-0752<br>E-mail: pommier@nih.gov	Jane Trepel<br>DTB/CCR/NCI<br>10 Center Drive<br>Room 12N230<br>Bethesda, MD 20892<br>TEL: 301-496-1547<br>E-mail: jt66h@nih.gov
Christophe Redon, Ph.D.<br>DTB/CCR/NCI<br>37 Convent Drive Room 5056<br>Bethesda, MD 20892<br>TEL: 301-451-8576<br>FAX: 301-402-0752<br>E-mail: redonc@mail.nih.gov	Raffit Hassan, M.D.<br>TGIB/CCR/NCI<br>37 Convent Drive Room 5116<br>Bethesda, MD 20892<br>TEL: 301-451-8742<br>FAX: 301-402-9469<br>E-mail: rh276q@nih.gov
Arun Rajan, M.D.<br>TGIB/CCR/NCI<br>10 Center Drive Room 12N226<br>Bethesda, MD 20892<br>TEL: 301-594-5322<br>E-mail: rajana@mail.nih.gov	Eva Szabo, M.D.<br>Division of Cancer Prevention/NCI<br>9609 Medical Center Drive Room 5E102<br>Rockville, MD 20850<br>TEL: 240-276-7011<br>E-mail: szaboe@mail.nih.gov
Christine Alewine, M.D., Ph.D.<br>Laboratory of Molecular Biology/CCR/NCI<br>National Cancer Institute<br>37 Convent Drive Room 5116B	Udayan Guha, M.D., Ph.D.<br>TGIB/CCR/NCI<br>10 Center Drive Room 13N240-C<br>Bethesda, MD 20892

Bethesda, MD 20892<br>TEL: 301-451-8725<br>E-mail: alewinecc@mail.nih.gov	TEL: 301-402-3524<br>FAX: 301-402-0172<br>E-mail: udayan.guha@nih.gov
Emerson Padiernos, N.P.<br>TGIB/CCR/NCI<br>10 Center Drive Room 13N230<br>Bethesda, MD 20892<br>TEL: 301-402-9741<br>E-mail: emerson.padiernos@nih.gov	Linda Sciuto, R.N.<br>Office of the Clinical Director (OCD)/CCR/NCI<br>10 Center Drive Room 13N230<br>Bethesda, MD 20892<br>TEL: 301-451-9707<br>E-mail: lsciuto@mail.nih.gov
Susan Perry, R.N.<br>OCD/CCR/NCI<br>10 Center Drive Room 13N230<br>Bethesda, MD 20892<br>TEL: 301-402-4423<br>E-mail: perrys@mail.nih.gov	Betsy Morrow<br>TGIB/CCR/NCI<br>10 Center Drive Room 3B51<br>Bethesda, MD 20892<br>TEL: 301-402-5688<br>E-mail: morrowbj@mail.nih.gov
Lisa Bengtson, R.N.<br>OCD/CCR/NCI<br>10 Center Drive Room 13N230<br>Bethesda, MD 20892<br>TEL: 301-435-5398<br>E-mail: bengtsonla@mail.nih.gov	Yvonne Mallory, R.N.<br>OCD/CCR/NCI<br>10 Center Drive Room 12N214<br>Bethesda, MD 20892<br>TEL: 301-402-0255<br>FAX: 301-480-2462<br>E-mail: malloryy@mail.nih.gov
Seth Steinberg, Ph.D.<br>Biostatistics and Data Management/OCD/NCI<br>BLDG 9609 RM 2W334<br>Rockville MD 20850<br>TEL: 240-276-556<br>FAX: 240-276-7885<br>E-mail: steinbes@mail.nih.gov	William D. Figg, PharmD, MBA<br>Genitourinary Malignancy Branch<br>CCR/NCI/NIH<br>10 Center Drive, Room 5A01<br>Bethesda, MD 20892<br>TEL: 301-402-3622<br>FAX: 301-402-8606<br>Email: wf13e@nih.gov
Cody J. Peer, PhD<br>Clinical Pharmacology Program<br>OCD/CCR/NCI/NIH<br>10 Center Drive, Room 5A10<br>Bethesda, MD 20892<br>TEL: 301-451-4982<br>FAX: 301-402-8606<br>Email: cody.peer@nih.gov	Chul Kim, MD<br>OCD/CCR/NCI<br>BG 10 RM 12N226 MSC 1906<br>10 Center Drive<br>Bethesda, MD 20892-1906<br>TEL: 301-496-4916<br>Email: kimc5@mail.nih.gov

Referral Contact

Linda Sciuto, R.N.
Office of the Clinical Director (OCD)
National Cancer Institute
10 Center Drive

Room 13N230

Study Coordinator

Linda Sciuto, R.N.
Office of the Clinical Director (OCD)
National Cancer Institute
10 Center Drive
Room 13N230
TEL: 301-451-9707
E-mail: lsciuto@mail.nih.gov

Investigational Agents:

Drug Name:	CRLX101	Olaparib
IND Number:	Pending	Pending
Sponsor:	Center for Cancer Research	Center for Cancer Research
Manufacturer:	Cerulean Pharma, Inc.	AstraZeneca

PRÉCIS

Background:

- Small cell lung cancer (SCLC) is an aggressive cancer with a poor prognosis.
- Although highly responsive to chemotherapy initially, SCLC relapses quickly and becomes refractory to treatment within a few months.
- The use of PARP inhibitors in combination with chemotherapy builds upon pre-clinical data in lung cancer and other cancers supporting the notion that PARP inhibitors potentiate the effect of DNA damaging therapies.
- Despite their highly synergistic activity in preclinical models, human studies combining PARP inhibitors and camptothecins have not translated into clinical benefit due to enhanced toxicity with the combination.
- One approach to improve ability to combine camptothecins with agents that sensitize their activity like PARP inhibitors is to use alternative formulations that minimize toxicity to the normal tissues.
- CRLX101 is a nanoparticle drug conjugate composed of 20 (S)-camptothecin (a potent and highly selective topoisomerase I inhibitor) conjugated to a linear, cyclodextrin-polyethylene glycol-based polymer.
- Olaparib is a PARP inhibitor indicated as monotherapy in patients with deleterious or suspected deleterious germline BRCA mutated advanced ovarian cancer who have been treated with three or more prior lines of chemotherapy. Olaparib has an established safety profile and it is under investigation in a number of different cancers.

Objectives:

- Phase I: To determine the MTD/ recommended Phase 2 dose (RP2D) of CRLX101 in combination with olaparib in patients with refractory cancers
- Phase II: To determine the antitumor activity of olaparib plus CRLX101 with respect to progression free survival at 16 weeks separately in SCLC patients with resistant and sensitive relapse

Eligibility:

Phase I

- Male or female adult patients $\geq$18 years of age
- Histologically or cytologically confirmed, advanced solid tumor that is refractory to standard therapy and/or for whom no further standard therapy is available
- ECOG Performance Status of 0, 1 or 2

Phase II

- Male or female patients $\geq$ 18 years old
- Have a pathologically (histology or cytology) confirmed diagnosis of SCLC

- Disease progression on or after at least one platinum-based standard chemotherapy regimen for either limited or extensive stage disease.
- Have measurable disease per RECIST 1.1
- ECOG performance status of 0, 1 or 2

Design:

- Patients meeting eligibility criteria will receive CRLX101 (IV Q 2weeks) plus olaparib (PO BID days 3-13 and days 17-26; On days 13 and 26, only one dose of olaparib will be administered in the morning) administered in 28 day cycles, until disease progression or development of intolerable side effects. The MTD of the combination will be used in Phase II.
- Blood, tumor and hair samples will be collected at multiple time points for PK, PD analyses. Tumor biopsies are optional and will be performed only in SCLC patients (in Phase I and II) at the following time points: pre-treatment, on cycle 1 day 4 and at disease progression.
- Toxicity will be graded according to CTCAE version 4.0.
- Tumor assessments will be made using CT scans (chest, abdomen and pelvis) at baseline and after every 2 cycles according to RECIST version 1.1.
- After discontinuation of study treatment, follow-up for survival will be carried out every 3 months.
- The maximum number of patients on the phase I portion of the trial is 30 and the two cohorts in phase II may accrue up to 20 evaluable patients each. Thus, the maximum number of evaluable patients who may enroll on this trial is 70. In order to allow for a small number of in-evaluable patients, the accrual ceiling will be set at 75.
- It is anticipated that approximately 1 to 2 patients per month may enroll onto this trial; the trial is expect to complete accrual in 3-4 years.

Trial schema and dose escalation

Dose level	CRLX101, mg/m2 (IV q 2 weeks D1 and D15)	Olaparib tablet, mg (PO BID Days 3-13* and 17-26*)
1	12	100
2	12	150
3	12	200
4	12	250
5	15	250
28 day cycles; biopsies pre-treatment and on day 4 (24 hours post- olaparib) and at disease progression; * On days 13 and 26, only one dose of olaparib will be administered in the morning.		

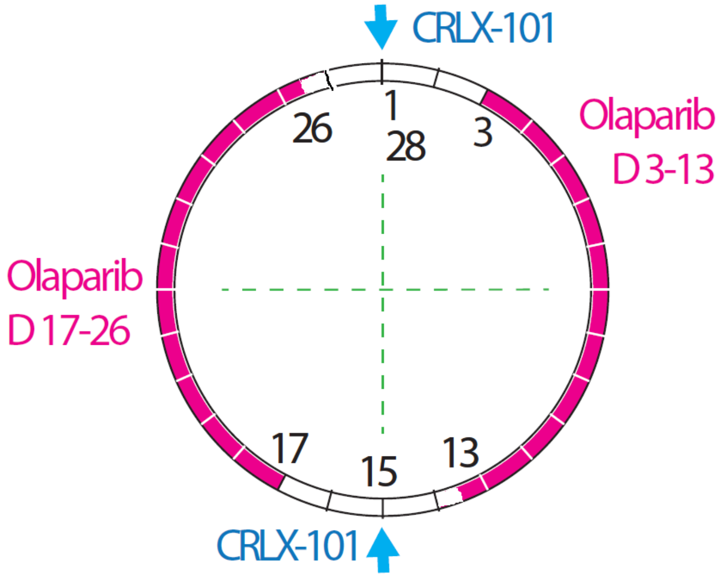

TABLE OF CONTENTS

PRÉCIS 4
TABLE OF CONTENTS 7
1. INTRODUCTION 13
1.1 Study Objectives 13
1.1.1 Primary Objectives 13
1.1.2 Secondary Objectives 13
1.1.3 Exploratory Objectives 13
1.2 Background and Rationale 13
1.2.1 Small Cell Lung Cancer 13
1.2.2 PARP as a Therapeutic Target in Small Cell Lung Cancer 14
1.2.3 PARP Inhibitors and DNA Damaging Chemotherapy are Synergistic 15
1.2.4 Safely combining PARP Inhibitors with Camptothecins 16
1.2.5 CRLX101 Preclinical Toxicology 17
1.2.6 CRLX101 phase I clinical trial 17
1.2.7 Adverse Events with CRLX101 Monotherapy 18
1.2.8 Adverse Events Associated with CRLX101 Combination Therapy 18
1.2.9 CRLX101 Clinical Development Overview 20
1.2.10 Olaparib 20
1.2.11 Preclinical Data of CRLX101 plus Olaparib 21
1.2.12 Hypothesis 22
1.2.13 Rationale 23
2 ELIGIBILITY ASSESSMENT AND ENROLLMENT 24
2.1 Eligibility Criteria 24
2.1.1 Inclusion Criteria - Phase I 24
2.1.2 Inclusion Criteria - Phase II 26
2.1.3 Exclusion Criteria - Phase I and II 28
2.2 Screening Evaluation 29
2.2.1 History and physical exam (including height, weight, vital signs and performance status) 29
2.2.2 Blood tests (for organ function) 29
2.2.3 Viral Markers Protocol Screen (HBsAg, anti-HCV, anti-HIV) within 3 months of C1D1. 29

2.2.4 Confirmation of diagnosis by the NCI Laboratory of Pathology 29
2.2.5 CT chest/abdomen/ pelvis 29
2.2.6 Electrocardiogram 29
2.2.7 Echocardiogram 29
2.2.8 Urine or serum HCG for women of child-bearing potential (to be performed within 3 days of study enrollment) 29
2.3 Registration Procedures 29
2.4 Stratification Procedures 30
2.5 Baseline Evaluation 30
3 STUDY IMPLEMENTATION 30
3.1 Study Design 30
3.1.1 Dose Limiting Toxicity 31
3.1.2 Dose Escalation 31
3.2 Drug Administration 32
3.2.1 CRLX101 32
3.2.2 Olaparib 33
3.3 Dose Modifications 34
3.3.1 General Recommendation for Dose Modification 34
3.3.2 Dosing Delays/Dose Modifications and Management of Toxicities 35
3.3.3 General Recommendations for Dose Modification and Management of Non-Hematologic Adverse Events 37
3.3.4 Management of the hypersensitivity/infusion reactions 38
3.3.5 **Laboratory safety assessment** 39
3.4 Study Calendar 39
3.5 Criteria for Removal from Protocol Therapy and Off Study Criteria 41
3.5.1 Criteria for Removal from Protocol Therapy 41
3.5.2 Off-Study Criteria 41
3.5.3 Off-Study Procedure 42
4 CONCOMITANT MEDICATIONS/MEASURES 42
4.1 Supportive Care 43
4.1.1 Other Concomitant Medications 44
4.1.2 Palliative radiotherapy 44
4.1.3 Administration of other anti-cancer agents 44

4.1.4 Medications that may NOT be administered 44
4.1.5 Overdose 44
4.1.6 Maternal exposure 45
4.1.7 Paternal exposure 45
4.1.8 Pregnancy 45
5 BIOSPECIMEN COLLECTION 45
5.1 Correlative Studies for Research/Pharmacokinetic Studies 45
5.1.1 γ-H2AX 49
5.1.2 SLFN11 50
5.1.3 Tumor biopsies and genomic DNA 50
5.1.4 Immune Subsets 51
5.1.5 Circulating Tumor Cells 51
5.1.6 A multiplexed gene expression approach to profiling immune gene and DNA damage response signatures 51
5.1.7 PK 52
5.2 Sample Storage, Tracking and Disposition 52
5.2.1 Trepel Lab 53
5.2.2 Clinical Pharmacology Program (Figg Lab) 54
5.2.3 Redon (Aladjem) Lab 54
5.2.4 Future Use/IRB Reporting/Protocol Completion/Sample Destruction 55
5.3 Samples for Genetic/Genomic Analysis 55
5.3.1 Description of the scope of genetic/genomic analysis 55
5.3.2 Certificate of Confidentiality 55
5.3.3 Management of Results 56
6 DATA COLLECTION AND EVALUATION 56
6.1 Data Collection 56
6.2 Data Sharing Plans 57
6.2.1 Human Data Sharing Plan 57
6.2.2 Genomic Data Sharing Plan 57
6.3 Response Criteria 57
6.3.1 Definitions 58
6.3.2 Disease Parameters 58

6.3.3 Methods for Evaluation of Measurable Disease 59
6.3.4 Response Criteria 61
6.3.5 Duration of Response 63
6.3.6 Progression-Free Survival 63
6.3.7 Response Review 64
6.4 Toxicity Criteria 64
7 SAFETY REPORTING REQUIREMENTS/DATA AND SAFETY MONITORING PLAN 64
7.1 Definitions 64
7.1.1 Adverse Event 64
7.1.2 Suspected adverse reaction 64
7.1.3 Unexpected adverse reaction 65
7.1.4 Serious 65
7.1.5 Serious Adverse Event 65
7.1.6 Disability 65
7.1.7 Life-threatening adverse drug experience 65
7.1.8 Protocol Deviation (NIH Definition) 65
7.1.9 Non-compliance (NIH Definition) 66
7.1.10 Unanticipated Problem 66
7.2 NCI-IRB Reporting 66
7.2.1 NCI-IRB Expedited Reporting of Unanticipated Problems and Deaths 66
7.2.2 NCI-IRB Requirements for PI Reporting at Continuing Review 66
7.2.3 NCI-IRB Reporting of IND Safety Reports 67
7.3 IND Sponsor Reporting Criteria 67
7.4 FDA Reporting Criteria 67
7.4.1 IND Safety Reports to the FDA (Refer to 21 CFR 312.32) 67
7.4.2 FDA Annual Reports (Refer to 21 CFR 312.33) 68
7.4.3 Expedited Adverse Event Reporting Criteria to the IND Manufacturer 68
7.5 Data and Safety Monitoring Plan 69
7.5.1 Principal Investigator/Research Team 69
7.5.2 Sponsor Monitoring Plan 70
8 STATISTICAL CONSIDERATIONS 70

9 COLLABORATIVE AGREEMENTS 72
9.1 Cooperative Research and Development Agreement (CRADA) 72
9.1.1 Cereulean Pharmaceuticals 72
9.1.2 Astra Zeneca 72
10 HUMAN SUBJECTS PROTECTIONS 72
10.1 Rationale For Subject Selection 72
10.2 Participation of Children 72
10.3 Evaluation of Benefits and Risks/Discomforts 72
10.3.1 Risks 72
10.3.2 Benefits 73
10.4 Consent and Assent Process and Documentation 73
10.4.1 Informed consent of non-English speaking subjects 73
11 PHARMACEUTICAL INFORMATION 74
11.1 CRLX101-212 (IND#) 74
11.1.1 Description 74
11.1.2 Supplier/How Supplied 74
11.1.3 Handling and Dispensing 74
11.1.4 Preparation 74
11.1.5 Storage and Stability 75
11.1.6 Dosage and Administration 75
11.1.7 Premedication and Hydration 76
11.1.8 Return and Retention of CRLX101-212 76
11.2 Olaparib (AZD2281; Lynparza) 76
11.2.1 Source 76
11.2.2 Pre-clinical experience 76
11.2.3 Toxicology and safety pharmacology summary 76
11.2.4 Approved indications and clinical experience; ; capsule formulation 77
11.2.5 Indications and Usage 77
11.2.6 Dosage and Administration 77
11.2.7 Tablet Dosage Forms and Strengths 77
11.2.8 Warnings and Precautions 77
11.2.9 Adverse Reactions 77

11.2.10 Drug Interactions 77
12 REFERENCES 79
13 APPENDICES 82
13.1 Appendix A: Performance Status Criteria 82
13.2 Appendix B: List of drugs that may have potential CYP3A4 interactions 83
13.2.1 CYP3A4 Substrates 83
13.2.2 CYP3A4 Inhibitors 84
13.2.3 CYP3A4 Inducers 84
13.3 Appendix C: Patient's Pill Diary: Olaparib 86

1. INTRODUCTION

1.1 STUDY OBJECTIVES

1.1.1 Primary Objectives

1.1.1.1 Phase I: To determine the MTD/ recommended Phase 2 dose (RP2D) of CRLX101 plus olaparib in patients with refractory cancers.

1.1.1.2 Phase II: To determine the antitumor activity of olaparib plus CRLX101 with respect to progression free survival 16 weeks separately in SCLC patients with resistant and sensitive relapse

1.1.2 Secondary Objectives

1.1.2.1 To evaluate the pharmacokinetic profile of CRLX101 (both the total drug and released camptothecin) and olaparib in plasma.

1.1.2.2 To evaluate the pharmacodynamic (PD) activity of CRLX101 in blood, surrogate tissue and tumor biopsy specimens.

1.1.2.3 To determine the duration of response (DOR), overall survival (OS), and progression-free survival (PFS) of the combination.

1.1.2.4 To further explore safety for combination.

1.1.3 Exploratory Objectives

1.1.3.1 To explore possible correlations between clinical response and biomarkers

1.2 BACKGROUND AND RATIONALE

1.2.1 Small Cell Lung Cancer

Small cell lung cancer (SCLC) is an aggressive cancer with a poor prognosis. Annually there are approximately 34,000 new cases in the United States alone. SCLC is characterized by rapid doubling time, high growth fraction and early and widespread metastatic involvement. Approximately two thirds of patients present with extensive-stage disease with tumor involvement of contralateral lung, liver, adrenal glands, brain, bones and/or bone marrow. The median survival of patients with extensive-stage SCLC (ES-SCLC) ranges from 8 to 13 months. Less than 5% of patients survive two years and less than 2% of patients survive five years after diagnosis.

Standard therapy for patients with ES-SCLC consists of platinum and etoposide followed by prophylactic cranial irradiation in patients with a response [2]. Although highly responsive to chemotherapy initially, SCLC relapses quickly and becomes refractory to treatment within a few months. There is only one FDA approved treatment for patients with relapsed SCLC after first-line chemotherapy: topotecan, a camptothecin which inhibits relegation of topoisomerase I-mediated single-strand DNA breaks leading to lethal double-strand DNA breaks. In patients with disease that is refractory to or relapsed after first-line chemotherapy, the median survival ranges from 2 to 6 months.

Patients with disease progression during or after their initial treatment are classified into categories based on the treatment free intervals from the initial chemotherapy. Sensitive relapse is tumor progression that occurs 90 days or more after the last day of initial chemotherapy. Resistant relapse is tumor progression that occurs within 90 days of the last day of initial chemotherapy. Refractory relapse is when tumor progresses during the initial therapy or did not respond to initial therapy. These parameters have been found to be independent predictors of probability of responding to second-line chemotherapy [3].

The standard treatment of ES-SCLC today reflects the prevailing state-of-the-art from the early 1980s. Among the many strategies that have been evaluated unsuccessfully over the last three decades are dose-dense chemotherapy regimens, addition of a third drug to standard two drug chemotherapies, alternating non-cross resistant chemotherapy regimens, maintenance therapy and more recently targeted therapies. Not unexpectedly, the outcomes for these patients have not significantly changed over this time. Clearly there is a critical need for newer therapeutic approaches for patients with SCLC.

1.2.2 PARP as a Therapeutic Target in Small Cell Lung Cancer

Poly-(ADP)-ribose polymerases (PARPs) are a large family comprising of proteins that are critical regulatory components in DNA damage repair, maintenance of genomic integrity and other cellular processes [4]. PARP-1 and PARP-2 are activated by single-strand DNA breaks (SSB). When SSBs are formed, PARP binds at the end of broken DNA strands, activating its enzymatic activity. Activated PARP catalyzes addition of long polymers of ADP-ribose (PAR). This lengthening PAR chain (PARylation) builds up a large negatively charged structure at the SSB which recruits other DNA repair enzymes that collectively repair DNA breaks via base excision repair (BER). PARP inhibition by inhibiting BER leads to the conversion of SSB to DNA double-strand breaks (DSB). The activity of PARP inhibitors are best established in cancers with mutations in BRCA1/2 and other DNA repair genes that result in synthetic lethality in the setting of PARP inhibition (which provides a second "hit" to the DNA repair machinery). Olaparib monotherapy was recently FDA-approved for patients with advanced, BRCA-mutated ovarian cancer who have received three or more lines of chemotherapy.

Drugs that target DNA damage response (DDR), including PARP inhibitors, have shown promising activity against SCLC in pre-clinical models and in early clinical trials. Proteomic profiling of a large panel of SCLC cell lines led to the observation that PARP1, Chk1, and several other DNA repair proteins are expressed at high levels in SCLC [5]. These studies also confirmed PARP1 overexpression in patient tumors at the protein level by immunohistochemistry and at the mRNA level. Based on this finding, several PARP inhibitors were tested in pre-clinical models of SCLC. Olaparib, rucaparib, and talazoparib (previously BMN-673) all demonstrated striking single agent activity in a majority of SCLC cell lines tested. Furthermore, the addition of a PARP inhibitor to standard chemotherapies (e.g., cisplatin, etoposide and/or topotecan) and radiation further potentiated their effect [6]. In animal models including xenografts and patient-derived xenografts (PDXs), talazoparib has demonstrated significant anti-tumor activity as a single agent, comparable or superior to cisplatin [7].

Following these observations, several clinical trials were initiated to investigate the effects of PARP inhibition in SCLC patients. The first two studies to complete enrollment investigated the use of PARP inhibitors in relapsed SCLC. In the first study, single-agent talazoparib (BMN-673)

was tested in an expansion cohort of patients with platinum-sensitive SCLC relapse (NCT01286987). Preliminary data from this trial demonstrated 2/23 patients with RECIST confirmed partial responses and 3/23 with stable disease lasting more than 24 weeks (clinical benefit rate of 25%). More than half of patients treated had some tumor volume reduction as their best response [8]. In the second study, the oral alkylating drug temozolomide with or without veliparib (ABT-888) was studied in 100 patients with sensitive or refractory relapse (NCT01638546). This trial recently completed enrollment and analysis of the results are ongoing.

1.2.3 PARP Inhibitors and DNA Damaging Chemotherapy are Synergistic

The use of PARP inhibitors in combination with chemotherapy builds upon prior pre-clinical data in lung cancer and other malignancies supporting the notion that PARP inhibitors potentiate the effect of other DNA damaging therapies. Supporting this hypothesis, PARP inhibition has been reported to down regulate key components of the DNA repair machinery and enhanced the efficacy of chemotherapy in preclinical SCLC models [5]. PARP inhibition sensitizes cancer cells both to cytotoxic chemotherapy, such as alkylators (temozolomide, cyclophosphamide) or camptothecins (irinotecan, topotecan) and to ionizing radiation- all of which induce DNA damage requiring BER [9]. PARP inhibitors are highly effective in combination with camptothecins in tumors with and without defects in homologous recombination [1, 10-12]. The Pommier laboratory has demonstrated highly synergistic activity of olaparib in combination with camptothecins (**Figure 1**) [1]. They observed that the synergistic activity is due to its catalytic PARP inhibitory activity rather than due to trapping of PARP-DNA complexes.

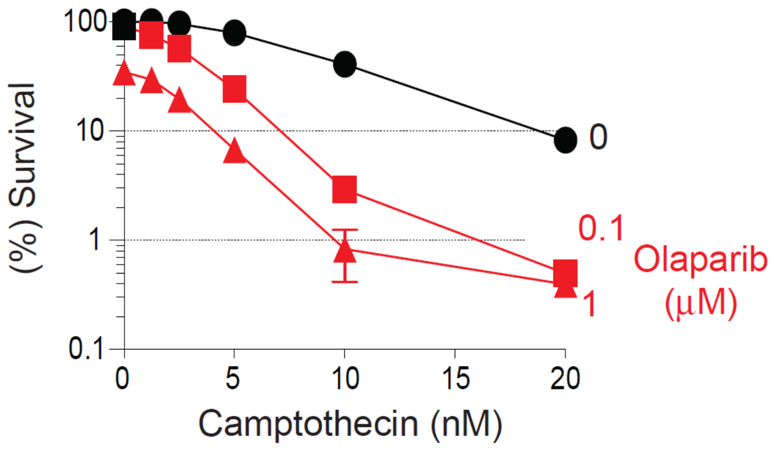

Figure 1: Synergistic activity of camptothecins and olaparib in DT40 cells with a synergistic combination index (<0.3), even when the doses of individual agents are low [1].

Despite their highly synergistic activity in preclinical models, human studies combining PARP inhibitors and camptothecins have not translated into clinical benefit due to enhanced toxicity with the combination [13, 14]. In combination with chemotherapy, olaparib and veliparib enhanced the myelosuppression of their chemotherapy partners. In these studies, unacceptable toxicity necessitated dose reductions to sub-therapeutic levels.

1.2.4 Safely combining PARP Inhibitors with Camptothecins

One approach to safely combine camptothecins with agents that sensitize their activity like PARP inhibitors is to use alternative formulations that could minimize toxicity to the normal tissues.

CRLX101 (formerly named IT-101) is a nanoparticle-drug conjugate (NDC) composed of 20(S) -camptothecin conjugated to a linear, cyclodextrin-polyethylene glycol-based polymer (Poly-CD-PEG-Camptothecin) (CRLX101 Investigators Brochure) In solution, CRLX101 self-assembles into nanoparticles and releases CPT over prolonged periods of time [15] [16].

Camptothecin (CPT) is a potent topoisomerase I (Top1) inhibitor that failed clinical development due to poor solubility and high systemic toxicity. CPT derivatives such as irinotecan and topotecan demonstrate clinical utility for the treatment of advanced solid tumors. The primary cellular target of CPT, the Top1–DNA cleavage complex, is stabilized, preventing Top1-mediated DNA relegation. Exposure of cancer cells to CPT leads to replication-mediated accumulation of DNA double-strand breaks and subsequent apoptosis.

CRLX101 localizes to the tumor and is retained for a prolonged duration by a mechanism referred to as enhanced permeability and retention (EPR) [17] wherein macromolecules penetrate and are trapped in tumor tissue due to the abnormally leaky vasculature of tumors. CRLX101 provides sustained release of CPT from polymer in the tumor for prolonged periods while sustaining low unconjugated (released) CPT concentrations in the blood. Animal xenograft models of cancer indicate that CRLX101 accumulates in solid tumors and releases CPT over a period of several days to give sustained inhibition of its target [16]. Sustained release of camptothecin from polymer in the tumor and low unconjugated camptothecin concentrations in the blood could optimize the cell cycle specific antitumor activity associated with the prolonged drug exposure in tumor tissue and improve tolerability associated with low systemic exposure. Drug localization analyses have been conducted in tumor from subjects with HER-2 negative gastric cancer who enrolled in a clinical trial of CRLX101. Pre- and post- treatment biopsies involving tumor tissue and healthy adjacent tissue were collected from 10 subjects, 9 of whom had evaluable biopsies. Biopsies from all subjects were analyzed for differential drug accumulation between tumor and normal tissue using immunofluorescence techniques. Post-treatment biopsies were obtained between 24 and 48 hours after a single dose of CRLX101 and CPT was visualized by direct fluorescent excitation of tissues. Of the 9 patients with evaluable biopsies, 7 showed clear evidence of CPT within the post-treatment tumor while only one patient showed potential evidence of CPT in the post-treatment normal tissue. Images show bright, punctuate CPT signal specific to tumor tissue following treatment with CRLX101 and rare events of CPT signal co-localized with anti-PEG signal as demonstrated in **Figure 2.**

Figure 2: Immunofluorescence Visualization in Gastric Cancer Tumor and Adjacent Normal Tissue after First Dose with CRLX101 in Subjects on the Gastric Investigator-Sponsored Trial

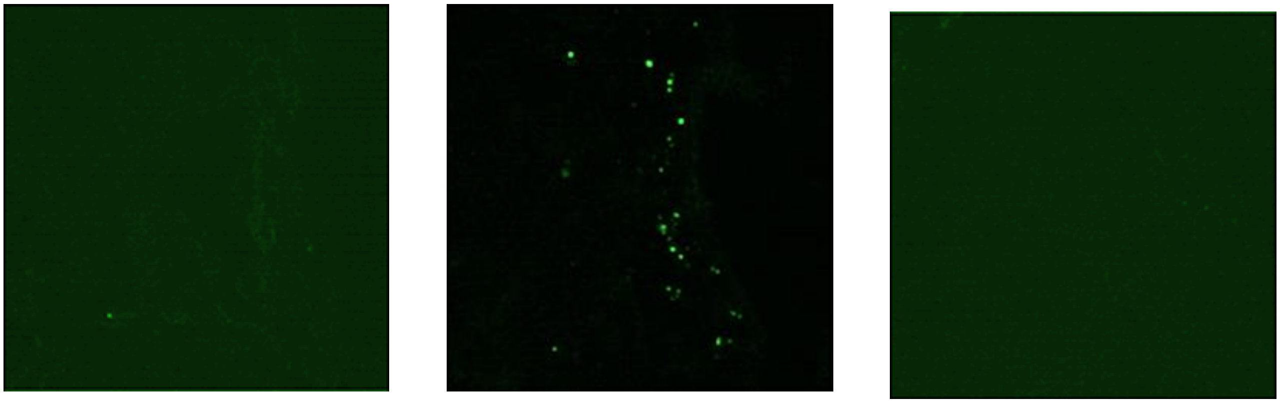

Left: Tumor pre-treatment; **Middle:** Tumor 24 hour post-treatment; **Right**: Healthy adjacent tissue 24 hours post; Green dots = CPT from nanoparticles

1.2.5 CRLX101 Preclinical Toxicology

Dose-limiting toxicity in rat and dog were largely body weight losses. Reductions in neutrophil and platelet counts were observed in the acute dog toxicity study and reversible reductions in neutrophil and platelet counts were observed at the mid dose in the sub-acute multi-dose dog toxicity study (CRLX101 Investigators Brochure). Reversible reductions in neutrophil and increases in platelet counts were observed at the high dose in the sub-acute multi-dose rat toxicity study.

1.2.6 CRLX101 phase I clinical trial

Patients with advanced solid malignancies were enrolled to an open-label, single-arm, dose-escalation study, in which CRLX101 was administered intravenously over 60 min among two dosing schedules, initially weekly at 6, 12, and 18 mg/m2 and later bi-weekly at 12, 15, and 18 mg/m2. The MTD was determined at 15 mg/m2 bi-weekly, and an expansion phase 2a study was completed. Sixty-two patients (31 male; median age 63 years, range 39-79) received treatment. Bi-weekly dosing was generally well tolerated with myelosuppression being the dose-limiting toxicity. Among all phase 1/2a patients receiving the MTD (*n*=44), most common grade 3/4 adverse events were neutropenia (Grade 3 neutropenia = 9% and no grade 4) and fatigue (Grade 3 fatigue = 9%, no grade 4). Evidence of systemic plasma exposure to both the polymer-conjugated and unconjugated CPT was observed in all treated patients. Mean elimination unconjugated CPT Tmax values ranged from 17.7 to 24.5 h, and maximum plasma concentrations and areas under the curve were generally proportional to dose for both polymer-conjugated and unconjugated CPT. Best overall response was stable disease in 28 patients (64 %) treated at the MTD and 16 (73 %) of a subset of NSCLC patients. Median progression-free survival (PFS) for patients treated at the MTD was 3.7 months and for the subset of NSCLC patients was 4.4 months.

1.2.7 Adverse Events with CRLX101 Monotherapy

Study CRLX-001 (Phase 1/2a Solid Tumors): The most common treatment emergent adverse events (TEAE) were fatigue (31 subjects, 50%; 9% grade 3 and 4; 1% grade 1/2), followed by nausea (29%; all grade ½; on the day of infusion and up to 2 days following infusion; stable after multiple cycles), cystitis (27%), constipation (24%), cough (24%), decreased appetite (23%), dysuria (23%), anemia (23%), dyspnea (21%), and back pain (21%). In addition there were 3 (5%) subjects reporting Grade 3/4 related TEAEs at time of infusion that occurred in one subject each: hypersensitivity, infusion-related reaction, and cytokine release syndrome (none received pre-medication).

Among the 44 subjects treated at the MTD, of whom 22 had NSCLC, the most common TEAEs were fatigue (20 subjects, 46%), constipation, nausea (12 subjects each, 27%), cystitis (11 subjects, 25%), back pain, and cough (9 subjects each, 21%).

Study CRLX-002 (Phase 2 NSCLC): In the CRLX101 plus BSC group, the most frequently reported TEAEs were anemia (25 subjects, 26%), dyspnea (17 subjects, 18%), cough (14 subjects, 14%), asthenia (12 subjects, 12%), nausea (11 subjects, 11%), cystitis (10 subjects, 10%) and fatigue (10 subjects, 10%)

Study CRLX101-208 (Phase 2 RCC): In the CRLX101 plus bevacizumab group, reported TEAEs associated with CRLX101 were anorexia, constipation, non-infective cystitis, dysuria, epistaxis, hematuria, hyponatremia, hypophosphatemia, nausea, pollakuria (urinary frequency), urinary urgency, and vomiting. In the CRLX101 group TEAEs reported by more than 1 subject included agitation, constipation and epistaxis.

Gastric Cancer investigator sponsored trial (IST): TEAEs considered related to CRLX101 included: anemia, fatigue, proteinuria, white blood cell count decrease, neutrophil count decrease, hematuria, urinary tract pain (dysuria), back pain, chest pain, chills, cough, decreased platelet count, dehydration, hypertension, increased alkaline phosphatase, increased AST, non-infective cystitis, infusion-related reaction, myalgia, nausea, pruritus, sinus tachycardia, pollakiuria (urinary frequency), and vomiting.

Small Cell Lung Cancer IST: TEAEs considered related to CRLX101 included: anemia, anorexia, constipation, dehydration, dizziness, dysgeusia, fatigue, hypoalbuminemia, hypokalemia, hypomagnesemia, hypophosphatemia, decrease white blood cell count, oral mucositis, nausea, platelet count decrease, vomiting, alkaline phosphate increase, alopecia, bilirubin increase, chronic kidney disease, diarrhea, facial pain, flu-like symptoms, generalized weakness, headache, hematuria, hyperhidrosis, hyperkalemia, hypermagnesemia, hypertension, hyponatremia, paresthesia, pruritus (maculopapular rash), small intestinal obstruction, supratherapeutic drug level (Lovenox), and tremor.

1.2.8 Adverse Events Associated with CRLX101 Combination Therapy

CRLX101 has not been thoroughly evaluated in clinical studies with other anticancer agents. Investigation of CRLX101 in combination therapy with bevacizumab is preliminary and has only been observed as part of ISTs. Please refer to the manufacturer’s package insert for the safety profile of bevacizumab.

Ovarian Cancer IST: Among monotherapy treated patients, related TEAEs reported by investigator included: anemia, nausea, fatigue, hematuria, hypomagnesemia, proteinuria, urinary

frequency, urinary tract pain, alanine aminotransferase increased, febrile neutropenia, and flatulence, allergic rhinitis, anorexia, bladder spasm, chills, noninfective cystitis, paresthesia (tingling), flatulence, flushing, hypophosphatemia, malaise, urinary tract pain (dysuria), pruritus (maculo-papular rash), sinus bradycardia and vasovagal reaction.

AEs reported in more than 30% of subjects included: anemia, constipation, diarrhea, fatigue and nausea. Other AEs reported in >10% of subjects included: abdominal pain, increased alkaline phosphatase, cough, dizziness, dyspnea, hypomagnesemia, infusion-related hypersensitivity reaction, neutropenia, pain, peripheral neuropathy and vomiting.

Among the patients treated with CRLX101 in combination with bevacizumab, related TEAEs included: anemia, fatigue, febrile neutropenia, hematuria, hypomagnesemia, increased alanine transaminase, nausea, proteinuria, urinary frequency, and urinary tract pain (dysuria), bloating, decreased platelet count, decreased white blood cell count, pyrexia (fever), flatulence, hemoglobinuria, hyperkalemia, hypertension, hyponatremia, hypophosphatemia, infusion reaction, increased alkaline phosphatase, increased aspartate transaminase, increased creatinine, mucositis oral, peripheral motor neuropathy, thromboembolic event, and tooth infection.

Renal Cell Cancer IST*:*   Related TEAEs in multiple subjects included: anemia, abdominal pain, constipation, diarrhea, flatulence, vomiting, edema in limbs, fatigue, weight loss, urinary tract infection, dizziness, headache, noninfective cystitis, proteinuria and pruritus. Other TEAEs occurring in 1 subject only and considered related to CRLX101 included: agitation, bloating, dry eye, dry heaves, dyspepsia, hearing loss, dehydration, dental carries, dyspnea, hematuria, hypoalbuminemia, oral mucositis, oral pain, pleural effusion, skin hyperpigmentation, urinary frequency and voice alteration.

AEs occurring in more than 30% of subjects on this study were dizziness, fatigue and hypertension. Other AEs occurring in more than 10% (2 or more) of subjects included: abdominal pain, anorexia, confusion, constipation, cystitis, edema (lower extremity), headache, nausea, vomiting, and weight loss.

Neoadjuvant Chemoradiation in Rectal Cancer IST: Related TEAEs reported in multiple subjects included: anemia, abdominal pain, constipation, diarrhea, nausea, rectal hemorrhage, rectal pain, fatigue, lymphocyte count decreased, neutrophil count decreased, white blood cell decreased, urinary tract pain (dysuria), urinary frequency, and palmar-plantar erythrodysesthesia syndrome. Additional related TEAEs reported in only 1 subject included: cystitis, radiation dermatitis, gastrointestinal pain, generalized pain, non-cardiac chest pain, headache, hypokalemia, hypophosphatemia, increased alanine aminotransferase, increased aspartate aminotransferase and increased bilirubin level.

Hypersensitivity reactions

Infusion-related hypersensitivity reactions during CRLX101 infusion have been observed in a total of 13 of the 280 (5%) treated subjects. Three subjects experienced infusion-related hypersensitivity reactions in the Phase 1/2a trial (CRLX-001) (N=62), all 3 were reported as serious. None of these subjects received premedication and all had a history of drug allergy. Based on this observation, mandatory premedication was implemented per protocol amendment. 10 subjects out of the 218 (5%) who received the protocol required pre-medications in subsequent trials have reported infusion-related hypersensitivity reactions, all of which were Grade 1 or 2 and non-serious. All subjects with hypersensitivity reactions were medically managed with corticosteroids and recovered without

sequelae; subjects continued to receive CRLX101 after medical treatment and resolution of symptoms. CRLX101 infusion was either restarted on the same day or subsequently resumed at the next scheduled visit.

Several subjects with infusion-related hypersensitivity reactions had a history of drug allergy, including a history of allergy to anticancer agents, commonly taxanes and platinum-based agents. A careful review of the medical history is therefore warranted; however, the possibility of hypersensitivity reaction during CRLX101 infusion cannot be ruled out if the subject does not have a history of drug allergy.

1.2.9 CRLX101 Clinical Development Overview

The safety and efficacy information for CRLX101 contained herein is based on the data available as of 12 March 2015:

- Four clinical trials were initiated under the Sponsor's IND #71,694. Study CRLX-001 was completed and the clinical study report (CSR) was finalized; Study CRLX-002 was concluded and the CSR is pending. Studies CRLX101-208 and CRLX101-209 are ongoing.
- Five Investigator-sponsored trials have been initiated and/or completed.
- A total of 280 subjects across all studies have received at least 1 dose of CRLX101.
- 168 subjects received CRLX101 at doses ranging from 6 to 18 mg/m^2 in 3 sponsored trials (Studies CRLX-001, CRLX-002, and CRLX101-208).
- 112 subjects received at least 1 dose of CRLX101 in five ISTs at doses ranging from 12 to 15 mg/m^2.
- Of the 280 CRLX101-treated subjects, 253 (90%) received the MTD of 15 mg/m^2, and 220 (79%) received CRLX101 as monotherapy.
- The most common TEAEs in CRLX101-treated subjects include: anemia, constipation, fatigue, nausea, neutropenia, thrombocytopenia, leukopenia and cystitis. These were mostly grade 1 or 2.
- CRLX101 has shown preliminary antitumor activity in subjects with renal, rectal, gastroesophageal, lung, and ovarian cancers.

1.2.10 Olaparib

Olaparib is a PARP inhibitor indicated as monotherapy in patients with deleterious or suspected deleterious germline BRCA mutated (as detected by an FDA-approved test) advanced ovarian cancer who have been treated with three or more prior lines of chemotherapy. Olaparib has an established safety profile and it is under investigation in a number of different cancers.

Olaparib (AZD2281, KU-0059436) is a potent Polyadenosine 5'diphosphoribose [poly (ADP ribose] polymerisation (PARP) inhibitor (PARP-1, -2 and -3) that is being developed as an oral therapy, both as a monotherapy (including maintenance) and for combination with chemotherapy and other anti-cancer agents.

PARP inhibition is a novel approach to targeting tumors with deficiencies in DNA repair mechanisms. PARP enzymes are essential for repairing DNA single strand breaks (SSBs).

Inhibiting PARPs leads to the persistence of SSBs, which are then converted to the more serious DNA double strand breaks (DSBs) during the process of DNA replication. During the process of cell division, DSBs can be efficiently repaired in normal cells by homologous recombination repair (HR). Tumors with HR deficiencies (HRD), such as serous ovarian cancers and SCLC cannot accurately repair the DNA damage, which may become lethal to cells as it accumulates. In such tumor types, olaparib may offer a potentially efficacious and less toxic cancer treatment compared with currently available chemotherapy regimens.

Olaparib has been shown to inhibit selected tumor cell lines in vitro and in xenograft and primary explant models as well as in genetic BRCA knock-out models, either as a stand-alone treatment or in combination with established chemotherapies. Cells deficient in homologous recombination DNA repair factors, notably BRCA1/2, are particularly sensitive to olaparib treatment.

PARP inhibitors such as olaparib may also enhance the DNA damaging effects of chemotherapy [18-20]. For further information please refer to the current version of the olaparib Investigator Brochure.

1.2.11 Preclinical Data of CRLX101 plus Olaparib

In vivo studies (conducted by Astra Zeneca) show that concurrent combination of CRLX101 and olaparib resulted in a dose-dependent decrease in hematological parameters in preclinical rat bone marrow model. However, combination with delay (24h+) between CRLX101 and olaparib provided sparing effect on peripheral blood cells (nadir and recovery) (**Figure 3**).

The combination of CRLX101 with olaparib (starting 24 hours after CRLX101) administered for 14 days had significantly more anti-tumor activity than olaparib administered for 2 days and provided greater efficacy than CRLX101 alone (**Figure 4**). The combination was also significantly more efficacious than topotecan (standard-of-care for SCLC).

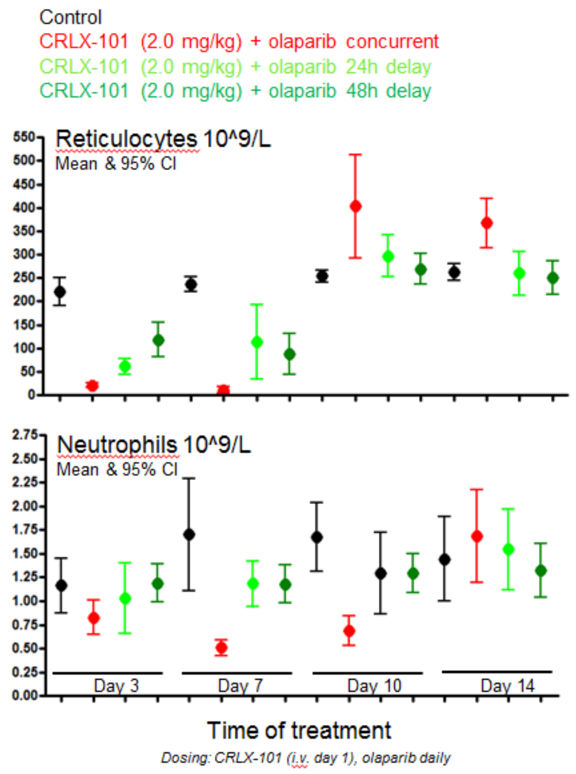

Figure 3: Hematological response to sequenced versus concurrent combination schedule in Wistar rat. Animals were treated with a single dose of CRLX101 and either simultaneously or with 24h and 48h delay with daily olaparib. Peripheral blood was collected from tail vain and analysis was performed on the Siemens Advia 2120i hematology analyzer.

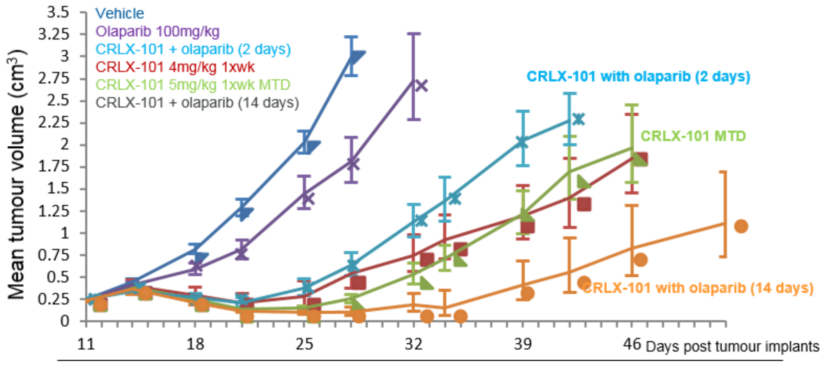

Figure 4: In vivo efficacy of CRLX101 combination with various duration of olaparib dosing in SCLC xenograft model. Mice implanted with NCI-H417a tumor cells were dosed with single dose of CRLX101 + olaparib for 2 days or 14 days starting 24h after the CRLX101.

1.2.12 Hypothesis

Despite their highly synergistic activity in preclinical models, human studies combining PARP inhibitors and camptothecins have not translated into clinical benefit due to enhanced toxicity with the combination. CRLX101 (and free camptothecin) is cleared out more slowly from tumor cells compared to plasma/bone marrow and induces sustained activation of DNA damage response in tumor. Of note, in preclinical models, most CRLX101 is cleared from the bone marrow by 24 hours; in contrast, durable topoisomerase 1 inhibition in the tumor is apparent. This effect combined with low systemic exposure lends CRLX101 to combinations with PARP inhibitors where both drugs can potentially be used at effective doses. Based on preclinical

studies, PARP inhibitors used 48 hours after CRLX101 administration will provide synergy while minimizing risk of myelosuppression.

1.2.13 Rationale

PARP inhibitors are highly synergistic in combination with camptothecins in preclinical models. However, this has not translated to clinical effectiveness, primarily due to enhanced toxicity which precludes administration of effective doses of either the PARP inhibitor or chemotherapy. Major dose limiting toxicity of this combination is myelosuppression. CRLX101 is a nanoparticle drug conjugate with a camptothecin payload that provides durable inhibition of topoisomerase I (Top1) specifically in the tumor. CRLX101 is cleared from the bone marrow relatively quickly. These properties allow combination of CRLX101 with PARP inhibitors where both drugs can potentially be used at doses close to the MTD of each. Based on preclinical studies, PARP inhibitors used 48 hours after CRLX101 and continued for 11 days will provide maximum synergy while minimizing risk of myelosuppression.

Since the safety of this combination is not established, we propose a phase I trial where escalating doses of olaparib will be combined with CRLX101 administered at 80% of its MTD (12 mg/kg IV q2 weeks). The starting dose of olaparib is 100 mg bid which is a third of its single-agent maximum tolerated dose (MTD; 300 mg tablet BID).

2 ELIGIBILITY ASSESSMENT AND ENROLLMENT

2.1 Eligibility Criteria

2.1.1 Inclusion Criteria - Phase I

2.1.1.1 Patients must have advanced solid tumor that is resistant or refractory to standard therapy. Histologic or cytologic diagnosis should be confirmed in NCI Laboratory of Pathology.

2.1.1.2 A minimum of 2 weeks will be required from any prior therapy, including chemotherapy, immunotherapy and/or radiation. In addition, recovery to Grade ≤ 1 from all reversible toxicities related to prior therapy is required at study entry.

2.1.1.3 Patients do not need to have measurable disease to enroll on phase I.

2.1.1.4 Age ≥18 years.

2.1.1.5 ECOG performance status ≤2 (Appendix A, Section 13.1).

2.1.1.6 Patients with treated brain metastases (surgery, whole or stereotactic brain radiation) are allowed provided the lesions have been stable for at least 2 weeks and the patient is off steroids or is on a stable dose of steroids. Patients with brain metastases should not require use of enzyme-inducing antiepileptic drugs (e.g., carbamazepine, phenytoin, or phenobarbital) within 14 days before first dose and during study. Use of newer antiepileptics that do not produce enzyme induction drug-drug interactions (DDIs) is allowed.

2.1.1.7 Patients must have normal organ and marrow function ≤5 days prior to C1D1 as defined below:

– leukocytes	≥3,000/mcL
– absolute neutrophil count	≥1,500/mcL without growth factor support
– platelets	≥100,000/mcL without growth factor support
– hemoglobin	≥9 gr/dl
– total bilirubin	≤1.5 x ULN (unless Gilbert's Disease)
– AST(SGOT)/ALT(SGPT)	≤2.5 X institutional upper limit of normal (≤ 5X ULN if liver mets)
– creatinine	within normal institutional limits
	OR
– creatinine clearance	≥50 mL/min (calculated using the Cockroft-Gault formula) for patients with creatinine levels above institutional normal.

– No features suggestive of MDS/AML on peripheral blood smear

2.1.1.8 The effects of CRLX101 and olaparib on the developing human fetus are unknown. For this reason and because these agents as well as other therapeutic agents used in this trial

are known to be teratogenic, women of child-bearing potential and men must agree to use adequate contraception (hormonal or barrier method of birth control; abstinence) prior to study entry, for the duration of study participation and for 120 days (both male and female) following last dose of study drug. Should a woman become pregnant or suspect she is pregnant while she or her partner is participating in this study, she should inform her treating physician immediately. Fertile females of childbearing potential are defined as women physically capable of becoming pregnant unless the female patient cannot have children because of surgery or other medical reasons (effective tubal ligation, ovaries or the uterus removed, or are post-menopausal). Post-menopausal is defined as:

- Amenorrheic for 1 year or more following cessation of exogenous hormonal treatments,
- LH and FSH levels in the post menopausal range for women under 50,
- radiation-induced oophorectomy with last menses >1 year ago,
- chemotherapy-induced menopause with >1 year interval since last menses,
- or surgical sterilization (bilateral oophorectomy or hysterectomy).

2.1.1.9 Negative urine pregnancy test ≤3 days prior to C1D1 (women of childbearing potential only)

2.1.1.10 Patient is willing and able to comply with the protocol for the duration of the study including undergoing treatment and scheduled visits and examinations including follow up.

2.1.2 Inclusion Criteria - Phase II

2.1.2.1 Age ≥18 years.

2.1.2.2 Patients must have histologically or cytologically confirmed diagnosis of SCLC. Pathologic diagnosis should be confirmed in NCI Laboratory of Pathology.

2.1.2.3 Have received and progressed during or after a platinum-based standard chemotherapy regimen for first line treatment of SCLC, either limited stage or extensive stage.

2.1.2.4 Patients could have received any number of therapies for relapsed or progressive disease, including re-treatment with original frontline regimen. A minimum of 2 weeks will be required from any prior therapy, including chemotherapy, immunotherapy and/or radiation. In addition, recovery to Grade ≤ 1 from all reversible toxicities related to prior therapy is required at study entry. No previous irradiation to the site of measurable or evaluable disease, unless that site had subsequent evidence of progression.

2.1.2.5 Patients must have measurable disease as per Response Evaluation Criteria in Solid Tumors, version (RECIST 1.1) . See Section **6.3** for the evaluation of measurable disease.

2.1.2.6 Radiographic evidence of disease progression after initial therapy should have been documented.

2.1.2.7 ECOG performance status ≤2 (see Appendix A, Section **13.1**).

2.1.2.8 Patients with treated brain metastases (surgery, whole or stereotactic brain radiation) are allowed provided the lesions have been stable for at least 2 weeks and the patient is off steroids or is on a stable dose of steroids. Patients with brain metastases should not require use of enzyme-inducing antiepileptic drugs (e.g., carbamazepine, phenytoin, or phenobarbital) within 14 days before first dose and during study. Use of newer antiepileptics that do not produce enzyme induction drug-drug interactions (DDIs) is allowed.

2.1.2.9 Patients must have normal organ and marrow function ≤5days prior to C1D1 as defined below:

– leukocytes	≥3,000/mcL
– absolute neutrophil count	≥1,500/mcL without growth factor support
– platelets	≥100,000/mcL without growth factor support
– hemoglobin	≥9 g/dl
– total bilirubin	≤1.5 x ULN (unless Gilbert’s Disease)
– AST(SGOT)/ALT(SGPT)	≤2.5 X institutional upper limit of normal (≤

	5X ULN if liver mets)
– creatinine	within normal institutional limits
	OR
– creatinine clearance	≥50 mL/min (calculated using the Cockroft-Gault formula) for patients with creatinine levels above institutional normal.

– No features suggestive of MDS/AML on peripheral blood smear

2.1.2.10 The effects of CRLX101 and olaparib on the developing human fetus are unknown. For this reason and because these agents are known to be teratogenic, women of child-bearing potential and men must agree to use adequate contraception (hormonal or barrier method of birth control; abstinence) prior to study entry, for the duration of study participation and for 120 days (both male and female) following last dose of study drug. Should a woman become pregnant or suspect she is pregnant while she or her partner is participating in this study, she should inform her treating physician immediately. Fertile females of childbearing potential are defined as women physically capable of becoming pregnant unless the female patient cannot have children because of surgery or other medical reasons (effective tubal ligation, ovaries or the uterus removed, or are post-menopausal). Post-menopausal is defined as:

- Amenorrheic for 1 year or more following cessation of exogenous hormonal treatments,
- LH and FSH levels in the post menopausal range for women under 50,
- radiation-induced oophorectomy with last menses >1 year ago,
- chemotherapy-induced menopause with >1 year interval since last menses,
- or surgical sterilization (bilateral oophorectomy or hysterectomy).

2.1.2.11 Negative urine pregnancy test ≤3 days prior to C1D1 (women of childbearing potential only).

2.1.3 Exclusion Criteria - Phase I and II

2.1.3.1 Patients who are receiving any other investigational agents.

2.1.3.2 Persistent toxicities (≥ CTCAE grade 2) with the exception of alopecia, caused by previous cancer therapy

2.1.3.3 Blood transfusions within 1 month prior to study start

2.1.3.4 Patients with myelodysplastic syndrome/acute myeloid leukemia or pneumonitis

2.1.3.5 Hypersensitivity to study therapies and its excipients

2.1.3.6 Patients unable to swallow orally administered medication and patients with gastrointestinal disorders likely to interfere with absorption of the study medication.

2.1.3.7 History of allergic reactions attributed to compounds of similar chemical or biologic composition to CRLX101 and/or olaparib or other agents used in study.

2.1.3.8 Patients receiving any medications or substances that are strong and moderate inhibitors or inducers of CYP3A are ineligible. Lists including medications and substances known or with the potential to interact with the CYP3A isoenzymes are provided in Appendix B, Section **13.2**.

2.1.3.9 Pregnant women are excluded from this study because CRLX101 and/or olaparib are agents with the potential for teratogenic or abortifacient effects. Because there is an unknown but potential risk for adverse events in nursing infants secondary to treatment of the mother with CRLX101 and/or olaparib, breastfeeding should be discontinued if the mother is treated with CRLX101 and/or olaparib. These potential risks may also apply to other agents used in this study.

2.1.3.10 HIV-positive patients on combination antiretroviral therapy are ineligible because of the potential for pharmacokinetic interactions with CRLX101 and/or olaparib. In addition, these patients are at increased risk of lethal infections when treated with marrow-suppressive therapy. Appropriate studies will be undertaken in patients receiving combination antiretroviral therapy when indicated.

2.1.3.11 Prolongation of QT/QTc interval (QTc interval >500 msec) using the Fredericia method of QTc analysis or family history of long QT syndrome. If single reading is above these minimum ranges, then repeat test in triplicate and evaluate eligibility based on average value

2.1.3.12 Any chronic or concurrent acute liver disease.

2.1.3.13 Concurrent treatment with Coumadin. Use of low molecular weight heparin use is permitted.

2.1.3.14 History of stroke, transient ischemic attack (TIA), or myocardial infarction, within 6 months prior to C1D1

2.1.3.15 Uncontrolled concurrent disease or illness including but not limited to:

- symptomatic congestive heart failure, unstable angina pectoris, clinically significant cardiac arrhythmia
- unstable or untreated cardiac conditions or ejection fraction of <50% as determined by echocardiogram (ECHO) or multiple gated acquisition scan (MUGA)
- uncontrolled diabetes mellitus
- psychiatric illness that would limit compliance with study requirements, as determined by the Investigator

2.1.3.16 Other severe, acute, or chronic medical or psychiatric condition or laboratory abnormality that may increase the risk associated with study participation or study drug administration or that may interfere with the interpretation of study results and, in the judgment of the investigator, would make the patient inappropriate for the study.

2.2 Screening Evaluation

Screening must be completed within 2 weeks prior to C1D1.

2.2.1 History and physical exam (including height, weight, vital signs and performance status)

2.2.2 Blood tests (for organ function)

- Complete blood count (CBC/Diff)
- Acute care panel
- Hepatic panel
- Prothrombin time (PT)
- Partial thromboplastin time (PTT)

2.2.3 Viral Markers Protocol Screen (HBsAg, anti-HCV, anti-HIV) within 3 months of C1D1.

2.2.4 Confirmation of diagnosis by the NCI Laboratory of Pathology

2.2.5 CT chest/abdomen/ pelvis

2.2.6 Electrocardiogram

2.2.7 Echocardiogram

2.2.8 Urine or serum HCG for women of child-bearing potential (to be performed within 3 days of study enrollment)

2.3 Registration Procedures

Authorized staff must register an eligible candidate with NCI Central Registration Office (CRO) within 24 hours of signing consent. A registration Eligibility Checklist from the web site (http://home.ccr.cancer.gov/intra/eligibility/welcome.htm) must be completed and faxed to 301-480-0757. After confirmation of eligibility at Central Registration Office, CRO staff will call pharmacy to advise them of the acceptance of the patient on the protocol prior to the release of

any investigational agents. Verification of Registration will be forwarded electronically via e-mail to the research team. A recorder is available during non-working hours.

2.4 STRATIFICATION PROCEDURES

Subjects enrolled in the phase 2 cohort will be stratified according to sensitivity to initial chemotherapy. The chemotherapy sensitive (S) cohort will comprise patients with tumor progression that occurs 90 days or more after the last dose of initial chemotherapy. The resistant/refractory (RR) cohort will comprise patients with tumor progression that occurs less than 90 days after the last day of initial chemotherapy and patients with tumor progression during the initial chemotherapy or did not respond to initial chemotherapy. Up to 20 evaluable patients will be enrolled in each cohort following the optimal two-stage phase II trial design described in section **8**.

2.5 BASELINE EVALUATION

Please refer to the Study Calendar (Section **3.4**)

3 STUDY IMPLEMENTATION

3.1 STUDY DESIGN

This is a phase 1 / 2 open label single center trial. Patients will be enrolled to the phase 1 portion of the study in up to 5 dose levels to determine the maximum tolerated dose (MTD) or recommended phase 2 dose (RP2D). Once the MTD or RP2D is determined, up to 20 evaluable patients each will be enrolled to one of two cohorts: cohort S – chemotherapy sensitive or cohort RR – chemotherapy refractory/resistant as described in section **8**.

Patients meeting the eligibility criteria will receive CRLX101 (IV Q 2weeks) plus olaparib (PO BID days 3-13* and days 17-26* administered in 28 day cycles, until disease progression or development of intolerable side effects.

* On days 13 and 26, only one dose of olaparib will be administered in the morning.

Blood, tumor and hair samples will be collected at multiple time points for PK, PD analyses as described in **Table 7.** Tumor biopsies are optional; will be performed only in SCLC patients (in phase I and II) at the following time points: pre-treatment, on cycle 1 day 4 and at disease progression. Patients will be asked to consent to the optional biopsy at the time of the procedure. If the patient chooses not to have the biopsy, that will be documented in the medical record and noted in the research record.

Toxicity will be graded according to CTCAE version 4.0. Tumor assessments will be made using CT scans (chest, abdomen and pelvis) at baseline and after every 2 cycles according to RECIST version 1.1. Tumor assessments will be discontinued at the time of progression. Subsequently follow-up for survival will be carried out every 3 months.

Dose level	CRLX101, mg/m2<br>(IV q 2 weeks<br>D1 and D15)	Olaparib tablet, mg<br>(PO BID Days 3-13* and 17-26*)

1	12	100
2	12	150
3	12	200
4	12	250
5	15	250
28 day cycles; biopsies pre-treatment and on day 4 (24 hours post- olaparib) and at disease progression; * On days 13 and 26, only one dose of olaparib will be administered in the morning.		

3.1.1 Dose Limiting Toxicity

During the phase 1 portion of the study subjects will be monitored for DLTs during the first cycle unless otherwise indicated. DLTs will be defined using the National Cancer Institute (NCI) CTCAE (Version 4). The following events, occurring during cycle 1 of the study combination, will be considered DLTs if deemed drug-related:

- Grade 4 neutropenia complicated by fever ≥ 38.5 °C (i.e. febrile neutropenia) and/or documented infection;
- Grade 4 neutropenia that does not resolve within 7 days*
- Grade 4 thrombocytopenia that does not resolve within 7 days* or any grade 3-4 thrombocytopenia complicated with hemorrhage;
- Grade 4 anemia that does not resolve within 7 days despite optimal therapy (withholding study drug and red blood cell transfusions);
- Inability to begin subsequent treatment course within 28 days of the scheduled date, due to study drug toxicity;
- Any grade 3-4 non-hematologic toxicity (except fatigue/asthenia < 2 weeks in duration; mucositis in subjects who have not received optimal therapy for mucositis; vomiting or diarrhea lasting less than 72 hours whether treated with an optimal anti-emetic or anti-diarrheal regimen or not; or alkaline phosphatase changes).

*Note: In the event of a Grade 4 neutropenia or thrombocytopenia, a full blood count must be performed no more than 7 days after the onset of the event to determine if a DLT has occurred. The subject will be closely monitored until resolution to Grade 3 or less.

3.1.2 Dose Escalation

Dose escalation will proceed in cohorts of 3–6 patients. The MTD is the dose level at which no more than 1 of up to 6 patients experience DLT during the DLT evaluation period, and the dose below that at which at least 2 (of ≤6) patients have DLT as a result of the drug. If a patient did not experience DLT and did not finish cycle 1 of treatment, he or she will not be evaluable for

toxicity and will be replaced in the dose level. Patients who do not complete the DLT evaluation period and do not have a DLT will be replaced.

The theoretical maximum number of subjects required to determine the MTD in the phase 1 portion of the study is 30 subjects (6 per dose level), although it is expected that as few as 15 subjects in 4 dose levels would be required to reach an MTD.

Dose escalation will follow the rules outlined in the **Table 1** below.

Table 1

**Number of Patients with DLT**<br><br>**at a Given Dose Level**	**Escalation Decision Rule**
0 out of 3	Enter up to 3 patients at the next dose level
≥ 2	Dose escalation will be stopped. This dose level will be declared the maximally administered dose (highest dose administered). Up to three (3) additional patients will be entered at the next lowest dose level if only 3 patients were treated previously at that dose.
1 out of 3	Enter up to 3 more patients at this dose level.<br>• If 0 of these 3 patients experience DLT, proceed to the next dose level.<br>• If 1 or more of this group suffer DLT, then dose escalation is stopped, and this dose is declared the maximally administered dose. UP to three (3) additional patients will be entered at the next lowest dose level if only 3 patients were treated previously at that dose.
≤1 out of 6 at highest dose level below the maximally administered dose	This is the MTD and is generally the recommended phase 2 dose. At least 6 patients must be entered at the recommended phase 2 dose.

3.2 DRUG ADMINISTRATION

3.2.1 CRLX101

CRLX101 will be diluted with 5% dextrose for injection (D5W) to a total volume of 500 mL and should be infused intravenously as described in section **11.1.6** over 60 minutes. Nothing else should be added to the bag.

The CRLX101 infusion should begin immediately after preparation and diluted CRLX101 infusion solution not used within 6 hours should be destroyed following institutional practices.

3.2.1.1 Premedication Prior to CRLX101 Treatment and Pre and Post Hydration

Subjects will receive up to 1,000 ml of 0.9% normal saline solution IV hydration before and after administration of CRLX101 to reduce risk of cystitis adverse reaction. Hydration levels should be adjusted as needed by the Investigator based on any underlying health conditions of the subject.

Subjects should be pre-medicated with the following 3 drug classes 30-60 minutes prior to start of CRLX101 infusion to reduce likelihood of hypersensitivity adverse reactions:

- a corticosteroid (dexamethasone 20 mg IV) 30-60 minutes prior to start of CRLX101
- an antihistamine (diphenhydramine 50 mg PO)
- an H2 antagonist (ranitidine 50 mg IV)

premedication with antiemetics such as a 5-HT3 receptor antagonist are to be administered to reduce the potential for nausea. Suggested antiemetics include dolasetron, granisetron or ondansetron 30-60 minutes prior to starting CRLX101 infusion.

The example medications, route of administration, and dose indicated should be the medication-of-choice if clinically feasible. It is acknowledged that the premedication regimen may be altered for patient safety during the study (for example, if a patient experiences a hypersensitivity/infusion reaction related to study drug, the premedication regimen may be altered for subsequent cycle dosing based on the investigator's discretion).

3.2.1.2 Additional prevention measurement to prevent hypersensitivity/infusion reaction

The CRLX101 entire dose is to be administered over approximately 60 minute infusion time. During the initial 10 minutes, the infusion rate should be slower to reduce the risk of infusion-related hypersensitivity reaction. The first ~40mL will be administered over 10 minutes, and then the infusion rate will be increased to allow for complete administration over 60 minutes.

For a patient with history of infusion reaction to other drugs, it's recommended that the first infusion is given over 2 hours of time course. If it is tolerated, then the infusion rate can be gradually increased in subsequent dosing to be given over 60 minutes.

3.2.2 Olaparib

Olaparib tablet at the appropriate dose level will be given orally on days 3-13 and days 17-26 twice daily (on days 13 and 26, only one dose of olaparib will be administered in the morning) . The correct number of 25 mg, 100 mg, 150 mg or 200 mg tablets comprising the appropriate dose should be taken at the same times each day with approximately 240 mL of water. A light snack (biscuits/ toast) is advised at the time of dosing to help alleviate nausea but no specific timing needs to be observed. The olaparib tablets should be swallowed whole and not chewed, crushed, dissolved or divided.

Olaparib will be dispensed at the start of each cycle. Patients will be provided with a pill diary (Appendix C, Section **13.3**), instructed in its use, and asked to bring it with them to each appointment.

If vomiting occurs shortly after the olaparib capsules are swallowed, the dose should only be replaced if all of the intact capsules can be seen and counted. Should any patient enrolled on the study miss a scheduled dose, the patient will be allowed to take the scheduled dose up to a maximum of 2 hours after that scheduled dose time. If greater than 2 hours after the scheduled dose time, the missed dose should not be taken, and the patient should take their allotted dose at the next scheduled time.

3.3 DOSE MODIFICATIONS

The dose levels and the general approach to dose modification of CRLX101 and olaparib are shown below. AEs should be treated with the appropriate maximum intervention, and dose reductions should be clearly documented in the note.

3.3.1 General Recommendation for Dose Modification

Table 2

Dose Level	Olaparib dosage schedule		
	Starting dose	First dose reduction	Second dose reduction
-1	100 mg twice daily	75 mg twice daily	50 mg twice daily
1	100 mg twice daily	75 mg twice daily	50 mg twice daily
2	150 mg twice daily	100 mg twice daily	75 mg twice daily
3	250 mg twice daily	200 mg twice daily	150 mg twice daily
4 and 5	300 mg twice daily	250 mg twice daily	200 mg twice daily

Table 3

Dose Level	CRLX101 dosage schedule		
	Starting dose	First dose reduction	Second dose reduction
-1	9 mg/m2 q 2weeks	6 mg/m2 q 2weeks	4 mg/m2 q 2weeks
1-4	12 mg/m2 q 2weeks	9 mg/m2 q 2weeks	6 mg/m2 q 2weeks
5	15 mg/m2 q 2weeks	11 mg/m2 q 2weeks	7 mg/m2 q 2weeks

3.3.2 Dosing Delays/Dose Modifications and Management of Toxicities

- In the case of toxicity, appropriate medical treatment should be used (including anti-emetics, anti-diarrheals, etc.).
- Once a patient has a dose reduction for toxicity, the dose will not be increased.
- A maximum of 2 dose reductions is permitted
- If either agent is discontinued due to toxicity, the patient may continue on the other agent.
- Participants continuing to experience toxicity at the off treatment visit will be contacted for additional assessments until the toxicity has resolved or is deemed irreversible. Patients must remain on the study to have additional assessment.
- For AEs that are unrelated to the study drugs, study drug may be held for up to 21 days at the discretion of the PI.
- The associate investigator and/or PI will determine whether one or both drugs are responsible for an observed toxicity and will manage that toxicity as described below.

3.3.2.1 Hematologic toxicities

Table 4. Dose Modification and Management of Hematologic Adverse Events

Observation	Action
<u>On day 1 and 15</u><br>Absolute neutrophil count (ANC) ≥ 1500/mcL<br>**AND**<br>Platelets ≥ 75,000/mcL<br>**AND**<br>Hemoglobin ≥ 8 mg/dL	No interruption.
<u>On day 1 and 15</u><br>ANC < 1500/mcL<br>**OR**<br>Platelets < 75,000/mcL<br>**OR**<br>Hemoglobin < 8 mg/dL	*On first occurrence*, hold the drug(s) causing the toxicity for up to 21 days until ANC ≥ 1500/mcL, platelets ≥ 75,000/mcL, and hemoglobin ≥ 9 mg/dL.<br>The associate investigator and/or PI will determine whether one or both drugs are responsible for an observed toxicity and will initiate appropriate medical therapy and no change in dose upon re-initiation.<br>*On second occurrence*, hold the drug(s) causing the toxicity for up to 21 days until ANC ≥ 1500/mcL, platelets ≥ 75,000/mcL, and hemoglobin ≥ 9 mg/dL. Initiate appropriate medical therapy. Treatment with olaparib or CRLX 101 may be restarted at one dose level (DL) lower.<br>Patients whose counts have not recovered to ANC ≥ 1500/mcL, platelets ≥ 75,000/mcL, and hemoglobin ≥ 9 mg/dL after 21 days should be removed from the

	drug causing the toxicity.
<u>At any time during cycle</u><br>Grade 4 hematologic AE	*On first occurrence,* hold the drug(s) causing the toxicity for up to 21 days until ANC ≥ 1500/mcL, platelets ≥ 75,000/mcL, and hemoglobin ≥ 9 mg/dL. Initiate appropriate medical therapy. Treatment with olaparib or CRLX 101 may be restarted at one DL lower.<br>*On second occurrence,* discontinue the drug(s) causing the toxicity. Follow patient until resolution/stabilization of toxicity.<br>Patients whose counts have not recovered to ANC ≥ 1500/mcL, platelets ≥ 75,000/mcL, and hemoglobin ≥ 9 mg/dL after 21 days should be removed from the drug causing the toxicity. See section **3.3.2.1** for exceptions.
Toxicities requiring more than 2 dose reductions	Remove patient from the drug causing the toxicity.

- Treatment may be delayed for a maximum of 21 days after holding the treatment for toxicities that develop and do not resolve as defined above (exemptions: lymphopenia, or leukopenia in the absence of grade 3 or higher neutropenia).
- Weekly blood counts will be obtained during the first cycle, and then at day 1 and 15 of subsequent cycles. If any weekly evaluation demonstrates grade ≥ 3 neutropenia or grade ≥ 2 thrombocytopenia, a repeat hematology assessment will be obtained 2-4 days later.
- Management of prolonged hematological toxicities while on study treatment

    If a patient develops prolonged hematological toxicity such as:
    ≥2 week interruption/delay in study treatment due to CTC grade 3 or worse anemia and/or development of blood transfusion dependence
    ≥3 week interruption/delay in study treatment due to CTC grade 3 or worse neutropenia (ANC < 1 x 109/L)
    ≥3 week interruption/delay in study treatment due to CTC grade 3 or worse thrombocytopenia (Platelets < 50 x 109/L)

    Weekly differential blood counts including reticulocytes (calculate reticulocyte index (RI), RI = reticulocyte count x hematocrit (Hct)/normal Hct; a value of 45 is usually used for normal Hct) and peripheral blood smear should be performed. If any blood parameters remain clinically abnormal after 4 weeks of dose interruption, the patient should be referred to hematologist for further investigations. Bone marrow analysis and/or blood cytogenetic analysis should be considered at this stage according to standard hematological practice.

Development of a confirmed myelodysplastic syndrome or other clonal blood disorder should be reported as an SAE. Study treatment should be discontinued if diagnosis of myelodysplastic syndrome is confirmed.

3.3.2.2 Neutropenia

- Growth factors to prevent neutropenia will not be administered prophylactically and during the DLT period in phase I, but can be used during a drug hold to assist the recovery. Please note that G-CSF should not be used within at least 24 hours of the last dose of study treatment.

3.3.2.3 Thrombocytopenia

- Thrombocytopenia will be treated conservatively. In the absence of bleeding, or a necessary invasive procedure, platelet transfusions should be given for a platelet count ≤ 10,000/mcL.
- If invasive procedure(s) is (are) planned, or the patient develops bleeding, platelet transfusions should be administered in accordance with the standard of practice, usually maintaining a platelet count above 50,000/mcL.

3.3.2.4 Anemia

- Symptomatic anemia should be treated with red blood cell transfusion and is recommended if the hemoglobin falls below 8 g/dL or the patient is symptomatic.

3.3.3 General Recommendations for Dose Modification and Management of Non-Hematologic Adverse Events

- The management of general AEs not otherwise specified in the following sections should be as per **Table 5**.
- At the discretion of the investigator, the study drugs may be held or dose modified independently if the observed toxicity is attributed to only one of the drugs, while the patient continued to receive the drug not associated with the observed toxicity.
- Dose modifications for nausea, vomiting, and diarrhea will be made only if they are refractory to treatment. The time a given drug is held should not exceed 21 days.

Table 5. General Management of Adverse Events (Non-Hematologic)

Observation	Action
Grade 1 or 2 AEs resolves promptly (within 48 hours) with supportive care	Maintain dose level (DL)
Any ≥ grade 3 non-hematologic*	Hold study drug(s) causing the toxicity for up to 21 days until toxicity resolves to ≤ grade 1. Treatment with olaparib or CRLX101 may be restarted at one DL lower, as per the dose

	reduction guidelines.
Grade 3 or 4 non-hematologic AE related to drugs that does not resolve to grade 1 or less within 21 days despite maximum supportive care after treating patient at the lowest reduced DL	Remove patient from the drug causing the toxicity.
Toxicities requiring more than 2 dose reductions	Remove patient from the drug causing the toxicity.

*Except fatigue/asthenia < 2 weeks in duration; mucositis in subjects who have not received optimal therapy for mucositis; vomiting or diarrhea lasting less than 72 hours whether treated with an optimal anti-emetic or anti-diarrheal regimen or not; or alkaline phosphatase changes

- Management of new or worsening pulmonary symptoms

If new or worsening pulmonary symptoms (e.g. dyspnea) or radiological abnormality occurs, an interruption in olaparib dosing is recommended and a diagnostic workup (including a high resolution CT scan) should be performed, to exclude pneumonitis. Following investigation, if no evidence of abnormality is observed on CT imaging and symptoms resolve, then olaparib treatment can be restarted, if deemed appropriate by the investigator.

- Olaparib should be stopped before surgery and re-started after wound has healed following recovery.
- No stoppage of olaparib is required for any biopsy procedures.
- Olaparib should be discontinued for a minimum of 7 days before a patient undergoes therapeutic palliative radiation treatment.

3.3.4 Management of the hypersensitivity/infusion reactions

Table 6. Management of the hypersensitivity/infusion reactions

**Hypersensitivity reaction**	
Grade 1 or 2	**Stop** infusion immediately, continue hydration fluids and provide medications as indicated per institutional guidelines.<br>If symptoms resolve within 1-2 hours, at Investigator's discretion may re-start study drug administration at slower rate (i.e. 2x slower rate) and increase rate slowly to complete administration of full dose.<br>OR<br>**Hold** dose administration on day of event, and resume treatment at next

	scheduled day at same dose level.<br>At Investigator’s discretion may administer subsequent dose using a desensitization protocol as per institutional guidelines.
Grade 3 or 4	**Stop** infusion immediately and administer medical support as indicated per institutional guidelines.<br>**Hold** dose administration on day of event.<br>**Review** with study Principal Investigator to determine whether to discontinue from study drug treatment. Confirm that rate of administration was appropriate and premeds were given as suggested.

3.3.5 Laboratory safety assessment

Full hematology assessments for safety (hemoglobin, red blood cells [RBC], platelets, mean cell volume [MCV], mean cell hemoglobin concentration [MCHC], mean cell hemoglobin [MCH], white blood cells [WBC], absolute differential white cell count (neutrophils, lymphocytes, monocytes, eosinophils and basophils) and absolute neutrophil count or segmented neutrophil count and Band forms should be performed at each visit and when clinically indicated. If absolute differentials are not available, percentage differentials are acceptable. Coagulation [activated partial thromboplastin time (APTT) and international normalized ratio (INR)] will be performed at baseline and if clinically indicated unless the patient is receiving warfarin. Patients taking warfarin may participate in this study; however, it is recommended that prothrombin time (INR and APTT) be monitored carefully at least once per week for the first month, then monthly if the INR is stable.

Biochemistry assessments for safety (sodium, potassium, calcium, magnesium, fasting glucose, creatinine, total bilirubin, gamma glutamyltransferase [GGT], alkaline phosphatase [ALP], aspartate transaminase [AST], alanine transaminase [ALT], urea or blood urea nitrogen [BUN], total protein, albumin and lactic dehydrogenase [LDH]).

Urinalysis by dipstick should be performed at baseline and then only if clinically indicated. Microscopic analysis should be performed if required.

Bone marrow or blood cytogenetic samples may be collected for patients with prolonged hematological toxicities.

Additional analyses may be performed if clinically indicated.

Any clinically significant abnormal laboratory values should be repeated as clinically indicated.

3.4 STUDY CALENDAR

On study assessments can be performed within ±5 days of the specified time, unless otherwise indicated.

Procedure	Screening	Baseline[2]	Cycles = 28 days[1]										End of Treatment/ Disease Progression[13]	Post Therapy Follow-up[4]
			Day 1	2	3	4	5	7	15	21	28	Cycle 2 day 1		
History	X								X				X	
Physical exam[3]	X	X							X				X	
PTT/PT	X	X												
Viral Markers Protocol Screen[17]	X													
CBC diff[5]	X	X						X	X[14]				X	
Peripheral blood smear		X												
Acute Care Panel[6]	X	X							X[14]				X	
Hepatic panel[7]	X	X							X[14]				X	
Pregnancy test[8]	X	X										X		
CT scan[12]	X[15]	X											X	
Clinical disease assessment		X									X		X	
ECG	X	X												
Echocardiogram	X													
Confirmation of dx by NCI LP	X													
gH2AX blood and hair sample collection[9]			X		X	X								
Immune subsets			X		X	X						X		
Whole blood for nanostring/ CTC			X									X	X	
PK[16]			X	X	X				X					
Biopsies[10] (optional)		X				X							X	
CRLX101			X						X			X		
Olaparib[11]					X	X	X	X		X				
Follow-up phone call														X

[1]Number of cycles depends on disease progression and development of intolerable side effects

[2] Baseline procedures are performed within 2 weeks of initiation of study therapy with the exception of pregnancy test which must be performed within 3 days prior to the initiation of study therapy and ECG which should be obtained within 7 days prior to initiation of study

therapy. If the procedure was performed during the appropriate timeframe at screening, it is not necessary to repeat at baseline.

[3] Symptom-directed physical examinations will be performed as clinically indicated in the investigator's judgment

[4] Follow-up for survival will be carried out every 3 months

[5] Includes Neutrophils, Lymphs, Monos, Eos, Basos, WBC, RBC, Hemoglobin, Hematocrit, RBC Indices, MCV, RDW, Platelet. Results should be available prior to administration of study drugs

[6] Includes Sodium (NA), Potassium (K), Chloride (CL) Total CO2 (Bicarbonate), Creatinine, random Glucose, Urea nitrogen, eGFR

[7] Includes Alkaline Phosphatase, ALT/GPT, AST/GOT, Total Bilirubin, Direct Bilirubin

[8] Only for women of child-bearing potential

[9] Blood and hair sample will be collected at multiple time points during cycle 1 (pre-treatment on C1day1, pre-treatment on C1day3and pre-treatment on C1day4)

[10] Optional biopsies will be obtained at baseline, during the first treatment cycle (approximately 24 hours after the first dose of olaparib) and at disease progression.

[11] PO days 3-13 and days 17-26, as indicated in Section **3.2;** on days 13 and 26, only one dose of olaparib will be administered in the morning

[12] Performed after every 2 cycles

[13] Approximately 4 weeks after treatment discontinuation

[14] Only done in cycle 1

[15]Only done in phase 2

[16] Cycles 1 and 6 only

[17] Within 3 months of C1D1.

3.5 CRITERIA FOR REMOVAL FROM PROTOCOL THERAPY AND OFF STUDY CRITERIA

3.5.1 Criteria for Removal from Protocol Therapy

- Progressive disease
- Participant requests to be withdrawn from active therapy
- Unacceptable Toxicity as defined in Sections **3.1.1** and **3.3**
- Toxicity related dose delay lasting longer than 21 days in which case patient will be removed from the drug causing the toxicity.
- Investigator discretion
- Requirement for any of the prohibited study drugs as described in Appendix B, Section **13.2** (Strong and moderate Inhibitors and Inducers of CYP3A)
- The subject becomes pregnant

3.5.2 Off-Study Criteria

- Participant requests to be withdrawn from study
- Death

3.5.3 Off-Study Procedure

Authorized staff must notify Central Registration Office (CRO) when a subject is taken off-study. An off-study form from the web site (http://home.ccr.cancer.gov/intra/eligibility/welcome.htm) main page must be completed and faxed to 301-480-0757.

4 CONCOMITANT MEDICATIONS/MEASURES

- No other chemotherapy, hormonal therapy (HRT is acceptable) or other novel agent is to be permitted during the course of the study for any patient (the patient can receive a stable dose of corticosteroids during the study as long as these were started at least 4 weeks prior to treatment, as per exclusion criteria above). Palliative radiotherapy is allowed for pre-existing small areas of painful metastases that cannot be managed with local or systemic analgesics as long as no evidence of disease progression is present.
- Live virus and bacterial vaccines should not be administered whilst the patient is receiving study medication and during the 30 day follow up period. An increased risk of infection by the administration of live virus and bacterial vaccines has been observed with conventional chemotherapy drugs and the effects with olaparib are unknown.
- No concomitant use of alternative, complementary therapies or over-the-counter agents will be allowed without approval of the PI. All medications must be recorded in the case report form and be reviewed by the treating physician at each visit.
- Caution should be exercised in the concomitant use of any medication that may markedly affect renal function. Such medications may be used with caution as deemed essential for treatment, or if already in use prior to entry in the study without any effect on renal function.
- Caution should be exercised in concomitant use of any medication that may significantly affect hepatic CYP450 drug metabolizing activity by way of enzyme induction (e.g. Phenytoin) or inhibition (e.g. ketoconazole, ritonavir, erythromycin) within 2 weeks before the first dose of olaparib and throughout the study period.
- Given this data, potent inhibitors or inducers of CYP3A4 (as outlined in Appendix B, Section **13.2** must not be used during this study.
- CYP3A4 known potent inhibitors: wash-out period 1 week, or at least 4.5x elimination half-lives for drugs and metabolites known to inhibit CYP3A subfamily enzymes: Ketoconazole, itraconazole, ritonavir, indinavir, saquinavir, telithromycin, clarithromycin and nelfinavir
- CYP3A4 inducers (potential reduction in efficacy of olaparib) a wash-out period for 3 weeks for Phenytoin, rifampicin, rifapentin, rifabutin, carbamazepine, phenobarbital, nevirapine, modafinil and St John's Wart.
- Patients should avoid concomitant use of drugs, herbal supplements and/or ingestion of foods known to modulate CYP3A4 enzyme activity from the time they enter the screening period until 30 days after the last dose of study medication. *In vitro* data have

shown that the principal enzyme responsible for the formation of the 3 main metabolites of olaparib is CYP3A4 and consequently, this restriction is required to ensure patient safety.

- Olaparib inhibits CYP3A4 *in vitro* and is predicted to be a mild CYP3A inhibitor *in vivo*. Therefore, caution should be exercised when sensitive CYP3A substrates or substrates with a narrow therapeutic margin (e.g. simvastatin, cisapride, cyclosporine, ergot alkaloids, fentanyl, pimozide, sirolimus, tacrolimus and quetiapine) are combined with olaparib.
- Substrates of UGT1A1 should also be given with caution in combination with olaparib (e.g. irinotecan, nintedanib, ezetimibe, raltegravir or buprenorphine).
- Induction of CYP1A2, 2B6 and 3A4 has been shown in vitro with CYP3A4 CYP2B6 being most likely to be induced to a clinically relevant extent. The potential for olaparib to induce CYP2C9, CYP2C19 and P-gp is unknown. It cannot be excluded that olaparib upon co administration may reduce the exposure to substrates of these metabolic enzymes and transport protein. The efficacy of hormonal contraceptives may be reduced if co administered with olaparib.
- In vitro olaparib has been shown to be an inhibitor of P-gp, OATP1B1, OCT1, OCT2, OAT3, MATE1 and MATE2K and is a weak inhibitor of BRCP. It cannot be excluded that olaparib may increase the exposure to substrates of P-gp (e.g. statins, digoxin, dabigatran, colchicine), OATP1B1 (e.g. bosentan, glibenclamide, repaglinide, statins, and valsartan), OCT1 (e.g. metformin), OCT2 (e.g. serum creatinine), OAT3, MATE1 and MATE2K. In particular, caution should be exercised if olaparib is administered in combination with any statin.

4.1 SUPPORTIVE CARE

Patient should receive general concomitant and supportive care medications based on best medical practice.

The use of any natural/herbal products or other “folk remedies” should be discouraged.

Olaparib is an investigational drug for which no data on in vivo interactions are currently available. Based on in vitro data and clinical exposure data, olaparib is considered unlikely to cause clinically significant drug interactions through inhibition or induction of cytochrome P450 enzyme activity. In vitro data have, however, also shown that the principal enzyme responsible for the formation of the 3 main metabolites of olaparib is CYP3A4 and consequently, to ensure patient safety, the following potent inhibitors of CYP3A4 must not be used during this study for any patient receiving olaparib.

While this is not an exhaustive list, it covers the known potent inhibitors, which have most often previously been reported to be associated with clinically significant drug interactions:

- ketoconazole, itraconazole, ritonavir, idinavir, saquinavir, telithromycin, clarithromycin and nelfinavir

For patients taking any of the above, the required wash-out period prior to starting olaparib is one week.

In addition, to avoid potential reductions in exposure due to drug interactions and therefore a potential reduction in efficacy, the following CYP3A4 inducers should be avoided:

- Phenytoin, rifampicin, rifapentine, rifabutin, carbamazepine, phenobarbitone, nevirapine, modafinil and St John's Wort (*Hypericum perforatum*)

For patients taking any of the above, the required wash-out periods prior to starting olaparib are:

- phenobarbitone 5 weeks, and for any of the others, 3 weeks.

4.1.1 Other Concomitant Medications

All medications (prescriptions or over-the-counter medications) continued at the start of the trial or started during the study or until 30 days from the end of the last protocol treatment and different from the study medication must be documented.

Anticoagulant Therapy: Subcutaneous heparin is permitted.

4.1.2 Palliative radiotherapy

Palliative radiotherapy may be used for the treatment of pain at the site of bony metastases that were present at baseline, provided the Investigator does not feel that these are indicative of clinical disease progression during the study period. Study treatment should be discontinued for a minimum of 3 days before the patient undergoes palliative radiation treatment. Study treatment should be restarted within 21 days as long as any bone marrow toxicity has recovered.

4.1.3 Administration of other anti-cancer agents

Patients must not receive any other concurrent anti-cancer therapy, including investigational agents, while on study treatment. Patients may continue the use of bisphosphonates for bone disease and corticosteroids for the symptomatic control of brain metastases provided the dose is stable before and during the study

4.1.4 Medications that may NOT be administered

No other chemotherapy, immunotherapy, hormonal therapy or other novel agent is to be permitted while the patient is receiving study medication.

4.1.5 Overdose

There is currently no specific treatment in the event of overdose with olaparib and possible symptoms of overdose are not established.

Olaparib must only be used in accordance with the dosing recommendations in this protocol. Any dose or frequency of dosing that exceeds the dosing regimen specified in this protocol should be reported as an overdose.

Adverse reactions associated with overdose should be treated symptomatically and should be managed appropriately.

If an overdose on an AstraZeneca study drug occurs in the course of the study, then investigators or other site personnel must inform appropriate AstraZeneca representatives **within one day**, i.e.,

immediately but no later than **the end of the next business day** of when he or she becomes aware of it.

For overdoses associated with SAE, standard reporting timelines apply. For other overdoses, reporting should be done within 30 days.

4.1.6 Maternal exposure

If a patient becomes pregnant during the course of the trial, study drug should be discontinued immediately.

The outcome of any conception occurring from the date of the first dose until 3 months *after the last dose* should be followed up and documented.

Pregnancy itself is not regarded as an adverse event unless there is a suspicion that the investigational product under study may have interfered with the effectiveness of a contraceptive medication. Congenital abnormalities/birth defects and spontaneous miscarriages should be reported and handled as SAEs. Elective abortions without complications should not be handled as AEs. The outcome of all pregnancies (spontaneous miscarriage, elective termination, ectopic pregnancy, normal birth or congenital abnormality) should be followed up and documented even if the patient was withdrawn from the study.

If any pregnancy occurs in the course of the study, then Investigators or other site personnel must inform appropriate AstraZeneca representatives **within one day** i.e., immediately but no later than the **end of the next business day** of when he or she becomes aware of it.

4.1.7 Paternal exposure

Male patients should refrain from fathering a child or donating sperm during the study and for 3 months following the last dose.

Pregnancy of the patient’s partners is not considered to be an adverse event. However, the outcome of all pregnancies (spontaneous miscarriage, elective termination, ectopic pregnancy, normal birth or congenital abnormality) should if possible be followed up and documented.

The outcome of any conception occurring from the date of the first dose until 3 months *after the last dose* should be followed up and documented.

4.1.8 Pregnancy

All outcomes of pregnancy should be reported to AstraZeneca.

5 BIOSPECIMEN COLLECTION

5.1 Correlative Studies for Research/Pharmacokinetic Studies

Table 7.Correlative studies

Phase 1

Sample	Assay	Time points	Type of tube/sample	Amount of blood
Tumor (only in patients with SCLC)	gH2AX	Pre-treatment<br>C1D4 (24hr after olaparib)	NA	NA
	SLFN11 IHC<br>SLFN11 gene expression	Pre-treatment	NA	NA
	Exome/RNAseq/ droplet digital PCR	Pre-treatment<br>At disease progression	Purple top EDTA for germline DNA	5 ml
Hair<br>(in all phase I patients)	gH2AX	Pre-treatment on C1D1<br>Pre-treatment C1D3 (pre-olaparib)<br>C1D4 (24hr after olaparib)	12 follicles	12 follicles
Peripheral blood mononuclear cells	Immune subsets<br>(only in patients with SCLC)	Pre-treatment on C1D1<br>Pre-treatment C1D3 (pre-olaparib)<br>C1D4 (24hr after olaparib)<br>Pre-treatment C2D1	CPT citrate blue/black tubes	Two 8-ml tubes
	gH2AX<br>(in all phase I patients)	Pre-treatment on C1D1<br>Pre-treatment C1D3 (pre-olaparib)<br>C1D4 (24hr after olaparib)	green top	One 6-ml tube

Sample	Assay	Time points	Type of tube/sample	Amount of blood
Circulating tumor cells	Enumeration RAD51 foci<br>(only in patients with SCLC)	Pre-treatment on C1D1<br>Pre-treatment C2D1<br>At disease progression	Lavender top tubes<br><br>PAXgene RNA	Two 10-ml<br><br>One 2.5 ml
Whole blood<br>(only in patients with SCLC)	Nanostring immune panel (770 genes)	Pre-treatment on C1D1<br>Pre-treatment C2D1<br>At disease progression	PAXgene RNA	One 2.5 ml
	Nanostring DNA damage response panel (180 genes) [3,4]	Pre-treatment on C1D1<br>Pre-treatment C2D1<br>At disease progression	PAXgene RNA	One 2.5 ml
Plasma	PK	Refer to PK table in section **5.1.7**	Refer to PK table in section **5.1.7**	Refer to PK table in section **5.1.7**

Phase 2

Sample	Assay	Time points	Type of tube/sample	Amount of blood
Tumor	gH2AX	Pre-treatment<br>C1D4 (24hr after olaparib)	NA	NA
	SLFN11 IHC<br>SLFN11 gene expression	Pre-treatment	NA	NA

Sample	Assay	Time points	Type of tube/sample	Amount of blood
	Exome/RNAseq/ droplet digital PCR	Pre-treatment<br>At disease progression	Purple top EDTA for germline DNA	5 ml
Hair	gH2AX	Pre-treatment on C1D1<br>Pre-treatment C1D3 (pre-olaparib)<br>C1D4 (24hr after olaparib)	12 follicles	12 follicles
Peripheral blood mononuclear cells	Immune subsets	Pre-treatment on C1D1<br>Pre-treatment C1D3 (pre-olaparib)<br>C1D4 (24hr after olaparib)<br>Pre-treatment C2D1	CPT citrate blue/black tubes	Two 8-ml tubes
	gH2AX	Pre-treatment on C1D1<br>Pre-treatment C1D3 (pre-olaparib)<br>C1D4 (24hr after olaparib)	green top	One 6-ml tube
Circulating tumor cells	Enumeration<br>RAD51 foci	Pre-treatment on C1D1<br>Pre-treatment C2D1<br>At disease progression	Lavender top tubes<br><br>PAXgene RNA	Two 10-ml<br><br>One 2.5 ml

Sample	Assay	Time points	Type of tube/sample	Amount of blood
Whole blood	Nanostring immune panel (770 genes)	Pre-treatment on C1D1<br>Pre-treatment C2D1<br>At disease progression	PAXgene RNA	One 2.5 ml
	Nanostring DNA damage response panel (180 genes) [3,4]	Pre-treatment on C1D1<br>Pre-treatment C2D1<br>At disease progression	PAXgene RNA	One 2.5 ml
Plasma	PK	Refer to PK table in section 5.1.7	Refer to PK table in section 5.1.7	Refer to PK table in section 5.1.7

5.1.1 γ-H2AX

Phosphorylated H2AX (γ-H2AX) plays an important role in the recruitment and/or retention of DNA repair and checkpoint proteins such as BRCA1, MRE11/RAD50/NBS1 complex, MDC1 and 53BP1. DNA damage has been shown to increase H2AX phosphorylation in cancer cells following exposure to camptothecins. If olaparib is able to increase the degree of DNA damage due to CRLX101, it may be detectable by measurement of H2AX phosphorylation. We plan to study patient PBMCs, hair follicles, and tumor biopsies if there is readily accessible disease. Tumor and hair follicles will be obtained if patients are suitable candidates and willing to allow such sampling. The ideal biomarker would confirm that there was increased DNA damage following addition of olaparib relative CRLX101 alone. This analysis will be exploratory only and data will be used in planning biomarker endpoints in subsequent trials with the combination of CRLX101and olaparib.

Hair follicles will be collected at multiple time points: Pre-treatment on C1D1; Pre-treatment C1D3 (pre-olaparib); C1D4 (24hr after olaparib). At least 24 hours prior to the start of the study, the research nurse will contact Dr. Redon in Dr. Aladjem's lab (DTB-LMP/CCR/NCI, Bldg 37/ Rm 5056) to inform him when samples will be taken (Tel: 301-451-8576 (L); 301-760-6275 (Cell); redonc@mail.nih.gov). Dr. Redon will provide tubes for collecting the plucked hairs. The tubes contain ice cold PBS labeled with the date/time of sampling, the protocol, and the unique identifier. Dr. Redon will also provide forceps for plucking. Dr. Redon should be notified of when the samples should be picked up.

Single hairs are plucked from the scalp with forceps. Plucked hairs from eyebrows will be collected only if scalp hairs cannot be provided. The aim is to acquire 12 hairs that contain a full

intact follicle and sheath. All the hairs from a patient are placed in microfuge tubes containing cold PBS and stored on ice. Upon delivery in Dr. Aladjem's lab, hairs will be fixed with paraformaldehyde and analyzed under a dissection microscope to select those containing a full intact follicle and sheath. Plucked hairs will be fluorescently stained for γ-H2AX and images will recorded by using a confocal microscope.

Blood will be collected in a 6-ml green top tube at the following time points during cycle 1: Pre-treatment on C1D1; Pre-treatment C1D3 (pre-olaparib); C1D4 (24hr after olaparib). When the patient is scheduled, the Trepel lab will be contacted [by email to trepel@helix.nih.gov and Sunmin Lee lees@pop.nci.nih.gov]. Blood from heparinized syringe will be mixed with a 1:1 ratio of room temperature PBS and layered over a 1:1 blood-PBS/Ficoll ratio in a conical centrifuge tube. The conical centrifuge tube should be centrifuged 25 minutes at room temperature at approximately 1000 rpm. The cell layer resting above the Ficoll and containing the peripheral blood mononuclear cells (PBMCs) will be aspirated and transferred to a 15 ml conical tube for washing in 15 ml PBS. PBMCs will be fixed with paraformaldehyde, spun onto a microscope slide and stain for γH2AX detection. Images from γ-H2AX-stained PBMCs will be recorded by using a confocal microscope

5.1.2 SLFN11

Expression of the gene SLFN11 has been found to correlate with the activity of topoisomerase inhibitors in studies using the National Cancer Institute cell line panel (NCI60) and the Cancer Cell Line Encyclopedia (CCLE) [21, 22]. SLFN11 expression predicted sensitivity to DNA damaging chemotherapy including Top1 and Top2 inhibitors, alkylating agents, platinum derivatives, DNA synthesis and PARP inhibitors [21-23]. In experiments using cells with endogenously high and low SLFN11 expression and siRNA- and Crispr-mediated silencing, SLFN11 was found to be causative in determining cell cycle arrest and cell death in response to DNA damaging agents in cancer cells [23]. Data from the CCLE, the NCI60 and The Cancer Genome Atlas (TCGA) indicate a broad range of SLFN11 expression in lung cancers, raising the possibility that high SLFN11 expression might enrich for tumors that are more likely to respond to DNA damaging chemotherapy; conversely low SLFN11 expression may predict tumors that are likely resistant. Pre-treatment SLFN11 expression in tumor samples will be assessed (IHC and RNA) to assess in an exploratory manner, the potentially role of SLFN11 as a predictor of response to CRLX101 plus olaparib.

5.1.3 Tumor biopsies and genomic DNA

Biopsies will be done only in phase II part of the study and in SCLC patients in the phase I part of the study.

Paired tumor biopsies will be obtained by minimally invasive methods such as CT guided percutaneous biopsies before and after treatment [C1D4 (24hr after olaparib)] and at disease progression. The site of biopsy will be determined in discussion with interventional radiologist. If it can be safely obtained, 4 cores of tumor tissue will be collected. Two cores will be sent to pathology- one for confirmation of diagnosis and another will be used for making an FFPE block. The other 2 cores will be flash frozen at the time of biopsy. When the patient is scheduled, the Trepel lab will be contacted [by email to trepel@helix.nih.gov and Sunmin Lee lees@pop.nci.nih.gov]. Interventional Radiology will call the lab at 301-496-1547 when the

patient arrives in IR and a lab member will be present at Interventional Radiology for the procedure. The cores will be flash frozen, 2D barcoded, and stored in liquid nitrogen.

Formalin fixed paraffin embedded tumor tissue (FFPE) from the new biopsies and or from archived FFPE tissue from prior biopsies/surgical procedures will be sent to the Trepel lab as described above. Genomic DNA and RNA will be extracted from the tumor and archived for genomic analysis. Tumor tissue obtained before start of treatment will be used for the following assessments: γ-H2AX, SLFN11, and POLQ expression; post-treatment biopsy will be assessed for γ-H2AX expression.

5.1.4 Immune Subsets

Little is known of the immunomodulatory effects of DNA damage-inducing cytotoxic therapy. Peripheral blood mononuclear cells (PBMC) obtained before and during treatment [(Pre-treatment on C1D1; Pre-treatment C1D3 (pre-olaparib); C1D4 (24hr after olaparib); Pre-treatment C2D1)] will be assessed by the Trepel Lab in the Developmental Therapeutics Branch using multiparameter flow cytometry for immune subsets including but not necessarily limited to Tregs, myeloid-derived suppressor cells, effector and exhausted CD4+ and CD8+ T-cells, and CD14+ monocytes. Assessment will include functional markers, i.e. PD-1, Tim-3, CTLA-4, CD40, HLA-DR , and/or PD-L1. Members of the lab will procure the peripheral blood samples, enter the samples in a secure patient database, process the samples for viable cell storage, label each sample with a unique 2D barcode, and viably store the samples. They will prepare the samples for staining, stain and run the samples by multiparametric flow cytometry (MACSQuant, Miltenyi Biotec, Bergisch Gladbach, DE), the data will be analyzed by FlowJo v.X.0.6. Peripheral blood will be drawn into two 8-ml CPT citrate blue/black tubes for each time point. These assessments will be performed by the Trepel lab.

5.1.5 Circulating Tumor Cells

Circulating tumor cells (CTCs), which can be prevalent in SCLC, present a readily accessible 'liquid biopsy'. Peripheral blood will be collected [Pre-treatment on C1D1; Pre-treatment C2D1; at disease progression] to correlate circulating tumor cell (CTC) levels at baseline or levels pre- and post-therapy with clinical response and survival. CTCs will be assessed using ferrofluidic enrichment and multiparameter flow cytometric detection. CTCs will be identified as viable, nucleated cells, that positively express one or more epithelial or tumor markers and are negative for expression of hematopoietic markers. CTCs will be enumerated and if sufficient, additional characterization performed included RAD51 foci, gene expression by droplet digital PCR, NanoString, whole transcriptome by Illumina or another platform as appropriate to the sample. Peripheral blood will be drawn into two 10-ml lavender top tubes and one 2.5 ml PAXgene RNA tube for each time point. These assessments will be performed by the Trepel lab.

5.1.6 A multiplexed gene expression approach to profiling immune gene and DNA damage response signatures

Peripheral immune and DNA damage response transcriptional signatures will be evaluated by the Trepel Lab using the NanoString nCounter® platform (NanoString Technologies, Seattle, WA). We will use the nCounter 770 gene PanCancer Immune Profiling Panel and the newly released 170 gene DNA Damage & Repair panel, built in collaboration with the Developmental Therapeutics Branch. Peripheral blood will be collected in a PAXgene tube (PreAnalytix; 2.5 cc peripheral blood per tube) per the manufacturer’s instructions. RNA will be isolated using the

PAXgene Blood RNA Kit according to the manufacturer's instructions. The peripheral gene signatures will be evaluated at baseline and post-therapy to look for correlates of clinical response.

5.1.7 PK

Blood samples for the determination of CRLX101 and olaparib plasma levels will be obtained from participating patients via 6mL sodium heparin tube (BD, Franklin Lakes, NJ) per the table below. Samples will be obtained following the first dose on Cycle 1, day 1, and again on Cycle 6, day 1 in order to assess the extent of accumulation from repeated dosing at the timepoints specified below:

sample	cycle	day	time (approx)	hr post-CRLX101 start	post-olaparib
1	1	1	8:00a	0hr (predose)	-
2	1	1	8:30a	0.5hr (mid-infusion)	-
3	1	1	9:00a	1hr (end of infusion (EOI))	-
4	1	1	10:00a	2 hr (1 hr post EOI)	-
5	1	1	11:00a	3 hr (2 hr post EOI)	-
6	1	1	9:00p	13 hr (12 hr post EOI)	-
7	1	2	9:00a	25 hr (24 hr post EOI)	-
8	1	3	10:00a	50 hr(49 hr post EOI)	2 hr
9	1	15	8:00a	360hr (trough,just prior to Day15 dose)	
10	6	1	8:00a	0hr (predose)	-
11	6	1	8:30a	0.5hr (mid-infusion)	-
12	6	1	9:00a	1hr (end of infusion (EOI))	-
13	6	1	10:00a	2 hr (1 hr post EOI)	-
14	6	1	11:00a	3 hr (2 hr post EOI)	-
15	6	1	9:00p	13 hr (12 hr post EOI)	-
16	6	2	9:00a	25 hr (24 hr post EOI)	-
17	6	3	10:00a	50 hr(49 hr post EOI)	2 hr
18	6	15	8:00a	360hr (trough,just prior to Day15 dose)	

The PK samples will be placed immediately on wet ice and refrigerated. The date and exact time of each blood draw should be recorded on the sample tube and the PK sheet. Please page 102-11964 for immediate pick-up. Contact Dr. Figg's Clinical Pharmacology Program (Blood Processing Core) in Bldg 10/5A09 at 301-402-3622 or 301-594-6131 with any questions.

Bioanalytical measurements of CRLX101 and olaparib will be measured by individual ultra HPLC-MS/MS assays optimized for each drug by the Clinical Pharmacology Program (CPP). This data will be used to assess any drug-drug interactions are present between olaparib and CRLX101, as well as to correlate adverse events and efficacy to each drug's exposure. Neither olaparib nor camptothecin are extensively metabolized into pharmacologically active metabolites, therefore there are no relevant pharmacogenomic considerations regarding drug metabolizing enzymes or transporters with this combination.

5.2 Sample Storage, Tracking and Disposition

All samples [except PK samples- which will be sent to Doug Figg's lab; one core of research biopsy- which will be stored in the Laboratory of Pathology; hair samples which will be collected and processed by Dr. Redon, Developmental Therapeutics Branch] will be sent to and stored in Jane Trepel's Lab at NCI, Bldg 10, Bethesda, MD. Place all samples at room

temperature, phone the Trepel Lab at 301-496-1547 and a laboratory member will come to pick up the sample.

All samples will be ordered and tracked through the CRIS Screens. Should a CRIS screen not be available, the NIH form 2803-1 will be completed and will accompany the specimen and be filed in the medical record. Samples will not be sent outside NIH without IRB notification and an executed MTA. Any transfer of materials to other NIH or non-NIH investigators will occur following NIH Intramural Research Program guidelines. If the subject withdraws consent the participants data will be excluded from future distributions, but data that have already been distributed for approved research use will not be able to be retrieved.

5.2.1 Trepel Lab

Tracking and disposition of samples will conform to the NCI CCR Biospecimen Guidelines.

All samples will be barcoded and data entered and stored in the Labmatrix system utilized by the NIH Clinical Center. This is a secure system with access limited to defined personnel. All such personnel with access to subject information annually complete the NIH online Protection of Human Subjects course.

Labmatrix creates a unique barcode ID for every sample which cannot be traced back to subjects without Labmatrix access. The data recorded for each sample includes the subject ID, name, trial name/protocol number, date/time drawn, as well as box and freezer location. Subject demographics associated with the clinical center patient number are provided in the system. For each sample, there are notes associated with the processing method (delay in sample processing, storage conditions on the ward, etc.). Access to personally identifiable information (PII) is limited to the PI and associate investigators.

An additional layer of encryption will be added for samples undergoing genetic analysis in the Trepel lab where a separate clinically annotated unique sample ID will be generated linked with the sample ID in Labmatrix. As additional clinical information is generated and linked to the unique patient ID, it is also electronically linked via Labmatrix to the sample ID. The Trepel lab will proceed with sample analysis and record data under the unique sample ID.

Barcoded samples are stored in barcoded boxes in a locked freezer at either -20 or -80°C or in liquid nitrogen according to stability requirements. These freezers are located onsite, and access to stored clinical samples is restricted. Samples will be stored until requested by a researcher named on the protocol. All requests are monitored and tracked in the Labmatrix System. All researchers are required to sign a form stating that the samples are only to be used for research purposes associated with this trial (as per the IRB approved protocol) and that any unused samples must be returned to the NCI. It is the responsibility of the NCI Principal Investigator to ensure that the samples requested are being used in a manner consistent with IRB approval.

Samples will be stored in a freezer at either -4° C or -70° C behind a door locked after working hours. Samples will be tracked by a designated member of the laboratory who is responsible for notifying the PI about requests for use of the material, for allocating the material to other members of the laboratory, for recording the disposition of the allocated material.

5.2.2 Clinical Pharmacology Program (Figg Lab)

Upon arrival in the Clinical Pharmacology Program, samples will be centrifuged and the plasma transferred into cryovials for storage at -80 C until the time of analysis. In addition, samples will be barcoded.

All PK samples will be bar-coded, with data entered and stored in the Patient Sample Data Management System (PSDMS, aka Labrador) utilized by the CPP. This is a secure program, with access to PSDM System limited to defined CPP personnel, who are issued individual user accounts. The program creates a unique barcode ID for every sample and sample box, which cannot be traced back to patients with PSDMS access. The data recorded for each sample includes the patient ID, name, trial name/protocol number, time drawn, cycle time point, dose, material type, as well as box and freezer locations. Patient demographics associated with the clinical center patient number are provided in the system. For each sample, there are notes associated with the processing method (e.g. delay in sample processing, storage conditions on the ward, etc.).

Bar-coded samples are stored in bar-coded boxes in locked freezers at either -20 C or -80 C according to stability requirements. These freezers are located onsite in the CPP and offsite at NCI Frederick Central Repository Services (Fisher Bioservices) in Frederick, MD. Samples will be stored until requested by a researcher named on the protocol. All requests are monitored and tracked in PSDMS. All researchers are required to sign a form stating that the samples are only to be used for research purposes associated with this trial (as per IRB approved protocol) and that any unused samples must be returned to the CPP.

Following completion of this study, samples will remain in storage as detailed above. Access to these samples will only be granted following IRB approval of an additional protocol, granting the rights to use the material.

If, at any time, a patient withdraws from the study and does not wish for their existing samples to be utilized, the individual must provide a written request. Following receipt of this request, the samples will be destroyed (or returned to the patient, if so requested), and reported as such to the IRB. Any samples lost (in transit or by a researcher) or destroyed due to unknown sample integrity (i.e. broken freezer allows for extensive sample thawing, etc.) will be reported as such to the IRB.

Sample bar-codes are linked to patient demographics and limited clinical information. This information will only be provided to investigators listed on this protocol, via registered use of the PSDMS. It is critical that the sample remains linked to patient information such as race, age, dates of diagnosis and death, and histological information about the tumor, in order to correlate genotype with these variables.

5.2.3 Redon (Aladjem) Lab

At least 24 hours in advance, the research nurse will contact Dr. Redon in Dr. Aladjem's lab (DTB-LMP/CCR/NCI, Bldg 37/ Rm 5056) to inform him when samples will be taken (Tel: 301-451-8576 (L); 301-760-6275 (Cell); redonc@mail.nih.gov). Dr. Redon will provide tubes for collecting the plucked hairs which contain ice cold PBS, and are labeled with the date/time of sampling, the protocol, and the unique identifier. Dr. Redon should be notified of when the samples should be picked up.

All of the hairs from a patient will be placed in microfuge tubes containing cold PBS and stored on ice. Upon delivery in Dr. Aladjem's lab, hairs will be fixed with paraformaldehyde and analyzed under a dissection microscope to select those containing a full intact follicle and sheath. Plucked hairs will be fluorescently stained for γ-H2AX and images will recorded by using a confocal microscope.

5.2.4 Future Use/IRB Reporting/Protocol Completion/Sample Destruction

Blood and tissue specimens collected in the course of this research project may be banked and used in the future to investigate new scientific questions related to this study, including gene/protein expression and germline analysis. However, this research may only be done if the risks of the new questions were covered in the consent document and the proposed research has undergone prospective IRB review and approval. If new risks are associated with the research (e.g., analysis of germ line genetic mutations.) the Principal Investigator must amend the protocol and obtain informed consent from all research subjects.

Following completion of this study, samples will remain in storage as detailed above only for those subjects that agreed to future use in the Optional Studies section of the consent form. Access to these samples will only be granted following IRB approval of an additional protocol, granting the rights to use the material. Currently, there is no plan to use these samples outside of the use described in the protocol.

The PI will report destroyed samples to the IRB if samples become unsalvageable because of environmental factors (ex. broken freezer or lack of dry ice in a shipping container) or if a subject withdraws consent. If the subject withdraws consent the participants data will be excluded from future distributions, but data that have already been distributed for approved research use will not be able to be retrieved.

Samples will also be reported as lost if they are lost in transit between facilities or misplaced by a researcher. Freezer problems, lost samples or other problems associated with samples will also be reported to the IRB, the NCI Clinical Director, and the office of the CCR, NCI.

5.3 SAMPLES FOR GENETIC/GENOMIC ANALYSIS

5.3.1 Description of the scope of genetic/genomic analysis

One of the exploratory endpoints of this protocol is to characterize genetic alterations which predict response and changes associated with the development of chemoresistance. To this end, whole and targeted genome sequencing may be performed on banked tumor samples collected pre-treatment and/or post-progression. Since analysis of germline variants is essential to fully characterize the somatic mutations identified in genome sequencing, these assays will involve both somatic and germline DNA.

5.3.2 Certificate of Confidentiality

As part of study efforts to provide confidentiality of subject information, this study will obtain a Certificate of Confidentiality which helps to protect personally identifiable research information. The Certificate of Confidentiality allows investigators on this trial to refuse to disclose identifying information related to the research participants, should such disclosure have adverse consequences for subjects or damage their financial standing, employability, insurability or

reputation. The informed consent includes the appropriate coverage and restrictions of the Certificate of Confidentiality.

5.3.3 Management of Results

The analyses that we perform in our laboratory are for research purposes only; they are not nearly as sensitive as the tests that are performed in a laboratory that is certified to perform genetic testing. Changes that we observe unrelated to our research may or may not be valid. Therefore, we do not plan to inform participants of the results of testing on the tissue and blood that is performed in our research lab. However, in the unlikely event that a clinically actionable gene variant is discovered, , subjects will be contacted. Clinically actionable findings for the purpose of this study are defined as disorders appearing in the American College of Medical Genetics and Genomics recommendations for the return of incidental findings that is current at the time of primary analysis. (A list of current guidelines is maintained on the CCR intranet: https://ccrod.cancer.gov/confluence/display/CCRCRO/Incidental+Findings+Lists) Subjects who still remain on the study will be contacted at this time with a request to provide a blood sample to be sent to a CLIA certified laboratory. If the research findings are verified in the CLIA certified lab, the subject will be referred to the NCI CCR Genetics Branch for the disclosure of the results.

This is the only time during the course of the study that incidental findings will be returned. No interrogations regarding clinically actionable findings will be made after the primary analysis.

Note: Up to date contact information must be maintained on subjects to have their results returned. If a subject's participation in the study ends prior to the primary analysis of whole genome/exome sequencing studies, they should be enrolled on study 96-C-0071, a follow up protocol, in order to allow for post study contact for the dissemination of any incidental findings and the maintenance of contact information.

6 DATA COLLECTION AND EVALUATION

6.1 Data Collection

C3D and LabMatrix will be used for data collection.

The PI will be responsible for overseeing entry of data into an in-house password protected electronic system and ensuring data accuracy, consistency and timeliness. The principal investigator, associate investigators/research nurses and/or a contracted data manager will assist with the data management efforts. All human subjects personally identifiable information (PII) as defined in accordance to the Health Insurance Portability and Accountability Act, eligibility and consent verification will be recorded. Primary data obtained during the conduct of the protocol will be kept in secure network drives or in approved alternative sites that comply with NIH security standards. Primary and final analyzed data will have identifiers so that research data can be attributed to an individual human subject participant.

End of study procedures: Data will be stored according to HHS and FDA regulations as applicable.

Loss or destruction of data: Should we become aware that a major breech in our plan to protect subject confidentiality and trial data has occurred, the IRB will be notified.

6.2 DATA SHARING PLANS

6.2.1 Human Data Sharing Plan

What data will be shared?

I will share human data generated in this research for future research as follows (check all that apply):

_x__ De-identified data in an NIH-funded or approved public repository.

___ De-identified data in another public repository.

_x__ Identified data in BTRIS (automatic for activities in the Clinical Center)

__x_ De-identified or identified data with approved outside collaborators under appropriate agreements.

___ I will not share human data generated in this research for future research. If checked, explain:

How and where will the data be shared?

Data will be shared through (check all that apply):

_x__ An NIH-funded or approved public repository: clinicaltrials.gov; dbGaP.

___ Another public repository. Insert name or names: ______________________________.

_x__ BTRIS (automatic for activities in the Clinical Center)

_x__ Approved outside collaborators under appropriate individual agreements.

_x__ Publication and/or public presentations.

When will the data be shared?

_x__ Before publication.

__x_ At the time of publication or shortly thereafter.

6.2.2 Genomic Data Sharing Plan

Unlinked genomic data will be deposited in public genomic databases such as dbGaP in compliance with the NIH Genomic Data Sharing Policy.

6.3 RESPONSE CRITERIA

For the purposes of this study, patients should be re-evaluated for response every 2 cycles. In addition to a baseline scan, confirmatory scans should also be obtained _4_ weeks following initial documentation of objective response.

Response and progression will be evaluated in this study using the new international criteria proposed by the revised Response Evaluation Criteria in Solid Tumors (RECIST) guideline (version 1.1) [24]. Changes in the largest diameter (unidimensional measurement) of the tumor lesions and the shortest diameter in the case of malignant lymph nodes are used in the RECIST criteria.

6.3.1 Definitions

Evaluable for toxicity: All patients will be evaluable for toxicity from the time of their first treatment with CRLX101.

Evaluable for objective response: Only those patients who have measurable disease present at baseline, have received at least one cycle of therapy, and have had their disease re-evaluated will be considered evaluable for response. These patients will have their response classified according to the definitions stated below. (Note: Patients who exhibit objective disease progression prior to the end of cycle 1 will also be considered evaluable.)

Evaluable Non-Target Disease Response: Patients who have lesions present at baseline that are evaluable but do not meet the definitions of measurable disease, have received at least one cycle of therapy, and have had their disease re-evaluated will be considered evaluable for non-target disease. The response assessment is based on the presence, absence, or unequivocal progression of the lesions.

6.3.2 Disease Parameters

Measurable disease: Measurable lesions are defined as those that can be accurately measured in at least one dimension (longest diameter to be recorded) as ≥20 mm by chest x-ray, as ≥10 mm with CT scan, or ≥10 mm with calipers by clinical exam. All tumor measurements must be recorded in millimeters (or decimal fractions of centimeters).

Malignant lymph nodes. To be considered pathologically enlarged and measurable, a lymph node must be ≥15 mm in short axis when assessed by CT scan (CT scan slice thickness recommended to be no greater than 5 mm). At baseline and in follow-up, only the short axis will be measured and followed.

Non-measurable disease. All other lesions (or sites of disease), including small lesions (longest diameter <10 mm or pathological lymph nodes with ≥10 to <15 mm short axis), are considered non-measurable disease. Bone lesions, leptomeningeal disease, ascites, pleural/pericardial effusions, lymphangitis cutis/pulmonitis, inflammatory breast disease, and abdominal masses (not followed by CT or MRI), are considered as non-measurable.

Note: Cystic lesions that meet the criteria for radiographically defined simple cysts should not be considered as malignant lesions (neither measurable nor non-measurable) since they are, by definition, simple cysts.

'Cystic lesions' thought to represent cystic metastases can be considered as measurable lesions, if they meet the definition of measurability described above. However, if non-cystic lesions are present in the same patient, these are preferred for selection as target lesions.

Target lesions. All measurable lesions up to a maximum of 2 lesions per organ and 5 lesions in total, representative of all involved organs, should be identified as **target lesions** and recorded and measured at baseline. Target lesions should be selected on the basis of their size (lesions

with the longest diameter), be representative of all involved organs, but in addition should be those that lend themselves to reproducible repeated measurements. It may be the case that, on occasion, the largest lesion does not lend itself to reproducible measurement in which circumstance the next largest lesion which can be measured reproducibly should be selected. A sum of the diameters (longest for non-nodal lesions, short axis for nodal lesions) for all target lesions will be calculated and reported as the baseline sum diameters. If lymph nodes are to be included in the sum, then only the short axis is added into the sum. The baseline sum diameters will be used as reference to further characterize any objective tumor regression in the measurable dimension of the disease.

Non-target lesions. All other lesions (or sites of disease) including any measurable lesions over and above the 5 target lesions should be identified as **non-target lesions** and should also be recorded at baseline. Measurements of these lesions are not required, but the presence, absence, or in rare cases unequivocal progression of each should be noted throughout follow-up.

6.3.3 Methods for Evaluation of Measurable Disease

All measurements should be taken and recorded in metric notation using a ruler or calipers. All baseline evaluations should be performed as closely as possible to the beginning of treatment and never more than 4 weeks before the beginning of the treatment.

The same method of assessment and the same technique should be used to characterize each identified and reported lesion at baseline and during follow-up. Imaging-based evaluation is preferred to evaluation by clinical examination unless the lesion(s) being followed cannot be imaged but are assessable by clinical exam.

Clinical lesions: Clinical lesions will only be considered measurable when they are superficial (e.g., skin nodules and palpable lymph nodes) and ≥10 mm diameter as assessed using calipers (e.g., skin nodules). In the case of skin lesions, documentation by color photography, including a ruler to estimate the size of the lesion, is recommended.

Chest x-ray: Lesions on chest x-ray are acceptable as measurable lesions when they are clearly defined and surrounded by aerated lung. However, CT is preferable.

Conventional CT and MRI: This guideline has defined measurability of lesions on CT scan based on the assumption that CT slice thickness is 5 mm or less. If CT scans have slice thickness greater than 5 mm, the minimum size for a measurable lesion should be twice the slice thickness. MRI is also acceptable in certain situations (e.g. for body scans).

Use of MRI remains a complex issue. MRI has excellent contrast, spatial, and temporal resolution; however, there are many image acquisition variables involved in MRI, which greatly impact image quality, lesion conspicuity, and measurement. Furthermore, the availability of MRI is variable globally. As with CT, if an MRI is performed, the technical specifications of the scanning sequences used should be optimized for the evaluation of the type and site of disease. Furthermore, as with CT, the modality used at follow-up should be the same as was used at baseline and the lesions should be measured/assessed on the same pulse sequence. It is beyond

the scope of the RECIST guidelines to prescribe specific MRI pulse sequence parameters for all scanners, body parts, and diseases. Ideally, the same type of scanner should be used and the image acquisition protocol should be followed as closely as possible to prior scans. Body scans should be performed with breath-hold scanning techniques, if possible.

PET-CT: At present, the low dose or attenuation correction CT portion of a combined PET-CT is not always of optimal diagnostic CT quality for use with RECIST measurements. However, if the site can document that the CT performed as part of a PET-CT is of identical diagnostic quality to a diagnostic CT (with IV and oral contrast), then the CT portion of the PET-CT can be used for RECIST measurements and can be used interchangeably with conventional CT in accurately measuring cancer lesions over time. Note, however, that the PET portion of the CT introduces additional data which may bias an investigator if it is not routinely or serially performed.

Ultrasound: Ultrasound is not useful in assessment of lesion size and should not be used as a method of measurement. Ultrasound examinations cannot be reproduced in their entirety for independent review at a later date and, because they are operator dependent, it cannot be guaranteed that the same technique and measurements will be taken from one assessment to the next. If new lesions are identified by ultrasound in the course of the study, confirmation by CT or MRI is advised. If there is concern about radiation exposure at CT, MRI may be used instead of CT in selected instances.

Endoscopy, Laparoscopy: The utilization of these techniques for objective tumor evaluation is not advised. However, such techniques may be useful to confirm complete pathological response when biopsies are obtained or to determine relapse in trials where recurrence following complete response (CR) or surgical resection is an endpoint.

Tumor markers: Tumor markers alone cannot be used to assess response. If markers are initially above the upper normal limit, they must normalize for a patient to be considered in complete clinical response. Specific guidelines for both CA-125 response (in recurrent ovarian cancer) and PSA response (in recurrent prostate cancer) have been published [25-27]. In addition, the Gynecologic Cancer Intergroup has developed CA-125 progression criteria which are to be integrated with objective tumor assessment for use in first-line trials in ovarian cancer [28].

Cytology, Histology: These techniques can be used to differentiate between partial responses (PR) and complete responses (CR) in rare cases (e.g., residual lesions in tumor types, such as germ cell tumors, where known residual benign tumors can remain).

The cytological confirmation of the neoplastic origin of any effusion that appears or worsens during treatment when the measurable tumor has met criteria for response or stable disease is mandatory to differentiate between response or stable disease (an effusion may be a side effect of the treatment) and progressive disease.

FDG-PET: While FDG-PET response assessments need additional study, it is sometimes reasonable to incorporate the use of FDG-PET scanning to complement CT scanning in assessment of progression (particularly possible 'new' disease). New lesions on the basis of FDG-PET imaging can be identified according to the following algorithm:

a. Negative FDG-PET at baseline, with a positive FDG-PET at follow-up is a sign of PD based on a new lesion.
b. No FDG-PET at baseline and a positive FDG-PET at follow-up: If the positive FDG-PET at follow-up corresponds to a new site of disease confirmed by CT, this is PD. If the positive FDG-PET at follow-up is not confirmed as a new site of disease on CT, additional follow-up CT scans are needed to determine if there is truly progression occurring at that site (if so, the date of PD will be the date of the initial abnormal FDG-PET scan). If the positive FDG-PET at follow-up corresponds to a pre-existing site of disease on CT that is not progressing on the basis of the anatomic images, this is not PD.
c. FDG-PET may be used to upgrade a response to a CR in a manner similar to a biopsy in cases where a residual radiographic abnormality is thought to represent fibrosis or scarring. The use of FDG-PET in this circumstance should be prospectively described in the protocol and supported by disease-specific medical literature for the indication. However, it must be acknowledged that both approaches may lead to false positive CR due to limitations of FDG-PET and biopsy resolution/sensitivity.

Note: A ‘positive’ FDG-PET scan lesion means one which is FDG avid with an uptake greater than twice that of the surrounding tissue on the attenuation corrected image.

6.3.4 Response Criteria

6.3.4.1 Evaluation of Target Lesions

<u>Complete Response (CR)</u>: Disappearance of all target lesions. Any pathological lymph nodes (whether target or non-target) must have reduction in short axis to <10 mm.

<u>Partial Response (PR)</u>: At least a 30% decrease in the sum of the diameters of target lesions, taking as reference the baseline sum of diameters.

<u>Progressive Disease (PD)</u>: At least a 20% increase in the sum of the diameters of target lesions, taking as reference the smallest sum on study (this includes the baseline sum if that is the smallest on study). In addition to the relative increase of 20%, the sum must also demonstrate an absolute increase of at least 5 mm. (Note: the appearance of one or more new lesions is also considered progressions).

<u>Stable Disease (SD)</u>: Neither sufficient shrinkage to qualify for PR nor sufficient increase to qualify for PD, taking as reference the smallest sum of diameters while on study.

6.3.4.2 Evaluation of Non-Target Lesions

<u>Complete Response (CR)</u>: Disappearance of all non-target lesions and normalization of tumor marker level. All lymph nodes must be non-pathological in size (<10 mm short axis).

Note: If tumor markers are initially above the upper normal limit, they must normalize for a patient to be considered in complete clinical response.

<u>Non-CR/Non-PD:</u> Persistence of one or more non-target lesion(s) and/or maintenance of tumor marker level above the normal limits.

Progressive Disease (PD): Appearance of one or more new lesions and/or *unequivocal progression* of existing non-target lesions. *Unequivocal progression* should not normally trump target lesion status. It must be representative of overall disease status change, not a single lesion increase.

Although a clear progression of "non-target" lesions only is exceptional, the opinion of the treating physician should prevail in such circumstances, and the progression status should be confirmed at a later time by the review panel (or Principal Investigator).

6.3.4.3 Evaluation of Best Overall Response

The best overall response is the best response recorded from the start of the treatment until disease progression/recurrence (taking as reference for progressive disease the smallest measurements recorded since the treatment started). The patient's best response assignment will depend on the achievement of both measurement and confirmation criteria.

For Patients with Measurable Disease (i.e., Target Disease)

Target Lesions	Non-Target Lesions	New Lesions	Overall Response	Best Overall Response when Confirmation is Required*
CR	CR	No	CR	≥4 wks. Confirmation**
CR	Non-CR/Non-PD	No	PR	≥4 wks. Confirmation**
CR	Not evaluated	No	PR	
PR	Non-CR/Non-PD/not evaluated	No	PR	
SD	Non-CR/Non-PD/not evaluated	No	SD	Documented at least once ≥4 wks. from baseline**
PD	Any	Yes or No	PD	no prior SD, PR or CR
Any	PD***	Yes or No	PD	
Any	Any	Yes	PD	

*	See RECIST 1.1 manuscript for further details on what is evidence of a new lesion.
**	Only for non-randomized trials with response as primary endpoint.
***	In exceptional circumstances, unequivocal progression in non-target lesions may be accepted as disease progression.
Note:	Patients with a global deterioration of health status requiring discontinuation of treatment without objective evidence of disease progression at that time should be reported as "*symptomatic deterioration.*" Every effort should be made to document the objective progression even after discontinuation of treatment.

For Patients with Non-Measurable Disease (i.e., Non-Target Disease)

Non-Target Lesions	New Lesions	Overall Response
CR	No	CR
Non-CR/non-PD	No	Non-CR/non-PD*
Not all evaluated	No	not evaluated
Unequivocal PD	Yes or No	PD
Any	Yes	PD

* 'Non-CR/non-PD' is preferred over 'stable disease' for non-target disease since SD is increasingly used as an endpoint for assessment of efficacy in some trials so to assign this category when no lesions can be measured is not advised

6.3.5 Duration of Response

Duration of overall response: The duration of overall response is measured from the time measurement criteria are met for CR or PR (whichever is first recorded) until the first date that recurrent or progressive disease is objectively documented (taking as reference for progressive disease the smallest measurements recorded since the treatment started).

The duration of overall CR is measured from the time measurement criteria are first met for CR until the first date that progressive disease is objectively documented.

Duration of stable disease: Stable disease is measured from the start of the treatment until the criteria for progression are met, taking as reference the smallest measurements recorded since the treatment started, including the baseline measurements.

6.3.6 Progression-Free Survival

PFS is defined as the duration of time from start of treatment to time of progression or death, whichever occurs first.

6.3.7 Response Review

Tumor measurements will be performed in consultation with the Center for Cancer Research Radiology and Imaging Sciences image processing service.

6.4 TOXICITY CRITERIA

The following adverse event management guidelines are intended to ensure the safety of each patient while on the study. The descriptions and grading scales found in the revised NCI Common Terminology Criteria for Adverse Events (CTCAE) version 4.0 will be utilized for AE reporting. All appropriate treatment areas should have access to a copy of the CTCAE version 4.0. A copy of the CTCAE version 4.0 can be downloaded from the CTEP web site (http://ctep.cancer.gov/protocolDevelopment/electronic_applications/ctc.htm#ctc_40).

7 SAFETY REPORTING REQUIREMENTS/DATA AND SAFETY MONITORING PLAN

7.1 DEFINITIONS

7.1.1 Adverse Event

An adverse event is defined as any reaction, side effect, or untoward event that occurs during the course of the clinical trial associated with the use of a drug in humans, whether or not the event is considered related to the treatment or clinically significant. For this study, AEs will include events reported by the patient, as well as clinically significant abnormal findings on physical examination or laboratory evaluation. A new illness, symptom, sign or clinically significant laboratory abnormality or worsening of a pre-existing condition or abnormality is considered an AE. All AEs must be recorded on the AE case report form.

All AEs, including clinically significant abnormal findings on laboratory evaluations, regardless of severity, will be followed until return to baseline or stabilization of event. Serious adverse events that occur more than 30 days after the last administration of investigational agent/intervention and have an attribution of at least possibly related to the agent/intervention should be recorded and reported as per sections **7.2, 7.3, 7.4.**

An abnormal laboratory value will be considered an AE if the laboratory abnormality is characterized by any of the following:

- Results in discontinuation from the study
- Is associated with clinical signs or symptoms
- Requires treatment or any other therapeutic intervention
- Is associated with death or another serious adverse event, including hospitalization.
- Is judged by the Investigator to be of significant clinical impact
- If any abnormal laboratory result is considered clinically significant, the investigator will provide details about the action taken with respect to the test drug and about the patient's outcome.

7.1.2 Suspected adverse reaction

Suspected adverse reaction means any adverse event for which there is a reasonable possibility that the drug caused the adverse event. For the purposes of IND safety reporting, 'reasonable

possibility' means there is evidence to suggest a causal relationship between the drug and the adverse event. A suspected adverse reaction implies a lesser degree of certainty about causality than adverse reaction, which means any adverse event caused by a drug.

7.1.3 Unexpected adverse reaction

An adverse event or suspected adverse reaction is considered "unexpected" if it is not listed in the investigator brochure or is not listed at the specificity or severity that has been observed; or, if an investigator brochure is not required or available, is not consistent with the risk information described in the general investigational plan or elsewhere in the current application. "Unexpected" also refers to adverse events or suspected adverse reactions that are mentioned in the investigator brochure as occurring with a class of drugs or as anticipated from the pharmacological properties of the drug, but are not specifically mentioned as occurring with the particular drug under investigation.

7.1.4 Serious

An Unanticipated Problem or Protocol Deviation is serious if it meets the definition of a Serious Adverse Event or if it compromises the safety, welfare or rights of subjects or others.

7.1.5 Serious Adverse Event

An adverse event or suspected adverse reaction is considered serious if in the view of the investigator or the sponsor, it results in any of the following:

- Death,
- A life-threatening adverse drug experience
- Inpatient hospitalization or prolongation of existing hospitalization
- Persistent or significant incapacity or substantial disruption of the ability to conduct normal life functions
- A congenital anomaly/birth defect.
- Important medical events that may not result in death, be life-threatening, or require hospitalization may be considered a serious adverse drug experience when, based upon appropriate medical judgment, they may jeopardize the patient or subject and may require medical or surgical intervention to prevent one of the outcomes listed in this definition.

7.1.6 Disability

A substantial disruption of a person's ability to conduct normal life functions.

7.1.7 Life-threatening adverse drug experience

Any adverse event or suspected adverse reaction that places the patient or subject, in the view of the investigator or sponsor, at immediate risk of death from the reaction as it occurred, i.e., it does not include a reaction that had it occurred in a more severe form, might have caused death.

7.1.8 Protocol Deviation (NIH Definition)

Any change, divergence, or departure from the IRB-approved research protocol.

7.1.9 Non-compliance (NIH Definition)

The failure to comply with applicable NIH Human Research Protections Program (HRPP) policies, IRB requirements, or regulatory requirements for the protection of human research subjects.

7.1.10 Unanticipated Problem

Any incident, experience, or outcome that:

- Is unexpected in terms of nature, severity, or frequency in relation to

  (a) the research risks that are described in the IRB-approved research protocol and informed consent document; Investigator's Brochure or other study documents, and

  (b) the characteristics of the subject population being studied; **AND**

- Is related or possibly related to participation in the research; **AND**
- Suggests that the research places subjects or others at a *greater risk of harm* (including physical, psychological, economic, or social harm) than was previously known or recognized.

7.2 NCI-IRB Reporting

7.2.1 NCI-IRB Expedited Reporting of Unanticipated Problems and Deaths

The Protocol PI will report to the NCI-IRB:

- All deaths, except deaths due to progressive disease
- All Protocol Deviations
- All Unanticipated Problems
- All serious non-compliance

Reports must be received by the NCI-IRB within 7 working days of PI awareness via iRIS.

7.2.2 NCI-IRB Requirements for PI Reporting at Continuing Review

The protocol PI will report to the NCI-IRB:

1. A summary of all protocol deviations in a tabular format to include the date the deviation occurred, a brief description of the deviation and any corrective action.
2. A summary of any instances of non-compliance
3. A tabular summary of the following adverse events:
   - All Grade 2 **unexpected** events that are possibly, probably or definitely related to the research;
   - All Grade 3 and 4 events that are possibly, probably or definitely related to the research;
   - All Grade 5 events regardless of attribution;
   - All Serious Events regardless of attribution.

NOTE: Grade 1 events are not required to be reported.

7.2.3 NCI-IRB Reporting of IND Safety Reports

Only IND Safety Reports that meet the definition of an unanticipated problem will need to be reported to the NCI IRB.

7.3 IND Sponsor Reporting Criteria

An investigator must **immediately** report to the sponsor, using the mandatory MedWatch form 3500a, any serious adverse event, whether or not considered drug related, including those listed in the protocol or investigator brochure and must include an assessment of whether there is a reasonable possibility that the drug caused the event.

Study endpoints that are serious adverse events (e.g. all-cause mortality) must be reported in accordance with the protocol unless there is evidence suggesting a causal relationship between the drug and the event (e.g. death from anaphylaxis). In that case, the investigator must immediately report the death to the sponsor.

Events will be submitted to Dr. William Dahut, authorized representative for the IND Sponsor (CCR) at:

William Dahut, M.D.

Bldg 10, Room 3-2571 MSC 1206

Bethesda, MD 20892

Telephone: 301-435-8183

William.Dahut@nih.gov

Copy all MedWatch forms to: nciprotocolsupportoffice@mail.nih.gov

7.4 FDA Reporting Criteria

7.4.1 IND Safety Reports to the FDA (Refer to 21 CFR 312.32)

7.4.1.1 The Sponsor will notify the FDA of any unexpected fatal or life-threatening suspected adverse reactions as soon as possible but no later than 7 calendar days of initial receipt of the information using the MedWatch Form 3500a.

The Sponsor is also responsible for reporting any:

- suspected adverse reaction that is both serious and unexpected
- any findings from clinical, epidemiological, or pooled analysis of multiple studies or any findings from animal or in vitro testing that suggest a significant risk in humans exposed to the drug
- clinically important increase in the rate of a serious suspected adverse reaction over that listed in the protocol or investigator brochure

to the FDA and to all investigators no later than 15 calendar days after determining that the information qualifies for reporting using the MedWatch Form 3500a. If FDA requests any

additional data or information, the sponsor must submit it to the FDA as soon as possible, but no later than 15 calendars days after receiving the request.

7.4.2 FDA Annual Reports (Refer to 21 CFR 312.33)

The study Sponsor will submit a brief report annually of the progress of the trial within 60 days of the anniversary date that the IND went into effect as indicated in 21CFR 312.33, and any associated FDA correspondences regarding the IND annual report.

7.4.3 Expedited Adverse Event Reporting Criteria to the IND Manufacturer

7.4.3.1 Cerulean Pharma, Inc. Reporting Requirements

To ensure patient safety, every SAE, regardless of suspected causality, occurring after the patient begins taking study drug and until 30 days after the patient has stopped study treatment must be reported to Cerulean via INC Research (Cerulean's PV CRO) within 24 hours of learning of its occurrence. The SAE form located in the Study Specific Safety Management Plan (SMP) can be printed and used for this purpose. If the event is both a serious SAR and unexpected, the MedWatch 3500A form will be used.

Any SAE experienced after this 30 day period should only be reported to Cerulean / INC Research if the investigator suspects a causal relationship to the study drug. Recurrent episodes, complications, or progression of the initial SAE must be reported as follow-up to the original episode within 24 hours of the investigator receiving the follow-up information. An SAE occurring at a different time interval or otherwise considered completely unrelated to a previously reported one should be reported separately as a new event. The investigator must assess and record the relationship of each SAE to each specific study drug (if there is more than one study drug).

Any patient hypersensitivity/allergic reactions a patient experiences associated with CRLX101 must be reported to Cerulean / INC Research within 24 hours of learning of its occurrence.

SAE and important medical events, including any hypersensitivity/allergic reactions will be sent to Cerulean by email to:

PV@Ceruleanrx.com

Or alternatively by fax to:

1-617-494-1544

A summary report of other AEs and SARs will also be sent to Cerulean at a frequency no less than once a year and in a format agreed upon at Study Start-up. Instructions on sending these additional reports to Cerulean will be agreed upon with Cerulean clinical team members at Study Start-up.

7.4.3.2 AZD Reporting Requirements

The Sponsor shall report any adverse events or adverse reactions (each as defined in the protocol) that arise in relation to the study to (i) the relevant regulatory authorities in accordance with the applicable laws; and (ii) any overseeing ethics committee in accordance with its policies.

- The Sponsor is required to notify the Company of all Suspected Unexpected Serious Adverse Reactions (SUSARs) subject to expedited reporting under the Applicable Laws at the same time that the reports are sent to the FDA using a medwatch 3500A form.

- The Sponsor is required to notify the company within 30 days of all other SAEs [non-expedited reports], plus any AE reports relating to topics of special interest [MDS/AML, new primary malignancy and pneumonitis] using a medwatch 3500A form.

- The Sponsor shall provide the Company with line listing of all adverse events reported in the study. These line listings will be sent when the Sponsor receives an e-mail from Company.

- The Sponsor will inform the Company of any other matter relating to safety, quality or efficacy of the Company's medicinal product or which might affect the conduct of the study or the safety of subjects as soon as possible and at least in parallel with correspondence to regulators, IECs/IRBs and investigators.

- The Sponsor shall provide the Company all non-adverse events (AEs and SAEs) on the Final Study Report upon completion of the Study.

- The Sponsor shall respond to any query from the Company relating to SAEs, as part of the Company's pharmacovigilance processes.

Reports described above should be sent to AEMailboxClinicalTrialTCS@astrazeneca.com. Tata Consultancy Services (TCS) will be responsible for processing all SAEs onto the AZ Patient Safety Database. Reports can also be sent via FAX to 1-302-886-4114.

Non-serious adverse events and SAEs will be collected from the time consent is given, throughout the treatment period and up to and including the *30 day follow-up* period. After withdrawal from treatment, subjects must be followed-up for all existing and new AEs for *30 calendar days after the last dose of trial drug and/or until event resolution.* All new AEs occurring during that period must be recorded (if SAEs, then they must be reported to the FDA and AstraZeneca). All study-related toxicities/ SAEs must be followed until resolution, unless in the Investigator's opinion, the condition is unlikely to resolve due to the patient's underlying disease.

7.5 DATA AND SAFETY MONITORING PLAN

7.5.1 Principal Investigator/Research Team

The clinical research team will meet on a regular basis when patients are being actively treated on the trial to discuss each patient. Decisions about dose level enrollment and dose escalation if applicable will be made based on the toxicity data from prior patients.

All data will be collected in a timely manner and reviewed by the principal investigator or a lead associate investigator. Adverse events will be reported as required above. Any safety concerns, new information that might affect either the ethical and or scientific conduct of the trial, or protocol deviations will be immediately reported to the IRB using iRIS and to the Sponsor.

The principal investigator will review adverse event and response data on each patient to ensure safety and data accuracy. The principal investigator will personally conduct or supervise the investigation and provide appropriate delegation of responsibilities to other members of the research staff.

7.5.2 Sponsor Monitoring Plan

This trial will be monitored by personnel employed by Harris Technical Services on contract to the NCI, NIH. Monitors are qualified by training and experience to monitor the progress of clinical trials. Personnel monitoring this study will not be affiliated in any way with the trial conduct.

At least 25% of enrolled patients will be randomly selected and monitored at least biannually or as needed, based on accrual rate. The patients selected will have 100% source document verification done. Additional monitoring activities will include: adherence to protocol specified study eligibility, treatment plans, data collection for safety and efficacy, reporting and time frames of adverse events to the NCI IRB and FDA, and informed consent requirements. Written reports will be generated in response to the monitoring activities and submitted to the Principal investigator and Clinical Director or Deputy Clinical Director, CCR, NCI.

8 STATISTICAL CONSIDERATIONS

- Phase I: To determine the MTD/ recommended Phase 2 dose (RP2D) of CRLX101 in combination with olaparib in patients with refractory cancers
- Phase II: To determine the antitumor activity of olaparib plus CRLX101 with respect to progression free survival at 16 weeks separately in SCLC patients with resistant and sensitive relapse

For the phase I portion of the trial, a standard 3+3 design will be used, to determine the MTD of CRLX101 in combination with olaparib. The theoretical maximum number of subjects required to determine the MTD in the phase 1 portion of the study is 30 subjects (6 per dose level), although it is expected that as few as 15 subjects in 4 dose levels would be required to reach an MTD.

For the phase II portion, patients will be enrolled in two different cohorts based on sensitivity to initial chemotherapy: sensitive (S; patients with tumor progression that occurs 90 days or more after the last day of initial chemotherapy) and resistant/refractory (RR; patients with tumor progression that occurs less than 90 days after the last day of initial chemotherapy and patients with tumor progression during the initial chemotherapy or did not respond to initial chemotherapy). Based on data derived from a randomized trial in previously treated patients, the median PFS of sensitive and resistant/refractory patients to topotecan, (the currently approved therapy in this setting) are approximately 16 weeks and 12 weeks respectively [18]. Since there will not be a tumor assessment at 12 weeks on the present trial, both cohorts will have their primary efficacy determination take place at 16 weeks.

For the sensitive cohort, the goal will be determine if slightly more than 50% of patients may be identified as being without progression by 16 weeks; for the resistant/refractory cohort, the goal will be to determine if slightly more than 50% of patients may be identified as being without

progression by 12 weeks. In the latter case, assuming an exponential failure distribution, this is equivalent to determining if slightly more than 40% of the patients in the resistant/refractory cohort will be identified as being without progression by 16 weeks. In each cohort, this will be primarily estimated as a binomial fraction of the patients who are progression free at those time points from among those who are potentially followed for at least that long. A Kaplan-Meier curve of progression-free survival (PFS) will also be constructed for each cohort.

Thus, for the sensitive cohort, to see at least minimal potential improvement beyond 50% without progression at 16 weeks, the goal would be to determine if the combination would rule out a 40% cohort-specific progression free rate and target a rate of 65% cohort-specific progression free rate. For the resistant/refractory cohort, to see at least minimal potential improvement beyond 40% without progression at 16 weeks, the goal would be to determine if the combination would rule out a 30% cohort-specific progression free rate and target a rate of 55% cohort-specific progression free rate. In both cohorts, the percentage of patients who are progression free at 16 weeks will be considered ‘successes’ for this trial.

For the sensitive patients, the phase II trial will be conducted using an optimal two-stage phase II trial in order to rule out an unacceptably low success rate of 40% (p0=0.40) in favor of an improved success rate of 65% (p1=0.65). With alpha=0.10 (probability of accepting a poor treatment=0.10) and beta = 0.20 (probability of rejecting a good treatment=0.20), this first stage will enroll 11 evaluable patients, and if 0 to 5 of the 11 have a success (that is, 0 to 5 make it to 16 weeks without progression), then no further patients will be accrued. If 6 or more of the first 11 patients have success, then accrual would continue until a total of 20 evaluable sensitive patients have been enrolled.

As it may take up to 4 months to determine if a patient has experienced a success at 16 weeks, a temporary pause in the accrual may be necessary to ensure that enrollment to the second stage is warranted. If there are 6 to 10 patients with a success out of 20 patients, this would be an uninterestingly low success rate. If there were 11 or more of 20 (55%) who experienced a success, this would be sufficiently interesting to warrant further study in later trials. Under the null hypothesis (40% success rate), the probability of early termination is 75%.

For the resistant/refractory patients, the phase II trial will be conducted using an optimal two-stage phase II trial in order to rule out an unacceptably low success rate of 30% (p0=0.30) in favor of an improved success rate of 55% (p1=0.55). With alpha=0.10 (probability of accepting a poor treatment=0.10) and beta = 0.20 (probability of rejecting a good treatment=0.20), this first stage will enroll 8 evaluable patients, and if 0 to 2 of the 8 have a success (that is, 0 to 2 make it to 16 weeks without progression), then no further patients will be accrued. If 3 or more of the first 8 patients have success, then accrual would continue until a total of 20 evaluable resistant/refractory patients have been enrolled. As it may take up to 4 months to determine if a patient has experienced a success at 16 weeks, a temporary pause in the accrual may be necessary to ensure that enrollment to the second stage is warranted. If there are 3 to 8 patients with a success out of 20 patients, this would be an uninterestingly low success rate. If there were 9 or more of 20 (45%) who experienced a success, this would be sufficiently interesting to warrant further study in later trials. Under the null hypothesis (30% success rate), the probability of early termination is 55%.

Patients who receive at least one dose of the study drug will be evaluable for safety. Patients who complete at least one cycle of treatment and have a follow up imaging study will be evaluable for efficacy. The maximum number of patients on the phase I portion of the trial is 30 and the two cohorts in phase II may accrue up to 20 evaluable patients apiece. Thus, the maximum number of evaluable patients which may enroll on this trial is 70. In order to allow for a small number of in-evaluable patients, the accrual ceiling will be set at 75. It is anticipated that approximately 1 to 2 patients per month may enroll onto this trial; the trial is expect to complete accrual in 3-4 years.

9 COLLABORATIVE AGREEMENTS

9.1 Cooperative Research and Development Agreement (CRADA)

9.1.1 Cereulean Pharmaceuticals

The investigational study agent, CRLX101 is provided by the company, Cerulean Pharmaceuticals under a Collaborative Agreement [Cooperative Research and Development Agreement (CRADA).

9.1.2 Astra Zeneca

The investigational study agent, olaparib is provided by the company, Astra Zeneca under a Collaborative Agreement [Cooperative Research and Development Agreement (CRADA).

10 HUMAN SUBJECTS PROTECTIONS

10.1 Rationale For Subject Selection

As previously described, the subjects for this study will include all subjects who meet the eligibility criteria outlined in section **2.1**. No gender, racial, or ethnic groups will be excluded from participation in this trial.

10.2 Participation of Children

Because no dosing adverse event data are currently available on the use of CRLX101 in combination with olaparib in subjects <18 years of age, children are excluded from this study.

10.3 Evaluation of Benefits and Risks/Discomforts

10.3.1 Risks

10.3.1.1 Study drug risks

The risks associated with the specific study agents are described in Sections **11.1** and **11.2**. Subjects will be adequately monitored for the occurrence of any possible side effects.

10.3.1.2 Biopsy risks

The risks associated with biopsies are pain and bleeding at the biopsy site. In order to minimize pain, local anesthesia will be used. Rarely, there is a risk of infection at the sampling site. CT guidance may be used in obtaining biopsies. If so, there will also be a risk of exposure to radiation from up to 3 CT scans. This radiation exposure is not required for medical care and is for research purposes only. The amount of radiation received in this study is 0.54 rem which is below the guideline of 5 rem per year allowed for research subjects by the NIH Radiation Safety Committee.

10.3.1.3 Risks related to blood sampling

Side effects of blood draws include pain and bruising, lightheadedness, and rarely, fainting.

10.3.1.4 Risk related to hair collection

The only risk associate with hair collection is pain.

10.3.2 Benefits

The benefits include a possible decrease in the size of the tumor and the scientific knowledge that could be acquired through this trial.

10.4 Consent and Assent Process and Documentation

Subjects referred for the study will discuss the informed consent at the initial evaluation. Enrolled subjects will sign an informed consent form prior to any evaluation for the study. The principal investigator or an associate investigator on the trial will be obtaining consent after adequate explanation of the aims, methods, objectives and potential hazards of the study. It will be explained to the subjects that they are completely free to refuse to enter the study or to withdraw from it at any time for any reason.

Reconsent on this study may be obtained via telephone according to the following procedure: the informed consent document will be sent to the subject. An explanation of the study will be provided over the telephone highlighting the changes after the subject has had the opportunity to read the consent form. The subject will sign and date the informed consent. A witness to the patient signature--*someone other than the health care provider*--will sign and date the consent. The original informed consent document will be mailed, via the US Postal Service or FedEx, back to the consenting investigator who will sign and date the consent form with the date the consent was obtained via telephone. A fully executed copy will be returned via mail for the subject’s records. The informed consent process will be documented on a progress note by the consenting investigator and a copy of the informed consent document and note will be kept in the subject’s research record.

10.4.1 Informed consent of non-English speaking subjects

We anticipate the enrollment of Spanish speaking research participants into our study. The IRB approved full consent document will be translated into that language in accordance with the Clinical MAS Policy M77-2.

If there is an unexpected enrollment of a research participant for whom there is no translated extant IRB approved consent document, the principal investigator and/or those authorized to obtain informed consent will use the Short Form Oral Consent Process as described in MAS Policy M77-2, OSHRP SOP 12, 45 CFR 46.117 (b) (2), (*If a study with an IND or IDE, also cite 21 CFR 50.27 (b) (2)*). The summary that will be used is the English version of the extant IRB approved consent document. Signed copies of both the English version of the consent and the translated short form will be given to the subject or their legally authorized representative and the signed original will be filed in the medical record.

Unless the PI is fluent in the prospective subject’s language, an interpreter will be present to facilitate the conversation. Preferably someone who is independent of the subject (i.e., not a

family member) will assist in presenting information and obtaining consent. Whenever possible, interpreters will be provided copies of the relevant consent documents well before the consent conversation with the subject (24 to 48 hours if possible).

We request prospective IRB approval of the use of the short form process and will notify the IRB at the time of continuing review of the frequency of the use of the Short Form.

11 PHARMACEUTICAL INFORMATION

11.1 CRLX101-212 (IND#)

11.1.1 Description

CRLX101-212 is a polymer drug conjugate composed of 20 (S)-camptothecin conjugated to a biocompatible polymer. The compound self assembles into soluble nanoparticles composed of several molecules when dissolved in aqueous solution. Camptothecin (CPT) is an antineoplastic that inhibits topoisomerase I resulting in cell death during the S phase of the cell cycle. CRLX101-212 nanoparticles are believed to be taken up by tumor cells followed by release of the active pharmaceutical ingredient 20(S)-camptothecin.

11.1.2 Supplier/How Supplied

The CRLX101-212 is provided by the company, Cerulean Pharma Inc., the manufacturer of the drug under a Collaborative Agreement [Cooperative Research and Development Agreement (CRADA)].

CRLX101 will be provided at no cost to the study patient by It is supplied as a lyophilized cake in two configurations: a 30 mL single-use vial containing 35 mg of CPT equivalents (approximately 350 mg of polymer drug conjugate) and a 30 mL single-use vial containing 50 mg of CPT equivalents (approximately 500 mg of polymer drug conjugate).

11.1.3 Handling and Dispensing

CRLX101 is a sterile injectable and must be handled under appropriate controls, conditions, and aseptic techniques to maintain product sterility. CRLX101-212, a cytotoxic, should be prepared in a class II biological safety cabinet using standard precautions for the safe handling of antineoplastic agents. Latex gloves are recommended. It must be dispensed only from official study sites by authorized personnel according to local regulations, and stored in a secure area according to local regulations. It is the responsibility of the Investigator to ensure that study drug is only dispensed to eligible study patients.

11.1.4 Preparation

Vials of CRLX101-212 should be removed from the refrigerator and warmed to room temperature for approximately one hour prior to preparation. Concentrated CRLX101-212 solution is prepared by adding 20 mL of USP Sterile Water for Injection (SWFI) to each 50 mg vial or 14 mL of SWFI to each 35 mg vial. The required volume of SWFI is to be aseptically withdrawn into a sterile calibrated syringe and the syringe needle inserted through the vial

septum. The SWFI should be slowly added along the inside of the vial wall and not directly onto the lyophilized product cake to minimize foaming. The product is dissolved by gentle swirling (do not shake) until a clear homogenous solution is achieved. The majority of the product cake will dissolve quickly, however complete dissolution to a clear, homogenous solution will take longer and may require up to 30 minutes. The CRLX101-212 concentrated solution should be inspected visually for particulate matter prior to further preparation. Visually inspect for completion of reconstitution every 2-3 minutes. Reconstitution is complete when (i) foam head has dissipated down to a thin bubble ring along inner wall of vial, (ii) solids in solution and foam head are no longer visible, and (iii) translucent polymer "vapors" are no longer visible in solution and the solution is clear and uniform in appearance. Complete reconstitution of the vial may take up to 30 minutes.

Each milliliter of concentrated solution contains 2.5 mg of camptothecin equivalents. Concentrated CRLX101-212 solution is diluted to the recommended dose with 5% dextrose in water for injection (D5W) to a total volume of 500 mL. After determining the amount of CRLX101-212 concentrated solution required for dosing, an equivalent volume is aseptically withdrawn from a 500 mL infusion bag or bottle of D5W with a calibrated syringe. The measured volume of concentrated CRLX101-212 solution is then withdrawn with a calibrated syringe and injected into the prepared 500 mL D5W infusion bag or bottle. The solution is thoroughly mixed by gentle manual rotation.

11.1.5 Storage and Stability

CRLX101-212 vials should be stored at refrigerated conditions (2° - 8°C) and should only be accessible to authorized individuals. CRLX101-212 is stable for at least 3 years when stored at refrigerated conditions (2° - 8°C). The reconstituted solution is stable for 24 hours when stored at room temperature. Any unused product solution from the reconstituted vials should be destroyed per the site's standard operating procedures.

Formulated CRLX101-212 (i.e., IV bag) should be used within 6 hours of reconstituting the drug product vials.

11.1.6 Dosage and Administration

See Sections **3.2** and **3.3** for the dose levels of CRLX101-212 to be evaluated in this study. Each dose should be administered by IV infusion over 60 minutes on Day 1 (D1) of weeks 1, 3 and 5. See Section **11.1.4** for how to prepare each dose for administration. The first ~40mL will be administered over 10 minutes, and then the infusion rate will be increased to allow for complete administration over 60 minutes.

See Section **3.3.4** for management of any CRLX101-212 infusion related/hypersensitivity reactions.

The second and subsequent dose of CRLX101-212 may be delayed if related AEs have not resolved to grade 1 or better. If a dose of CRLX101-212 is delayed, then the subsequent dose should be administered 2 weeks later to avoid significant carry-over of unconjugated plasma CPT from one dose to the next (see Section **3.3**). However the maximum delay for AEs to resolve is 21 days beyond which the patient will be taken off treatment.

11.1.7 Premedication and Hydration

The premedication prior to CRLX101 treatment and pre and post hydration and additional prevention measurements to prevent hypersensitivity/infusion reaction have been described in Section **3.2.1**.

11.1.8 Return and Retention of CRLX101-212

Any used study drug vials, or partially used vials, remaining after trial is complete will be destroyed per institution drug destruction policy. Unused study drug vials may be destroyed in the same manner or returned if requested to Cerulean Pharma Inc.

11.2 OLAPARIB (AZD2281; LYNPARZA)

(Please refer to the Investigator's Brochure and the package insert for further details)

11.2.1 Source

The olaparib is provided by the company, Astra Zeneca under a Collaborative Agreement [Cooperative Research and Development Agreement (CRADA)].

Olaparib (Lynparza, AstraZeneca Pharmaceuticals LP) has been commercially Food and Drug Administration approved as monotherapy for the treatment of patients with deleterious or suspected deleterious germline BRCA mutated (gBRCAm) (as detected by an FDA-approved test) advanced ovarian cancer who have been treated with three or more prior lines of chemotherapy.

As of 20 March 2015, >3800 patients with ovarian, breast, pancreatic, gastric and a variety of other solid tumors are estimated to have received treatment with olaparib in clinical studies as either monotherapy or in combination with other chemotherapy/anti-cancer agents. Most new clinical studies are investigating the tablet formulation which delivers the therapeutic dose of olaparib in fewer dose units than the capsule.

The AstraZeneca Pharmaceutical Development R&D Supply Chain will supply olaparib or placebo to the investigator as round or oval *green film coated tablets.*

11.2.2 Pre-clinical experience

The pre-clinical experience is fully described in the current version of the olaparib Investigator's Brochure (IB).

11.2.3 Toxicology and safety pharmacology summary

Olaparib has been tested in a standard range of safety pharmacology studies e.g. dog cardiovascular and respiratory function tests, and the rat Irwin test. There were no noticeable effects on the cardiovascular or respiratory parameters in the anaesthetized dog or any behavioural, autonomic or motor effects in the rat at the doses studied.

The toxicology studies indicate that the target organ of toxicity is the bone marrow.

Further information can be found in the current version of the olaparib Investigator's Brochure

11.2.4 Approved indications and clinical experience; ; capsule formulation

Clinical experience with olaparib is fully described in the current version of the olaparib Investigator's Brochure.

11.2.5 Indications and Usage

Lynparza is a poly (ADP-ribose) polymerase (PARP) inhibitor indicated as monotherapy in patients with deleterious or suspected deleterious germline BRCA mutated (as detected by an FDA-approved test) advanced ovarian cancer who have been treated with three or more prior lines of chemotherapy.

The indication is approved under accelerated approval based on objective response rate and duration of response. Continued approval for this indication may be contingent upon verification and description of clinical benefit in confirmatory trials.

11.2.6 Dosage and Administration

Please refer to section **3.2.2** for administration instructions. Dose will be according to assigned dose level (see section **3.1)**.

11.2.7 Tablet Dosage Forms and Strengths

Olaparib is presented for oral administration as a green, film-coated tablet containing 25 mg, 100 mg, 150 mg or 200 mg of drug substance.

11.2.8 Warnings and Precautions

• Myelodysplastic syndrome/Acute Myeloid Leukemia: (MDS/AML) occurred in patients exposed to Lynparza, and some cases were fatal. Monitor patients for hematological toxicity at baseline and monthly thereafter. Discontinue if MDS/AML is confirmed.

• Pneumonitis: occurred in patients exposed to Lynparza, and some cases were fatal. Interrupt treatment if pneumonitis is suspected. Discontinue if pneumonitis is confirmed.

• Embryo-Fetal toxicity: Lynparza can cause fetal harm. Advise females of reproductive potential of the potential risk to a fetus and to avoid pregnancy.

11.2.9 Adverse Reactions

• Most common adverse reactions (≥20%) in clinical trials were anemia, nausea, fatigue (including asthenia), vomiting, diarrhea, dysgeusia, dyspepsia, headache, decreased appetite, nasopharyngitis/pharyngitis/URI, cough, arthralgia/musculoskeletal pain, myalgia, back pain, dermatitis/rash and abdominal pain/discomfort.

• Most common laboratory abnormalities (≥25%) were increase in creatinine, mean corpuscular volume elevation, decrease in hemoglobin, decrease in lymphocytes, decrease in absolute neutrophil count, and decrease in platelets.

11.2.10 Drug Interactions

• CYP3A Inhibitors: Avoid concomitant use of strong and moderate CYP3A inhibitors. If the inhibitor cannot be avoided, reduce the dose.

• CYP3A Inducers: Avoid concomitant use of strong and moderate CYP3A inducers. If a moderate CYP3A inducer cannot be avoided, be aware of a potential for decreased efficacy.

12 REFERENCES

1. Murai, J., et al., *Rationale for poly(ADP-ribose) polymerase (PARP) inhibitors in combination therapy with camptothecins or temozolomide based on PARP trapping versus catalytic inhibition.* J Pharmacol Exp Ther, 2014. **349**(3): p. 408-16.

2. van Meerbeeck, J.P., D.A. Fennell, and D.K. De Ruysscher, *Small-cell lung cancer.* Lancet, 2011. **378**(9804): p. 1741-55.

3. Ardizzoni, A., M. Tiseo, and L. Boni, *Validation of standard definition of sensitive versus refractory relapsed small cell lung cancer: a pooled analysis of topotecan second-line trials.* Eur J Cancer, 2014. **50**(13): p. 2211-8.

4. Gibson, B.A. and W.L. Kraus, *New insights into the molecular and cellular functions of poly(ADP-ribose) and PARPs.* Nature Reviews Molecular Cell Biology, 2012. **13**(7): p. 411-424.

5. Byers, L.A., et al., *Proteomic Profiling Identifies Dysregulated Pathways in Small Cell Lung Cancer and Novel Therapeutic Targets Including PARP1.* Cancer Discovery, 2012. **2**(9): p. 798-811.

6. Owonikoko, T.K., et al., *Poly (ADP) ribose polymerase enzyme inhibitor, veliparib, potentiates chemotherapy and radiation in vitro and in vivo in small cell lung cancer.* Cancer Med, 2014. **3**(6): p. 1579-94.

7. Cardnell, R.J., et al., *Proteomic markers of DNA repair and PI3K pathway activation predict response to the PARP inhibitor BMN 673 in small cell lung cancer.* Clin Cancer Res, 2013. **19**(22): p. 6322-8.

8. Y. Feng, R.C., L.A. Byers, B. Wang, Y. Shen. , *Talazoparib (BMN 673) as single agent and in combination with temozolomide or PI3K pathway inhibitors in small cell lung cancer and gastric cancer models.* . 26th EORTC-NCI-AACR Symposium on Molecular Targets and Cancer Therapeutics (abstract) 2014.

9. Delaney, C.A., et al., *Potentiation of temozolomide and topotecan growth inhibition and cytotoxicity by novel poly(adenosine diphosphoribose) polymerase inhibitors in a panel of human tumor cell lines.* Clin Cancer Res, 2000. **6**(7): p. 2860-7.

10. Ihnen, M., et al., *Therapeutic potential of the poly(ADP-ribose) polymerase inhibitor rucaparib for the treatment of sporadic human ovarian cancer.* Mol Cancer Ther, 2013. **12**(6): p. 1002-15.

11. Shen, Y., et al., *BMN 673, a novel and highly potent PARP1/2 inhibitor for the treatment of human cancers with DNA repair deficiency.* Clin Cancer Res, 2013. **19**(18): p. 5003-15.

12. Patel, A.G., et al., *Enhanced Killing of Cancer Cells by Poly(ADP-ribose) Polymerase Inhibitors and Topoisomerase I Inhibitors Reflects Poisoning of Both Enzymes.* Journal of Biological Chemistry, 2012. **287**(6): p. 4198-4210.

13. Samol, J., et al., *Safety and tolerability of the poly(ADP-ribose) polymerase (PARP) inhibitor, olaparib (AZD2281) in combination with topotecan for the treatment of*

patients with advanced solid tumors: a phase I study. Investigational New Drugs, 2012. **30**(4): p. 1493-1500.

14. Kummar, S., et al., *Phase I Study of PARP Inhibitor ABT-888 in Combination with Topotecan in Adults with Refractory Solid Tumors and Lymphomas*. Cancer Research, 2011. **71**(17): p. 5626-5634.
15. Weiss, G.J., et al., *First-in-human phase 1/2a trial of CRLX101, a cyclodextrin-containing polymer-camptothecin nanopharmaceutical in patients with advanced solid tumor malignancies*. Invest New Drugs, 2013. **31**(4): p. 986-1000.
16. Eliasof, S., et al., *Correlating preclinical animal studies and human clinical trials of a multifunctional, polymeric nanoparticle*. Proc Natl Acad Sci U S A, 2013. **110**(37): p. 15127-32.
17. Matsumura, Y. and H. Maeda, *A new concept for macromolecular therapeutics in cancer chemotherapy: mechanism of tumoritropic accumulation of proteins and the antitumor agent smancs*. Cancer Res, 1986. **46**(12 Pt 1): p. 6387-92.
18. Farmer, H., et al., *Targeting the DNA repair defect in BRCA mutant cells as a therapeutic strategy*. Nature, 2005. **434**(7035): p. 917-21.
19. McCabe, N., et al., *Deficiency in the repair of DNA damage by homologous recombination and sensitivity to poly(ADP-ribose) polymerase inhibition*. Cancer Res, 2006. **66**(16): p. 8109-15.
20. Menear, K.A., et al., *Novel alkoxybenzamide inhibitors of poly(ADP-ribose) polymerase*. Bioorg Med Chem Lett, 2008. **18**(14): p. 3942-5.
21. Sousa, F.G., et al., *Alterations of DNA repair genes in the NCI-60 cell lines and their predictive value for anticancer drug activity*. DNA Repair, 2015. **28**: p. 107-115.
22. Zoppoli, G., et al., *Putative DNA/RNA helicase Schlafen-11 (SLFN11) sensitizes cancer cells to DNA-damaging agents*. Proc Natl Acad Sci U S A, 2012. **109**(37): p. 15030-5.
23. Tang, S.W., et al., *SLFN11 is a transcriptional target of EWS-FLI1 and a determinant of drug response in Ewing's sarcoma*. Clin Cancer Res, 2015. **21**(18): p. 4184-4193.
24. Eisenhauer, E.A., et al., *New response evaluation criteria in solid tumours: revised RECIST guideline (version 1.1)*. Eur J Cancer, 2009. **45**(2): p. 228-47.
25. Rustin, G.J., et al., *Re: New guidelines to evaluate the response to treatment in solid tumors (ovarian cancer)*. J Natl Cancer Inst, 2004. **96**(6): p. 487-8.
26. Scher, H.I., et al., *Design and end points of clinical trials for patients with progressive prostate cancer and castrate levels of testosterone: recommendations of the Prostate Cancer Clinical Trials Working Group*. J Clin Oncol, 2008. **26**(7): p. 1148-59.
27. Bubley, G.J., et al., *Eligibility and response guidelines for phase II clinical trials in androgen-independent prostate cancer: recommendations from the Prostate-Specific Antigen Working Group*. J Clin Oncol, 1999. **17**(11): p. 3461-7.

28. Vergote, I., et al., *Re: new guidelines to evaluate the response to treatment in solid tumors [ovarian cancer]. Gynecologic Cancer Intergroup.* J Natl Cancer Inst, 2000. **92**(18): p. 1534-5.

13 APPENDICES

13.1 APPENDIX A: PERFORMANCE STATUS CRITERIA

**ECOG Performance Status Scale**		**Karnofsky Performance Scale**	
Grade	Descriptions	Percent	Description
0	Normal activity. Fully active, able to carry on all pre-disease performance without restriction.	100	Normal, no complaints, no evidence of disease.
		90	Able to carry on normal activity; minor signs or symptoms of disease.
1	Symptoms, but ambulatory. Restricted in physically strenuous activity, but ambulatory and able to carry out work of a light or sedentary nature (e.g., light housework, office work).	80	Normal activity with effort; some signs or symptoms of disease.
		70	Cares for self, unable to carry on normal activity or to do active work.
2	In bed <50% of the time. Ambulatory and capable of all self-care, but unable to carry out any work activities. Up and about more than 50% of waking hours.	60	Requires occasional assistance, but is able to care for most of his/her needs.
		50	Requires considerable assistance and frequent medical care.
3	In bed >50% of the time. Capable of only limited self-care, confined to bed or chair more than 50% of waking hours.	40	Disabled, requires special care and assistance.
		30	Severely disabled, hospitalization indicated. Death not imminent.
4	100% bedridden. Completely disabled. Cannot carry on any self-care. Totally confined to bed or chair.	20	Very sick, hospitalization indicated. Death not imminent.
		10	Moribund, fatal processes progressing rapidly.
5	Dead.	0	Dead.

13.2 APPENDIX B: LIST OF DRUGS THAT MAY HAVE POTENTIAL CYP3A4 INTERACTIONS

13.2.1 CYP3A4 Substrates

Albuterol
Alfentanil
Alprazolam
Amiodarone
Amlodipine
Amprenavir
Aprepitant
Aripiprazole
Atazanavir
Atorvastatin
Benzphetamine
Bisoprolol
BortezomibBosentan
Bromazepam
Bromocriptine
Budesonide
Buprenorphine
Buspirone
Busulfan
Carbamazepine
Cerivastatin
Chlordiazepoxide
Chloroquine
Chlorpheniramine
Cilostazol
Cisapride
Citalopram
Clarithromycin
Clobazam
Clonazepam
Clorazepate
Cocaine
Colchicine
Conivaptan
Cyclophosphamide
Cyclosporine
Dantrolene
Dapsone
Dasatinib
Delavirdine
Diazepam
Dihydroergotamine
Diltiazem
Disopyramide
Docetaxel
Doxepin
Doxorubicin
Doxycycline
Efavirenz
Eletriptan
Enalapril
Eplerenone
Ergoloid mesylates
Ergonovine
Ergotamine
Erythromycin
Escitalopram
Estradiol
Estrogens, conj., synthetic
Estrogens, conj., equine
Estrogens, conj., esterified
Estrone
Estropipate
Ethinyl estradiol
Ethosuximide
Etoposide
Exemestane
Felbamate
Felodipine
Fentanyl
Flurazepam
Flutamide
Fluticasone
Fosamprenavir
Gefitinib
Haloperidol
Ifosfamide
Imatinib
Indinavir
Irinotecan
Isosorbide
Isosorbide dinitrate
Isosorbide mononitrate
Isradipine
Itraconazole
Ketamine
Ketoconazole
Lansoprazole
Letrozole
Levonorgestrel
Lidocaine
Losartan
Lovastatin
Medroxyprogesterone
Mefloquine
Mestranol
Methadone
Methylergonovine
Methysergide
Miconazole
Midazolam
Miglustat
Mirtazapine
Modafinil
Montelukast
Moricizine
Nateglinide
Nefazodone
Nelfinavir
Nevirapine
Nicardipine
Nifedipine
Nimodipine
Nisoldipine
Norethindrone
Norgestrel
Ondansetron
Paclitaxel
Pergolide
Phencyclidine
Pimozide
Pipotiazine
Primaquine
Progesterone
Quetiapine
Quinidine
Rabeprazole
Ranolazine
Repaglinide
Rifabutin
Ritonavir
Salmeterol
Saquinavir
Sibutramine
Sildenafil
Simvastatin
Sirolimus
Spiramycin
Sufentanil
Sunitinib
Tacrolimus
Tamoxifen
Tamsulosin
Telithromycin
Teniposide
Tetracycline
Theophylline
Tiagabine
Ticlopidine
Tipranavir
Tolterodine
Toremifene
Trazodone
Triazolam
Trimethoprim
Trimipramine
Troleandomycin
Vardenafil
Venlafaxine
Verapamil
Vinblastine
Vincristine
Vinorelbine
Zolpidem
Zonisamide
Zopiclone

13.2.2 CYP3A4 Inhibitors

Acetominophen
Acetazolamide
Amiodarone
Amlodipine
Amprenavir
Anastrozole
Aprepitant
Atazanavir
Atorvastatin
Azelastine
Azithromycin
Betamethasone
Bortezomib
Bromocriptine
Caffeine
Cerivastatin
Chloramphenicol
Chlorzoxazone
Cimetidine
Ciprofloxacin
Cisapride
Clarithromycin
Clemastine
Clofazimine
Clotrimazole
Clozapine
Cocaine
Conivaptan
Cyclophosphamide
Cyclosporine
Danazol
Dasatinib
Delavirdine
Desipramine
Dexmedetomidine
Diazepam
Diclofenac
Dihydroergotamine
Diltiazem
Disulfiram
Docetaxel
Doxorubicin
Doxycycline
Drospirenone
Efavirenz
Enoxacin
Entacapone
Ergotamine
Erythromycin
Ethinyl estradiol
Etoposide
Felodipine
Fentanyl
Fluconazole
Fluoxetine
Fluvastatin
Fluvoxamine
Fosamprenavir
Glyburide
Grapefruit juice (1)
Haloperidol
Hydralazine
Ifosfamide
Imatinib
Indinavir
Irbesartan
Isoniazid
Isradipine
Itraconazole
Ketoconazole
Lansoprazole
Lidocaine
Lomustine
Losartan
Lovastatin
Mefloquine
Mestranol
Methadone
Methimazole
Methoxsalen
Methylprednisolone
Metronidazole
Miconazole
Midazolam
Mifepristone
Mirtazapine
Mitoxantrone
Modafinil
Nefazodone
Nelfinavir
Nevirapine
Nicardipine
Nifedipine
Nisoldipine
Nizatidine
Norfloxacin
Olanzapine
Omeprazole
Orphenadrine
Oxybutynin
Paroxetine
Pentamidine
Pergolide
Phencyclidine
Pilocarpine
Pimozide
Pravastatin
Prednisolone
Primaquine
Progesterone
Propofol
Propoxyphene
Quinidine
Quinine
Quinupristin
Rabeprazole
Ranolazine
Risperidone
Ritonavir
Saquinavir
Selegiline
Sertraline
Sildenafil
Sirolimus
Sulconazole
Tacrolimus
Tamoxifen
Telithromycin
Teniposide
Testosterone
Tetracycline
Ticlopidine
Tranylcypromine
Trazodone
Troleandomycin
Valproic acid
Venlafaxine
Verapamil
Vinblastine
Vincristine
Vinorelbine
Voriconazole
Zafirlukast
Ziprasidone

13.2.3 CYP3A4 Inducers

Aminoglutethimide
Carbamazepine
Fosphenytoin
Nafcillin
Nevirapine
Oxcarbazepine
Pentobarbital
Phenobarbital
Phenytoin
Primidone
Rifabutin
Rifampin
Rifapentine
St. John‟s wort (2)

When drugs classified as „substrates‟ are co-administered with olaparib, there is the potential for higher concentrations of the “substrate”. When olaparib is co-administered with compounds classified as „inhibitors‟,

increased plasma concentrations of olaparib is the potential outcome. The co-administration of „inducers" would potentially lower plasma olaparib concentrations.

Note: Adapted from Cytochrome P450 Enzymes: Substrates, Inhibitors, and Inducers. In: Lacy CF, Armstrong LL, Goldman MP, Lance LL eds. Drug Information Handbook 15TH ed. Hudson, OH; LexiComp Inc. 2007: 1899-1912.
Only major substrates and effective inducers are listed.
Additional information for drug interactions with cytochrome P450 isoenzymes can be found at http://medicine.iupui.edu/flockhart/.
(1) Malhotra *et al*. (2001). Clin Pharmacol Ther. 69:14-23.
(2) Mathijssen *et al*. (2002). J Natl Cancer Inst. 94:1247-1249.

Frye *et al*. (2004). Clin Pharmacol Ther. 76:323-329.

13.3 APPENDIX C: PATIENT'S PILL DIARY: OLAPARIB

Today's date ________________________________
Patient Name________________________________ Patient Study ID _______________________
Cycle #__________
(*initials acceptable for patient's name)*

INSTRUCTIONS TO THE PATIENT:

1. Complete one form for each cycle (28 days).
2. You will take ___ tablets twice a day 12 hours apart on days 3 – 13 and days 17 -26. You must take the tablets with a large glass of water. A light snack (biscuits/ toast) is also recommended to help reduce nausea.
3. Record the date, the number of tablets you took, and when you took them.
4. If you have any comments or notice any side effects, please record them in the Comments column.
5. Please bring your pill bottle and this form to your physician when you go for your next appointment.

**DAY**	**DATE**	**# TABLETS AND WHEN TAKEN: OLAPARIB**<br>**AM PM**	**COMMENTS (side effects or missed doses)**
1		Do not take olaparib	
2		Do not take olaparib	
3		7AM<br>7PM	
4		7AM<br>7PM	
5		7AM<br>7PM	
6		7AM<br>7PM	
7		7AM<br>7PM	

8		7AM<br>7PM	
9		7AM<br>7PM	
10		7AM<br>7PM	
11		7AM<br>7PM	
12		7AM<br>7PM	
13		7AM<br>No PM	
14		Do not take olaparib	
15		Do not take olaparib	
16		Do not take olaparib	
17		7AM<br>7PM	
18		7AM<br>7PM	
19		7AM<br>7PM	
20		7AM<br>7PM	
21		7AM<br>7PM	

22		7AM<br>7PM	
23		7AM<br>7PM	
24		7AM<br>7PM	
25		7AM<br>7PM	
26		7AM<br>No PM	
27		Do not take olaparib	
28		Do not take olaparib	

Patient's Signature: ____ Date: _________________________

The Study Team will complete this section:
1. Date patient started protocol treatment_______________ Date patient was removed from study ____________________
2. Patient's planned daily dose_______________________ Total number of pills taken this month ___________________
Physician/Nurse Signature ____

NCI Protocol #: *16C0107*

Title: A Phase I/II Trial of CRLX101, a Nanoparticle Camptothecin with Olaparib in Patients with Relapsed/Refractory Small Cell Lung Cancer

Principal Investigator: Anish Thomas, MBBS, M.D.
Thoracic and Gastrointestinal Oncology Branch (TGIB)
Center for Cancer Research (CCR)
National Cancer Institute (NCI)

High Level Summary of Protocol Changes

	Protocol version date	Approval date	Summary
Initial	4/13/2016	4/20/2016	NA
Amendment A	3/18/2017	4/3/2017	The purpose of this amendment is to change the dose escalation schema to escalate olaparib from 200 mg to 300 mg between dose levels 3 and 4 (compared with 200 mg to 250 mg proposed earlier), clarify eligibility requirements (CYP inhibitors, prior transfusion), clarify management of CRLX101 infusion reactions, update adverse event profile of CRLX101 and administrative changes
Amendment B	5/25/2017	5/29/2017	This protocol amendment is being submitted in response to filed deviations with the reference numbers 369116 and 368967 to revise the duration of CRLX101 infusion from 120 minutes to 60 minutes. Although not mandated by IRB, the protocol also includes changes made in response to reported protocol deviation number 368707 to increase the window of pre-medication with 3 drug classes (corticosteroid, antihistamine and H2 antagonist) up to 120 minutes.
Amendment C	8/16/2017	8/31/2017	The purpose of this amendment is to add 2 expansion cohorts: one for the patients with metastatic or advanced urothelial carcinoma (UC) with selected DNA-gene defects for a total of 35 patients and the second cohort for castrate resistant metastatic prostate cancer (mCRPC) for a total of 25 patients. The expansion cohorts are being added to determine overall response rate of CRLX101

			plus olaparib in patients with mCRPC and urothelial carcinoma. Manufacturer of CRLX101 has changed to BlueLink as Cerulian Pharma Inc has sold its shares.
Amendment D	1/19/2018	2/18/2018	Updates are being made to key study personnel as well as to include other clarifications, administrative, editorial and template changes.
Amendment E	2/26/2018	03/26/20818	The amendment is being submitted to define RP2D since Phase I part of the trial is now completed as well as to clarify the dose modification for a starting dose of olaparib. Furthermore, the amendment includes an additional timepoint of hair collection and makes hair collection enrolled on Phase II part of the study optional. In addition to the cycle 1 hair collections, hair follicles may be collected at the end of each cycle at approximately 24 hours after last dose of olaparib. This will provide a PD readout of phosphorylation of H2AX after olaparib alone.
Amendment F	4/9/2018	5/21/2018	The purpose of this amendment is to clarify several matters that were identified during the site initiation visit for the expansion cohorts pertaining to screening procedures, I/E criteria, CRLX101 infusion duration (60 minutes to 60-75 min)
Amendment G	7/23/2018	7/24/2018	The purpose of this amendment is to add clarifications regarding the prostate cancer cohort. • The time point for LDH, creatine kinase, uric acid, and total protein was changed from Day 1 to Cycle 2 Day 1; and the window for on-study assessments was increased to ±7 days to facilitate
Amendment H	1/3/2019	2/6/2019	For the SCLC phase II cohort, we have defined the phase II dose but not enrolled any patients. Although we were initially thinking of stratifying patients based on platinum-sensitivity previously, that would require 40 patients and a prolonged period of accrual. In order to abbreviate the trial duration and to detect a signal of activity sooner, we propose to amend the protocol to study both platinum sensitive and resistant patients together. Additionally, a recent study with more contemporary data shows that the previously set bar may have been too high (Pietanza JCO).
Amendment I	2/12/2019	3/1/2019	The purpose of this amendment is to add some clarification in the patient pill diary so as to avoid confusion for patients. In addition, information regarding the management of grade ≥ 3 neutropenia and grade ≥ 2 thrombocytopenia is being added to an additional section to ensure procedures are done by the research team.
Amendment J	5/20/2019	5/29/2019	The purpose of this amendment is to add the requirement that an ECG be done within 1-3 hours post-infusion for the first two cycles of CRLX101 for patients with any conduction abnormality

			on baseline ECG per IRB request (PRF 530972). Clarification is being provided for study lab assessments and for the exclusion of active pneumonitis.
Amendment K	5/30/2019	6/4/2019	The purpose of this amendment is to ensure the hemoglobin inclusion criteria is consistent within all cohorts. The holidays for Epic Sciences are also being updated.
Amendment L	7/8/2019	8/6/2019	The purpose of this amendment is to update the consent to include genetic summary results language. In addition, safety reporting requirements are being updated in the protocol per policies 801 and 802. Correction is being made for lab procedures for the mCRPC cohort.
Amendment M	10/10/2019	12/2/2019	The purpose of this amendment is to update the study calendar to allow monthly visits for patients that are being treated with Olaparib only. Additionally, clarification has been made that prostate cancer patients who have received previous radiation therapy may be included in the study (with a 2 week wash out period).
Amendment N	11/2/2020	12/15/2020	The purpose of this amendment is to switch the CRLX101 manufacturer to Ellipses and update clinical development information per the updated IB. Administrative changes include updating registration procedures language, sample storage and tracking information, radiation risk information and informed consent process and documentation.
Amendment	3/18/2021	4/7/2021	The purpose of this amendment is to add new risks associated with Olaparib per investigator brochure (IB) version 20.
Amendment	7/27/2021	9/9/2021	The purpose of this amendment is to update the protocol to clarify that the accrual for the mCRPC cohort has been stopped. Accrual to this cohort has been slow given the recent FDA approval of olaparib for the treatment of prostate cancer. Additionally, underlying conditions such as history of pelvic irradiation or surgery predispose the prostate cohort to cystitis and hematuria.
Amendment	10/6/2021	11/2/2021	The purpose of this amendment is to update CTCAE version from 4 to 5 per sponsor request.
Amendment	1/31/2022	3/29/2022	The purpose of this amendment is to update risks associated with olaparib per new investigator brochure (IB version 21, 01/31/2022). The notable changes are the addition of venous thromboembolism as an adverse drug reaction and embolism/pulmonary embolism as a serious adverse reaction. Changes in frequency categories were also made to the consent for consistency with the new IB. Per the principal investigator, there are no new risks that qualify as unanticipated problems.

Amendment	8/17/2022	10/4/2022	The purpose of this amendment is to update the protocol to reflect accrual closure for the urothelial carcinoma cohort. Accrual to this cohort has been slow due to competing protocols for the same patient population. The decision was made to close cohort accrual on this trial to prioritize other current and upcoming studies. Dear investigator letter information has been added addressing the intended withdrawal of olaparib for the treatment of adult patients with deleterious or suspected deleterious germline BRCA-mutated (gBRCAm) advanced ovarian cancer who have been treated with three or more prior lines of chemotherapy.
Amendment	1/20/2023	2/21/2023	The purpose of this modification is to update the contraception use language in the protocol and consent per the new olaparib investigator brochure version 21.2 (dated 10/04/2022). Per this new IB, contraception use for men and their partners has been updated to clarify that hormonal and barrier method or total/true abstinence are to be used for at least 3 months after the last dose of study drugs.
Amendment	3/11/2024	4/1/2024	The purpose of this modification is to replace references to Trepel lab, who is no longer part of the study, with the DTB Clinical Translational Unit.
Amendment	8/16/2024	10/15/2024	The purpose of this modification is to add on-study screening due to the upcoming deactivation of 01C0129. Possible recruitment strategies to boost enrollment are being added per the last continuing review recommendation.

13
